# Activation-Free Sulfonyl Fluoride Probes for Fragment Screening

**DOI:** 10.3390/molecules28073042

**Published:** 2023-03-29

**Authors:** László Petri, Péter Ábrányi-Balogh, Noémi Csorba, Aaron Keeley, József Simon, Ivan Ranđelović, József Tóvári, Gitta Schlosser, Dániel Szabó, László Drahos, György M. Keserű

**Affiliations:** 1 Medicinal Chemistry Research Group, Research Centre for Natural Sciences, Magyar Tudósok Krt. 2, 1117 Budapest, Hungary; petri.laszlo@ttk.hu (L.P.); abranyi-balogh.peter@ttk.hu (P.Á.-B.); csorba.noemi@ttk.hu (N.C.); aaron.keeley1989@gmail.com (A.K.); simon.jozsef@ttk.hu (J.S.); 2 National Laboratory for Drug Research and Development, Research Centre for Natural Sciences, Magyar Tudósok Krt. 2, 1117 Budapest, Hungary; 3 Department of Organic Chemistry and Technology, Budapest University of Technology and Economics, Szent Gellért tér 4, 1111 Budapest, Hungary; 4 Research Centre for Natural Sciences, MS Metabolomics Research Group, Magyar Tudósok Krt. 2, 1117 Budapest, Hungary; 5 KINETO Lab Ltd., Zápor u. 55, 1032 Budapest, Hungary; ivan.randelovic@kinetolab.hu; 6 Department of Experimental Pharmacology and National Tumor Biology Laboratory POB 21, National Institute of Oncology, 1525 Budapest, Hungary; tovari.jozsef@oncol.hu; 7 MTA-ELTE Lendület Ion Mobility Mass Spectrometry Research Group, Institute of Chemistry, ELTE Eötvös Loránd University, Pázmány Péter Sétány 1/A, 1117 Budapest, Hungary; gitta.schlosser@ttk.elte.hu; 8 MS Proteomics Research Group, Research Centre for Natural Sciences, Magyar Tudósok Krt. 2, 1117 Budapest, Hungary; szabo.daniel@ttk.hu (D.S.); drahos.laszlo@ttk.hu (L.D.)

**Keywords:** chemical probe, covalent fragment, electrophilic warhead, fragment screening, sulfonyl fluoride, targeted covalent inhibitor

## Abstract

SuFEx chemistry is based on the unique reactivity of the sulfonyl fluoride group with a range of nucleophiles. Accordingly, sulfonyl fluorides label multiple nucleophilic amino acid residues, making these reagents popular in both chemical biology and medicinal chemistry applications. The reactivity of sulfonyl fluorides nominates this warhead chemotype as a candidate for an external, activation-free general labelling tag. Here, we report the synthesis and characterization of a small sulfonyl fluoride library that yielded the 3-carboxybenzenesulfonyl fluoride warhead for tagging tractable targets at nucleophilic residues. Based on these results, we propose that coupling diverse fragments to this warhead would result in a library of sulfonyl fluoride bits (SuFBits), available for screening against protein targets. SuFBits will label the target if it binds to the core fragment, which facilitates the identification of weak fragments by mass spectrometry.

## 1. Introduction

Sulfonyl fluoride-based SuFEx chemistry was identified as a next generation click-chemistry approach by the Sharpless group in the mid-2010s [1,2,3,4]. Since then, sulfonyl fluorides have been used in a vast array of chemical biology and medicinal chemistry applications due to their reactivity against nucleophilic residues [5,6,7,8,9]. In particular, sulfonyl fluorides are known to label tyrosine, threonine, serine, lysine, cysteine and histidine residues, and therefore, when integrated into bioactive molecules, they are powerful tools for the irreversible labelling and modulation of protein targets [10,11,12]. Sulfonyl fluorides are considered as synthetically accessible, relatively stable and low-toxicity reagents, and their applications can be found in nearly all areas of modern chemistry, including syntheses, chemical biology, drug discovery and materials chemistry [1]. Notably, it has also been reported recently that sulfonyl fluorides are applicable in robust multicomponent syntheses [5,13,14], providing a viable option for generating large libraries equipped with this warhead. 

Covalent labelling of proteins is usually residue specific. Generally, applied warheads are typically Michael acceptors or other cysteine-targeting electrophilic functional groups, but serine, lysine and tyrosine are also considered as potential targets [15]. There are several specific applications, including SuFEx-based covalent inhibition, targeting mostly lysine or tyrosine nucleophilic residues [16,17,18,19,20,21,22,23]. Recently published comprehensive profiling studies of sulfonyl fluorides [24,25] provide confidence that sulfonyl fluoride bits (SuFBits) could be a valuable addition in the general toolbox of chemical biology, complementing the cysteine-targeting covalent approaches.

Side-chain-independent protein labelling is mostly achieved by photoaffinity tags [26,27,28]. The reactive functional groups (diazirines, benzophenones and azides) in this approach are activated by UV irradiation and react through a radical, carbene, diazo or nitrene intermediate with nearby residues. Although it is known that in the case of diazirines there is a preference towards negatively charged carboxylic acid residues (Asp and Glu) [29], photoaffinity labelling can still be considered as a promiscuous labelling approach. However, photoactivation, especially with UV light, might cause the degradation of targeted biomolecules or even the probe and requires equipment that assures reproducibility and efficacy. In a recent study of GSK diazirine photoaffinity tag was used to form a library of PhotoAffinity Bits (PhABits), as the basis of a photoaffinity-based fragment screening platform [30]. This technology, however, is restricted to photostable targets and photo-resistant fragments that might limit the application domain and the chemical space covered. Furthermore, filtering out photoactive and photosensitive fragments might complicate the screening process. These drawbacks could be outplayed with a warhead that labels multiple amino acid residues and thus can be clicked to the targeted protein without the need for outer activation. Based on the former results, sulfonyl fluorides could be considered for this application; however, their proper characterization and the options to determine their reactivity is still an emerging area [25]. Our final objective is to develop a similar approach to PhaBits while avoiding the need for photoactivation by applying the sulfonyl fluoride warhead. Developing the basis of the SuFBits platform, we first characterized and selected an optimized sulfonyl fluoride labelling tag that labels multiple residues and can be easily coupled to fragments.

## 2. Results and Discussion

**Figure 1:**
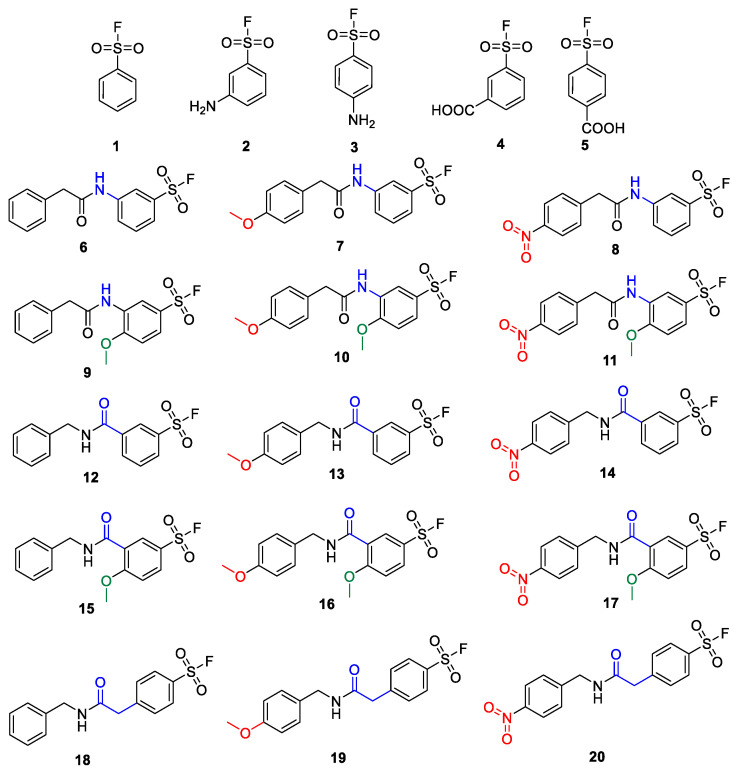
Members of the aryl sulfonyl fluoride library. Blue atoms are the attachment points between the warhead and the variable fragment. Red and green groups are electron donating (OMe) or electron withdrawing groups (NO_2_) to reveal their possible impact on reactivity.

Developing the SuFBits fragment screening platform, we first required an appropriate tag that contains the sulfonyl fluoride warhead and a suitable attachment point/functionality to enable general application in library synthesis. We imagined that this functional group should be easily available for robust reactions, e.g., acylation or well-established multicomponent reactions. We have taken commercially available aryl sulfonyl fluorides that are modified in the *para* or *meta* position with an amino or carboxylic acid functional group (**1**–**5**) as the variable fragment binding element (Figure 1). We have synthesized derivatives of these with or without additional substituents on the phenyl ring or more distant from the warhead (**6**–**17**), and with or without a methylene spacer as well (**18**–**20**), separating the amide group from the aromatic core. The additional substituents attached to the phenylsulfonyl fluoride were benzyl, 4′-methoxybenzyl and 4′-nitrobenzyl groups in order to check the possible effect of a distant electron donating or electron withdrawing group on the reactivity.

**Figure 2:**
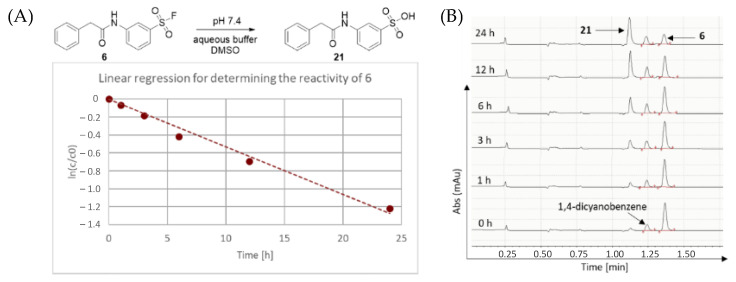
(**A**) Representative chemical reaction of the intrinsic reactivity assay with representative linear regression resulting in a k value for the **6**→**21** transformation and (**B**) representative HPLC chromatograms of the **6**→**21** reaction.

First, we characterized the intrinsic reactivity of the library members (Table 1). Compounds were added in DMSO to PBS buffer (pH 7.4) together with the internal standard 1,4-dicyanobenzene. The vial was mixed, then analysed by HPLC-MS in 1, 2, 3, 6, 12 and 24 h time points (Figure 2). In all cases, hydrolysis of the corresponding sulfonic acid could be observed (Figure 2). These data provided the pseudo first order kinetic rate constant (k) and aqueous stability half-lives (t_1/2_) after linear regression (Table 1 and Figure 2).

In comparison to benzenesulfonyl fluoride (**1**), the amino (**2**,**3**) and the carboxylic acid substituents (**4**,**5**) caused an increased half-life, suggesting higher stability and lower aqueous reactivity (Table 1). The less reactive one was 4-aminobenzenesulfonyl fluoride (**3**), followed by the 3-amino derivative (**2**), while among the carboxylic acids, the meta-substituted compound (**4**) was more stable. These differences might be explained by the electron donating character of the amino group contrary to the electron withdrawing effect of the acids. Based on these results, 3-aminobenzenesulfonyl fluoride (**2**) seemed to be the better choice for further library design than the less reactive *para* analogue (**3**). Among the carboxylic acids, both seemed advantageous, but the *meta* substitution (**4**) had moderate reactivity that might be considered a better choice. Acylation on the *meta* amino group with phenylacetic acids and the addition of a methoxy to the benzenesulfonyl core (**6**–**8** and **9**–**11**, respectively) increased the reactivity to t_1/2_ = 10–19 h, which was generally not effected significantly by the substituents. This might also be an advantage during library design if the reactivity of the covalent warhead is stable regardless of the non-covalent part of the molecule. On the contrary, when acylating benzylamines with 3-carboxybenzenesulfonyl fluoride (**12**–**14**), the reactivity increased to t_1/2_ = 4–5 h regardless of the substituents.

Interestingly, the additional methoxy group led to higher half-lives for compounds **15** and **16**, while for the nitro-substituted sulfonyl fluoride (**17**), no change could be seen. With these experiments, we show that to achieve constant or flexible reactivity, either the amino (**2**) or the carboxy warhead (**4**) can be chosen during the design of a fragment library. In order to detach the effect of the substituents and check other types of warhead connection through a longer linker, we have used 2-(4-(fluorosulfonyl)phenyl)acetic acid for acylating benzylamines. Compounds **18**–**20** provided the lowest half-life values (t_1/2_ = 1–4 h), suggesting the highest reactivity in parallel the lowest aqueous stability, which might limit the application of these compounds to rapid labelling approaches. We also presume that the large range of reactivity and stability of compounds **6**–**20** (t_1/2_ = 1–38 h) might enable fine tuning of the reaction with the target of interest. Notably, these results were in accordance with similar research published recently naming similar compounds to *meta*-carboxy derivatives such as **12**–**20**, which exhibit desirable properties for applications in biological systems [25].

**Figure 3:**
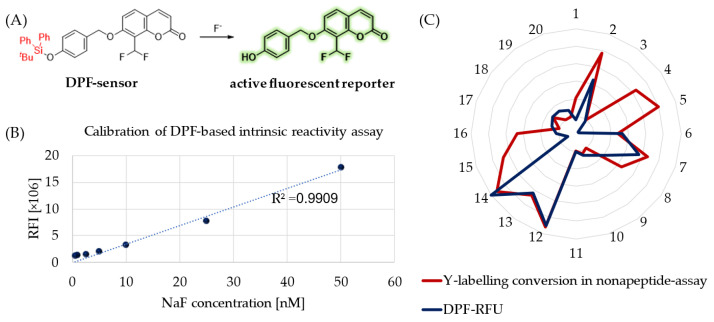
(**A**) Fluorogenic reaction of the DPF sensor molecule in the presence of fluoride ions; (**B**) calibration of assay sensitivity with NaF solutions and (**C**) the measured activity as relative fluorescence unit (RFU) for compounds **1**–**20** and compared to the tyrosine labelling conversion obtained by the nonapeptide assay.

Besides hydrolytic characterization, we aimed to measure the intrinsic reactivity of sulfonyl fluoride probes against a tyrosine surrogate in physiological buffer (PBS, pH = 7.4). Therefore, we developed a 96-well-plate-based spectroscopic analysis applying DPF as a sensitive fluoride sensor molecule (Figure 3A) [31]. This molecule is a fluoride-dependent on–off fluorogenic probe, where a 4-(*tert*-butyldiphenylsilyloxy) trigger group is attached to the latent reporter coumarin. Fluoride ions induce the removal of a silyl protecting trigger group on DPF (Figure 3A). After calibration of the chromogenic assay with a NaF concentration series (Figure 3B), we measured the amount of the released fluoride in the reaction of SuFBits with *N*-acetyl tyrosine as a protein surrogate. After 15 minutes of incubation at room temperature, the release of the 7-hydroxy-8- formylcoumarin was monitored by measuring the fluorescence endpoint at 𝜆_ex_ = 360 nm and 𝜆_em_ = 445 nm. This measured-reactivity-related fluorescence intensity (Supplementary Table S1) was then normalized and used as relative fluorescence units (RFU) to describe the reactivity of the library members.

To compare the assay outcome to a more complex surrogate, we performed a nonapeptide assay (KGDYHFPIC) developed in our group [32] and took the direct conversion of the tyrosine-specific labelling events (Figure 3C). We observed here a significant correlation between these orthogonal methods describing the tyrosine reactivity of the SuFBit probes. Interestingly, compounds **4**, **5**, **15** and **16** resulted in significant tyrosine labelling of the nonapeptide; however, they were completely unreactive based on the DPF assay. With all the other probes, the two parallel methods corresponded very well, resulting in probes **12** and **14** as the two strongest consensus hits (Figure 3C). 

**Figure 4:**
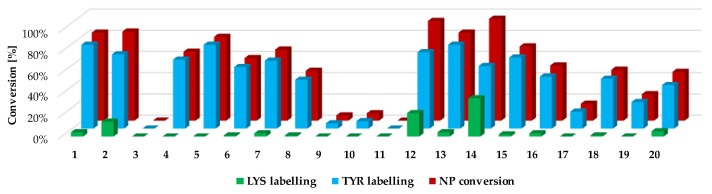
Nonapeptide conversion and amino acid labelling (selectivity/promiscuity) data for the phenyl-derived sulfonyl fluorides (**1**–**20**).

Next, we investigated the residue specificity of the labelling reaction. The nonapeptide contains several nucleophilic amino acid residues (Lys, His, Tyr and Cys); therefore, it is a useful surrogate for the estimation of labelling selectivity/promiscuity (number and proportion of labelled amino acid residues). In this assay, we detected labelling only on Tyr and Lys side chains, and in all cases the cysteines dimerized to disulphide bridges, forming nonapeptide dimers. This phenomenon is unique among the electrophilic chemotypes we have investigated up to now, although the assay conditions were the same as before [32]. It should be noted that single lysine labelling was not observed; lysines were labelled only together the Tyr residues, suggesting that at first Tyr is labelled and lysine is the secondary target. Considering the reported promiscuity of sulfonyl fluorides, labelling multiple residues, including Cys, His, Thr and Ser, is expected in a protein environment. In the nonapeptide reactivity screening (Figure 4 and Supplementary Table S1), phenylsulfonyl fluoride (**1**) showed 83% conversion and 4-aminophenylsulfonyl fluoride (**3**) did not label the nonapeptide, while conversion was 84% in the case of 3-aminophenylsulfonyl fluoride (**2**). The carboxylic acid substituents led to similar trends as that of the intrinsic reactivity assay, as sulfonyl fluoride **4** was less reactive than **5** (65% and 79%, respectively). Moving forward to the larger analogues, the lowest labelling efficacy was observed for **18**–**20** (25–48% conversion), where the phenylsulfonyl fluoride is attached through an acetyl linker in the *para* position. This might be explained by the high rate of the aqueous hydrolysis side reaction. Comparing this group to **12**–**14**, where the carboxyl group is directly attached to the phenylsulfonyl core in the *meta* position, a significant increase in reactivity could be observed (83–96% conversion), resulting in the most reactive compounds from the whole library. Sulfonyl fluorides **12**–**14** also had higher reactivity than that of the free carboxylic acid **4** (65%). Thus, it could be concluded that direct attachment in the *meta* position is advantageous, and an aqueous half-life of 4–5 h is still tolerated in labelling reactions. Notably, the addition of a methoxy group to the phenylsulfonyl fluoride warhead significantly decreased the reactivity (**15**–**17**, 16–70%) compared to **12**–**14**. When the warhead was attached using 3-aminophenylsulfonyl fluoride (**6**–**8**), the conversion was moderate (47–67%), and adding the methoxy group similarly resulted in lower values; in particular, the compounds almost lost their ability for labelling (**9**–**12**, 0–7%). Comparing **6**–**11** to **2** shows that increasing the size of **2** decreases the nonapeptide reactivity and thus the Tyr affinity. The nitro substituents distant from the sulfonyl fluoride (**8**, **11**, **14**, **17** and **20**) generally showed a decrease in the reactivity, while there was no regularity in the case of the methoxy-substituted analogues (**7**, **10**, **13**, **16** and **19**). It could be concluded that coupling through the carboxyl group results in more efficient labelling (average nonapeptide conversion 69 ± 30%) than coupling through the amine (31 ± 30%). The carboxyl modification keeps the originally observed reactivity and amino acid affinity of the phenylsulfonyl warhead. This might be rationalized by the similar electronic properties of amides and carboxylic acids, and the very different ones of amines and amide nitrogens. A recent work reporting a multicomponent synthesis of sulfonyl fluorides using 3-carboxyphenylsulfonyl fluoride gives further support to our observation [15].

**Figure 5:**
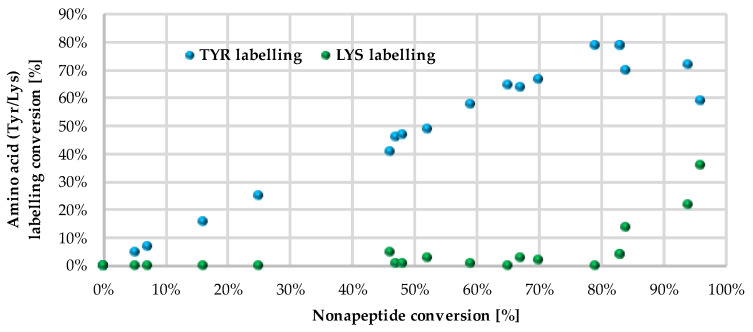
Correlation between nonapeptide conversion and amino acid labelling percentage (Tyr—blue, Lys—green).

We followed up the oligopeptide assay by analysing the promiscuity of sulfonyl fluorides. Probe **14** labelled both nucleophilic residues (double labelling on the nonapeptide for Tyr and Lys) with significant yield (Figure 4, blue and green columns, Supplementary Table S1 and Supplementary Figure S2). The compounds with the highest reactivity usually showed 60–80% Tyr labelling, in addition to 10–40% Lys labelling with single and double adducts. Only **12** and **14** provided a considerate conversion of lysine labelling (22% and 36%, respectively). In general, sulfonyl fluorides reacted preferentially with Tyr and only the most reactive ones could label Lys. Comparing the nonapeptide conversions to the amino acid labelling percentage, the increase in Tyr labelling percentage changed linearly with the increase in the nonapeptide conversion, which suggests that nonapeptide labelling is more selective for tyrosine (Figure 5).

**Figure 6:**
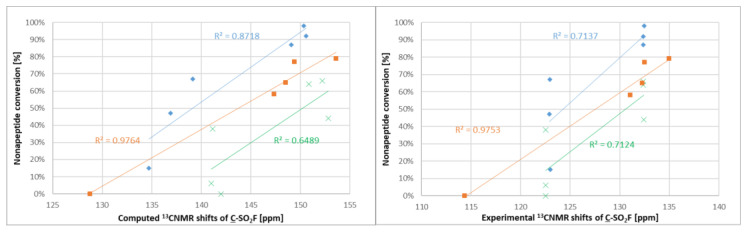
Correlation between calculated (**left**) and experimental (**right**) ^13^C NMR shifts and nonapeptide conversion. Orange: **1**–**5**, blue: **12**–**17**, green: **6**–**11**.

After experimental characterization of the library members, we aimed to find a descriptor that might be useful for reactivity estimation. Therefore, ^19^F NMR shifts were calculated for the sulfonyl fluorides, but there was no correlation observed between the ^19^F NMR shifts and the intrinsic reactivity or the nonapeptide conversion. However, when computing and measuring the ^13^C NMR shifts (Figure S1) of the sulfonyl-attached aromatic carbon and comparing to the nonapeptide conversions, one could see that among the structurally similar compounds **1**–**5** and **12**–**17**, there was a correlation between the NMR shifts and the reactivity; the more reactive compounds had a higher chemical shift (Figure 6). A similar observation was found for compounds **6**–**11**. The methoxyphenylsulfonyl fluoride derivatives (**9**–**11** and **15**–**17**) had systematically lower chemical shifts than the unsubstituted analogues **6**–**8** and **12**–**14**.

**Figure 7:**
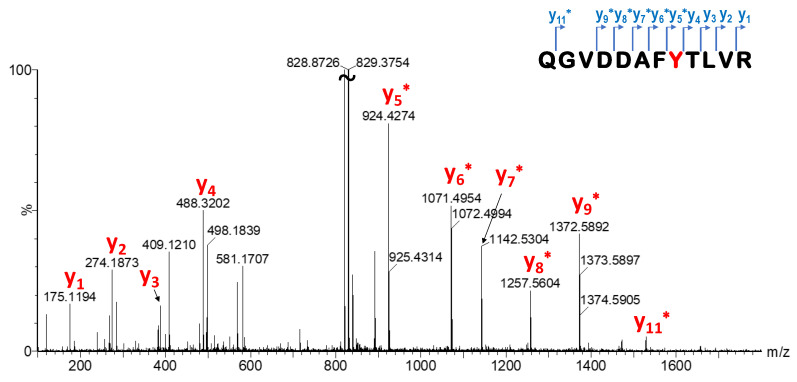
MS/MS spectrum (enlarged) of the KRas^G12D^ 150–161 tryptic peptide modified with covalent probe **12**. Modified fragments are labelled with ∗. Note that only the most intensive sequence fragments are assigned.

Finally, we turned to confirm the biological application of the most potent SuFBit probes. To demonstrate efficient protein labelling, we selected the probes **12** and **14** and applied them to label Kirsten rat sarcoma oncogene mutant (KRas^G12D^). KRas is part of the Ras protein family of membrane-bound GTPases, which act as molecular switches. Somatic KRas mutations are found in several cancers [33]. The labelling efficiency of library members was investigated by MS measurements. After enzymatic digestion, single labelling located on Y157 was confirmed by LC-MS/MS peptide mapping (Figure 7, Supplementary Table S2, Supplementary Figures S3 and S4). This particular tyrosine is located on the edge of the SOS–KRas protein–protein interaction surface area, and there has not been confirmed covalent labelling at this residue until now. However, we should also note that Y157 is not a mutation-dependent residue.

**Figure 8:**
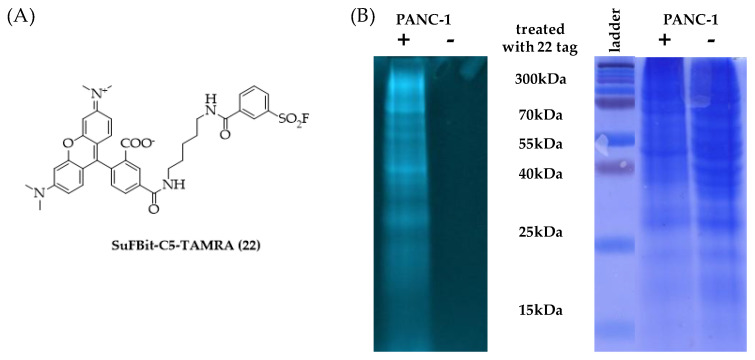
(**A**) SuFBit-C5-TAMRA (**22**) as a fluorescent derivative of probe **12**, and (**B**) SDS-PAGE confirmation of proteome-wide fluorescent labelling with SuFBit-C5-TAMRA (**22**).

Finally, we moved forward to proteome labelling experiments to demonstrate the SuFBit approach. Therefore, we equipped the warhead moiety of probe **12** to a fluorescence reporter backbone and used this specific fluorescent tag to label PANC-1 cell lysate. After we synthesized the SuFBit-C5-TAMRA (**22**) (Figure 8A), we conducted labelling of PANC-1 cell lysate membrane fractions in the presence of 7 mM of probe **22**, applying 2 h of incubation at 37 °C. Next, we applied a general gel electrophoresis protocol to achieve successful confirmation of the lysate labelling by SDS-PAGE analysis (Figure 8B). To identify the exact modification sites, a MS proteomic study was performed. Finally, it was revealed that the SuFBit-derived fluorescent tag (**22**) successfully labelled 18 proteins out of the identified 1529 in the cell lysate. Interestingly, most of the modified peptides were ribosomal proteins; however, some other targets could be identified. Vimentin was also labelled successfully, which was discovered recently to be an attachment factor for SARS-CoV-2 in the process of ACE2-dependent viral entry [34]. Another identified target was the X-ray repair cross-complementing protein 6 (XRCC6), which is generally is required for DNA repair biochemical processes; furthermore, XRCC6 mutations have been described recently as a potential factor in the development of autism [35]. However, we identified a moderate number of labelled proteins and we were able to covalently modify several proteins of PANC-1 cells, which might be valuable starting points for further developments in the field of therapeutics and diagnostics.

The SuFBit methodology and the results we demonstrated herein, identifying the possibly most prevalent targets of these electrophilic fragments to capture proteins in cells (full list of labelled proteins is shown in Supplementary Table S3), might contribute to target validation efforts of early phase oncology programs.

## 3. Materials and Methods

NMR spectra were recorded in DMSO-*d_6_* or CDCl_3_ solution at room temperature on a Varian Unity Inova 500 spectrometer (500 and 125 MHz for ^1^H, ^13^C and APT NMR spectra, respectively), with the residual solvent signal as the lock and TMS as the internal standard, or on a Bruker AVII600 (^1^H = 400 and 600 MHz, ^19^F = 376 MHz) referenced to residual nondeuterated solvent. Chemical shifts (δ) and coupling constants (*J*) are given in ppm and Hz, respectively. HPLC–MS measurements were performed using a Shimadzu LCMS-2020 device equipped with a Reprospher 100 C18 (5 µm; 100 × 3 mm) column and a positive-negative double ion source (DUIS±) with a quadrupole MS analyser in the range of 50–1000 *m*/*z*. The samples were eluted with gradient elution using eluent A (0.1% formic acid in water) and eluent B (0.1% formic acid in acetonitrile). The flow rate was set to 1.5 mL/min. The initial condition was 5% eluent B, followed by a linear gradient to 100% eluent B by 1.5 min, then from 1.5 to 4.0 min, 100% eluent B was retained and from 4.0 to 4.5 min, we reverted back to the initial condition of 5% eluent B which was retained until 5 min. The column temperature was kept at room temperature, and the injection volume was 1–10 µL. HPLC–MS/MS measurements were performed using an ExionLC AC instrument coupled with a Sciex 3500 triple quadrupole mass spectrometer with a Turbo V ion source in electrospray ionization mode. Chromatographic separation was achieved using a Phenomenex 2.6 μm, 100 × 4.6 mm column. Eluents were ultrapure water (A) and acetonitrile (B), both with 0.1% formic acid. The flow rate was 0.5 mL/min. Gradient elution was used, starting at 5% B. The initial ratio was kept for 2 min, then within 8 min, the ratio was raised to 95% B. It was kept there for another 3 min, then dropped back in 0.5 min to 5% and kept there for another 6.5 min for equilibration. The column temperature was kept at room temperature and the injection volume was 10 μL. The MS parameters were as follows: 450 °C source temperature, 5000 V ion spray voltage and 120 V declustering potential. Compressed air was used as the nebulizer gas (GS1) and heater gas (GS2), and nitrogen was used as curtain gas with values set at 35, 45 and 45, respectively. Gasses were provided by a Claind NiGen LCMS gas generator. Compounds **1**–**5** were purchased from Enamine, while **6**–**20** were synthesized. The purity of the compounds was assessed by HPLC with UV detection at 215 and 254 nm; all starting compounds were known, purchased or synthesized and >95% pure. LC-MS grade solvents were purchased from Merck (Darmstadt, Germany). RapiGest SF was obtained from Waters (Milford, MA, USA). Mass spectrometry grade trypsin and trypsin/Lys-C mix digestion enzymes were obtained from Promega (Promega Corporation, Madison, WI, USA). Reagents used for enzymatic digestion (1,4-Dithiothreitol (DTT) and iodoacetamide (IAA)) were purchased from Roche Diagnostics (Roche Diagnostics GmbH, Mannheim, DE) and Fluka Chemie GmbH (Buchs, CH). Other consumables (formic acid—FA, trifluoroacetic acid—TFA, heptafluorobutyric acid—HFBA and NH_4_HCO_3_) were from Merck Life Science Kft. (Merck KGaA., Darmstadt, Germany). 

DFT computations at the M062X/6-311++G(3df,2p) level of theory were performed considering the solvent effect of the corresponding NMR solvent using the CPCM solvent model with the Gaussian 09 and Gaussian 16 program packages. The geometries of the molecules were optimized in all cases, and frequency calculations were also performed to assure that the structures were in a local minimum. The NMR spectra were computed using the GIAO method considering the built-in internal standard, TMS [36,37,38,39,40,41].

***General method for the synthesis of sulfonyl fluorides***

HATU (1-[Bis(dimethylamino)methylene]-1H-1,2,3-triazolo[4,5-b]pyridinium 3-oxid hexafluorophosphate, 0.209 g, 0.550 mmol) was added to a solution of the appropriate acid (0.458 mmol), amine (0.458 mmol, 1 equiv.) and DIPEA (0.160 mL, 0.917 mmol) in *N*,*N*-dimethylformamide (DMF) (4 mL). The reaction mixture was stirred at room temperature followed by LCMS. After 48 h, the mixture was concentrated under reduced pressure. The resulting crude material was partitioned between dichloromethane (10 mL) and water (10 mL) and the organic layers were passed through a phase separator. The solvent was removed by vacuum, and the crude product was purified by preparative HPLC.

*3-(2-Phenylacetamido)benzenesulfonyl fluoride* (**6**)

Yield: 98%, ^1^H NMR (400 MHz, DMSO-*d*6) δ 3.71 (s, 2 H) 7.25–7.42 (m, 6 H) 7.71–7.83 (m, 2 H) 8.52 (t, *J* = 2.0 Hz, 1 H) 10.74 (s, 1 H) ppm; ^19^F NMR (376 MHz, DMSO-*d*6) δ 66.03 (s, 1 F) ppm; ^13^C NMR (126 MHz, DMSO-*d*6) δ 170.55, 141.11, 135.74, 132.40 (d, *J* = 23.4 Hz), 131.60, 129.63, 128.81, 127.16, 126.70, 123.03, 117.95, 43.71 ppm. HRMS (ESI): (M + H)^+^ calcd. for C_14_H_13_NO_3_FS, 294.0600; found 294.0606.

*3-(2-(4-Methoxyphenyl)acetamido)benzenesulfonyl fluoride* (**7**)

Yield: 76%, ^1^H NMR (400 MHz, DMSO-*d*6) δ 3.63 (s, 2 H) 3.74 (s, 3 H) 6.87–6.94 (m, 2 H) 7.24–7.30 (m, 2 H) 7.71–7.83 (m, 3 H) 8.52 (t, *J* = 2.0 Hz, 1 H) 10.68 (s, 1 H) ppm; ^19^F NMR (376 MHz, DMSO-*d*6) δ 66.02 (s, 1 F) ppm; ^13^C NMR (126 MHz, DMSO-*d*6) δ 170.90, 158.62, 141.17, 132.39 (d, *J* = 23.4 Hz), 131.58, 130.64, 127.63, 126.67, 122.96, 117.93, 114.27, 55.51, 42.85 ppm. HRMS (ESI): (M + H)^+^ calcd. for C_15_H_15_NO_4_FS, 324.0705; found 324.0711.

*3-(2-(4-Nitrophenyl)acetamido)benzenesulfonyl fluoride* (**8**)

Yield: 52%, ^1^H NMR (400 MHz, DMSO-*d*6) δ 3.92 (s, 2 H) 7.63 (m, 4 H) 8.22 (m, 3 H) 8.51 (s, 1 H) 10.84 (s, 1 H) ppm; ^19^F NMR (376 MHz, DMSO-*d*6) δ 66.03 (s, 1 F) ppm; ^13^C NMR (126 MHz, DMSO-*d*6) δ 169.45, 146.96, 143.67, 140.90, 132.42 (d, *J* = 23.4 Hz), 131.66, 131.19, 126.79, 123.82, 123.65, 123.21, 118.06, 43.15 ppm. HRMS (ESI): (M + H)^+^ calcd. for C_14_H_12_N_2_O_5_FS, 339.0450; found 339.0467.

*4-Methoxy-3-(2-phenylacetamido)benzenesulfonyl fluoride* (**9**)

Yield: 98%, ^1^H NMR (400 MHz, DMSO-*d*6) δ 3.84 (s, 2 H) 4.03 (s, 3 H) 7.23–7.43 (m, 6 H) 7.84–7.89 (m, 1 H) 8.80–8.83 (m, 1 H) 9.79 (s, 1 H) ppm; ^19^F NMR (376 MHz, DMSO-*d*6) δ ppm 67.75 (s, 1 F) ppm; ^13^C NMR (126 MHz, DMSO-*d*6) δ 170.88, 155.38, 136.07, 129.69, 129.20, 128.78, 127.07, 126.01, 122.55 (d, *J* = 23.4 Hz), 120.03, 112.70, 57.29, 43.17 ppm. HRMS (ESI): (M + H)^+^ calcd. for C_15_H_15_NO_4_FS, 324.0705; found 324.0711.

*4-Methoxy-3-(2-(4-methoxyphenyl)acetamido)benzenesulfonyl fluoride* (**10**)

Yield: 98%, ^1^H NMR (400 MHz, DMSO-*d*6) δ 3.71–3.79 (m, 6 H) 4.02 (s, 2 H) 6.85–6.99 (m, 2 H) 7.19–7.31 (m, 2 H) 7.40 (d, *J* = 8.8 Hz, 1 H) 7.86 (dd, *J* = 8.7, 2.6 Hz, 1 H) 8.80 (d, *J* = 2.5 Hz, 1 H) 9.70 (s, 1 H) ppm; ^19^F NMR (376 MHz, DMSO-*d*6) δ 67.75 (s, 1 F) ppm; ^13^C NMR (126 MHz, DMSO-*d*6) δ 171.22, 158.58, 155.32; 130.70, 129.24, 127.93, 125.94, 122.55 (d, *J* = 24.0 Hz), 119.94, 114.25, 112.67, 57.29, 55.51, 42.31 ppm. HRMS (ESI): (M + H)^+^ calcd. for C_16_H_17_NO_5_FS, 354.0811; found 354.0819.

*4-Methoxy-3-(2-(4-nitrophenyl)acetamido)benzenesulfonyl fluoride* (**11**)

Yield: 20%, ^1^H NMR (400 MHz, DMSO-*d*6) δ 4.00–4.15 (m, 5 H) 7.40–7.45 (m, 1 H) 7.63 (d, *J* = 8.9 Hz, 2 H) 7.86–7.90 (m, 1 H) 8.19–8.24 (d, *J* = 8.9 Hz, 2 H) 8.79 (d, *J* = 2.5 Hz, 1 H) 9.99 (s, 1 H) ppm; ^19^F NMR (376 MHz, DMSO-*d*6) δ 67.76 (s, 1 F) ppm; ^13^C NMR (126 MHz, DMSO-*d*6) δ 169.81, 155.53, 146.89, 144.11, 131.13, 129.01, 126.23, 123.80, 122.55 (d, *J* = 24.0 Hz), 120.35, 112.80, 57.32, 42.74 ppm. HRMS (ESI): (M + H)^+^ calcd. for C_15_H_14_N_2_O_6_FS, 369.0556; found 369.0569.

*3-(Benzylcarbamoyl)benzenesulfonyl fluoride* (**12**)

Yield: 54%, ^1^H NMR (400 MHz, CDCl_3_-*d*) δ 4.70 (d, *J* = 5.9 Hz, 2 H) 6.50 (br s, 1 H) 7.40 (m, 4 H) 7.76 (t, *J* = 7.9 Hz, 1 H) 8.15–8.20 (m, 1 H) 8.26 (d, *J* = 7.9 Hz, 1 H) 8.40 (t, *J* = 1.7 Hz, 1 H) ppm; ^19^F NMR (376 MHz, CDCl_3_) δ 66.14 (s, 1 F) ppm; ^13^C NMR (126 MHz, DMSO-*d*6) δ 164.22, 139.46, 136.48, 135.74, 132.39 (d, *J* = 24.1 Hz), 131.34, 131.29, 128.80, 127.87, 127.39, 127.29, 43.43 ppm. HRMS (ESI): (M + H)^+^ calcd. for C_14_H_13_NO_3_FS, 294.0600; found 294.0606.

*3-((4-Methoxybenzyl)carbamoyl)benzenesulfonyl fluoride* (**13**)

Yield: 70%, ^1^H NMR (400 MHz, DMSO-*d*6) δ 3.74 (s, 3 H) 4.45 (d, *J* = 5.4 Hz, 2 H) 6.91 (d, *J* = 8.9 Hz, 2 H) 7.28 (d, *J* = 8.9 Hz, 2 H) 7.89–7.95 (m, 1 H) 8.31 (d, *J* = 8.4 Hz, 1 H) 8.43 (d, *J* = 7.9 Hz, 1 H) 8.58 (s, 1 H) 9.38 (br t, *J* = 5.9 Hz, 1 H) ppm; ^19^F NMR (376 MHz, DMSO-*d*6) δ 66.37 (s, 1 F); ^13^C NMR (126 MHz, DMSO-*d*6) δ 164.07, 158.81, 136.56, 135.72, 132.36 (d, *J* = 24.1 Hz), 131.41, 131.31, 131.24, 129.29, 127.25, 114.23, 55.54, 42.90 ppm. HRMS (ESI): (M + H)^+^ calcd. for C_15_H_15_NO_4_FS, 324.0705; found 324.0713.

*3-((4-Nitrobenzyl)carbamoyl)benzenesulfonyl fluoride* (**14**)

Yield: 34%, ^1^H NMR (400 MHz, CDCl_3_) δ ppm 4.79 (d, *J* = 5.9 Hz, 2 H) 6.93 (br s, 1 H) 7.52–7.58 (m, 2 H) 7.79 (t, *J* = 7.9 Hz, 1 H) 8.06–8.27 (m, 4 H) 8.29 (d, *J* = 7.9 Hz, 1 H) ppm; ^19^F NMR (376 MHz, CDCl_3_) δ 66.17 (s, 1 F) ppm; ^13^C NMR (126 MHz, DMSO-*d*6) δ 164.50, 147.52, 147.03, 136.16, 135.77, 132.45 (d, *J* = 24.1 Hz), 131.48, 131.42, 128.86, 127.32, 123.99, 43.00 ppm. HRMS (ESI): (M + H)^+^ calcd. for C_14_H_12_N_2_O_5_FS, 339.0450; found 339.0461.

*3-(Benzylcarbamoyl)-4-methoxybenzenesulfonyl fluoride* (**15**)

Yield: 91%, ^1^H NMR (400 MHz, DMSO-*d*6) δ 4.04 (s, 3 H) 4.52 (d, *J* = 6.4 Hz, 2 H) 7.26 (d, *J* = 3.9 Hz, 1 H) 7.35 (d, *J* = 4.4 Hz, 4 H) 7.52 (d, *J* = 8.9 Hz, 1 H) 8.22–8.29 (m, 2 H) 8.87–8.96 (m, 1H) ppm; ^19^F NMR (376 MHz, DMSO-*d*6) δ 67.83 (s, 1 F) ppm; ^13^C NMR (126 MHz, DMSO-*d*6) δ 163.55, 163.15, 139.57, 133.17, 130.93, 128.75, 127.58, 127.24, 125.88, 122.92 (d, *J* = 24.4 Hz), 114.53, 57.56, 43.24 ppm. HRMS (ESI): (M + H)^+^ calcd. for C_15_H_15_NO_4_FS, 324.0705; found 324.0714.

*4-Methoxy-3-((4-methoxybenzyl)carbamoyl)benzenesulfonyl fluoride* (**16**)

Yield: 56%, ^1^H NMR (400 MHz, DMSO-*d*6) δ 3.74 (s, 3 H) 4.03 (s, 3 H) 4.44 (d, *J* = 5.9 Hz, 2 H) 6.88–6.95 (m, 2 H) 7.25–7.31 (m, 2 H) 7.50 (d, *J* = 8.4 Hz, 1 H) 8.21–8.27 (m, 2 H) 8.80–8.88 (m, 1 H) ppm; ^19^F NMR (376 MHz, DMSO-*d*6) δ 67.83 (s, 1 F) ppm; ^13^C NMR (126 MHz, DMSO-*d*6) δ 163.41, 163.12, 158.70, 133.12, 131.50, 130.88, 128.97, 125.97, 122.90 (d, *J* = 24.6 Hz), 114.50, 114.19, 57.55, 55.53, 42.70 ppm. HRMS (ESI): (M + H)^+^ calcd. for C_16_H_17_NO_5_FS, 354.0811; found 354.0820.

*4-Nitro-3-((4-methoxybenzyl)carbamoyl)benzenesulfonyl fluoride* (**17**)

Yield: 28%, ^1^H NMR (400 MHz, DMSO-*d*6) δ 4.07 (s, 3 H) 4.64 (d, *J* = 5.9 Hz, 2 H) 7.54 (d, *J* = 8.9 Hz, 1 H) 7.62 (d, *J* = 8.9 Hz, 2 H) 8.23 (d, *J* = 8.9 Hz, 4 H) 9.09 (br t, *J* = 5.9 Hz, 1 H) ppm; ^19^F NMR (376 MHz, DMSO-*d*6) δ 67.81 (s, 1 F) ppm; ^13^C NMR (126 MHz, DMSO-*d*6) δ 163.81, 163.20, 147.75, 146.93, 133.35, 131.03, 128.60, 125.48, 123.95, 123.00 (d, *J* = 24.6 Hz), 114.59, 57.63, 42.95 ppm. HRMS (ESI): (M + H)^+^ calcd. for C_15_H_14_N_2_O_6_FS, 369.0556; found 369.0566.

*4-(2-(Benzylamino)-2-oxoethyl)benzenesulfonyl fluoride* (**18**)

Yield: 41%, ^1^H NMR (400 MHz, CDCl_3_) δ 3.70 (s, 2 H) 4.47 (d, *J* = 5.4 Hz, 2 H) 5.85 (br s, 1 H) 7.24–7.38 (m, 5 H) 7.58 (d, *J* = 8.4 Hz, 2 H) 7.95–8.04 (m, 2 H) ppm; ^19^F NMR (376 MHz, CDCl_3_) δ 66.10 (1 F) ppm; ^13^C NMR (101 MHz, CDCl_3_) 43.32, 44.05, 127.82, 128.84, 130.04 (d, *J* = 24.6 Hz), 130.55, 131.80, 132.04, 137.65, 143.33, 168.58 ppm. HRMS (ESI): (M + H)^+^ calcd. for C_15_H_15_NO_3_FS, 308.0756; found 308.0766.

*4-(2-((4-Methoxybenzyl)amino)-2-oxoethyl)benzenesulfonyl fluoride* (**19**)

Yield: 22%, ^1^H NMR (400 MHz, DMSO-*d*6) δ 2.51 (t, *J* = 2.0 Hz, 2 H) 3.73 (s, 3 H) 4.22 (d, *J* = 5.4 Hz, 2 H) 6.85–6.91 (m, 2 H) 7.14–7.22 (m, 2 H) 7.68 (d, *J* = 8.4 Hz, 2 H) 8.06–8.12 (m, 2 H) 8.60 (br t, *J* = 5.7 Hz, 1 H) ppm; ^19^F NMR (376 MHz, DMSO-*d*6) δ 66.62 (1 F) ppm; ^13^C NMR (101 MHz, DMSO-*d*6) δ 42.32, 42.52, 55.55, 114.20, 128.79, 129.12, 130.01 (d, *J* = 23.4 Hz), 131.50, 131.53, 146.46, 158.75, 169.05 ppm. HRMS (ESI): (M + H)^+^ calcd. for C_16_H_17_NO_4_FS, 338.0862; found 338.0873.

*4-(2-((4-Nitrobenzyl)amino)-2-oxoethyl)benzenesulfonyl fluoride* (**20**)

Yield: 28%, ^1^H NMR (400 MHz, CDCl_3_) δ 3.76 (s, 2 H) 4.57 (d, *J* = 5.9 Hz, 2 H) 5.95 (br s, 1 H) 7.36–7.51 (m, 2 H) 7.60 (d, *J* = 7.9 Hz, 2 H) 7.96 -8.09 (m, 2 H) 8.13–8.28 (m, 2 H) ppm; ^19^F NMR (376 MHz, CDCl_3_) δ 66.05 (1 F) ppm; ^13^C NMR (125 MHz, DMSO-*d*6) 169.58, 147.81, 146.95, 146.12, 131.56, 130.11 (d, *J* = 24.6 Hz), 128.83, 128.67, 123.94, 42.38, 42.37 ppm. HRMS (ESI): (M + H)^+^ calcd. for C_15_H_14_N_2_O_5_FS, 353.0607; found 353.0620.

*2-(6-(Dimethylamino)-3-(dimethyliminio)-3H-xanthen-9-yl)-5-((5-(3-(fluorosulfonyl)benzamido)pentyl)carbamoyl)benzoate* (**22**)

^1^H NMR (500 MHz, DMSO-*d*6) δ 8.83 (s, 1H), 8.73–8.66 (m, 2H) 8.54 (t, *J* = 5.6 Hz, 2H), 8.27 (d, *J* = 7.9 Hz, 1H), 8.11 (d, *J* = 1.8 Hz, 1H), 7.77 (dt, *J* = 7.9, 1.4 Hz, 2H), 7.71 (dt, *J* = 7.8, 1.5 Hz, 1H), 7.37 (t, *J* = 7.7 Hz, 2H), 7.03 (broaden s, 2H), 3.06 (s, 6H), 2.64 (t, *J* = 2.0 Hz, 2H), 2.37 (t, *J* = 1.5, 2H), 1.66–1.57 (m, 4H), 1.44–1.38 (m, 2H), 1.25 (s, 6H) ppm. ^13^C NMR (125 MHz, DMSO-*d*6) 171.0; 167.5; 154.6; 150.8; 142.4; 139.8; 135.3; 134.9; 134.1; 134.0; 133.2; 132.9; 130.9; 130.1; 128.9; 128.5; 127.2; 123.3; 113.9; 105.1; 100.2; 96.7; 54.5; 42.4; 17.3; 15.9; 11.7 ppm. HRMS (ESI): (M + H)^+^ calcd. for C_37_H_38_N_4_O_7_SF^+^, 701.2439; found, 701.2432.

***Intrinsic reactivity assay***

In an LC-MS vial, 800 uL PBS (pH 7.4) buffer, 188 uL DMSO, 6 uL 100 mM 1,4-dicyanobenzene as internal standard in DMSO and 6 uL 100 mM sulfonyl fluoride in DMSO was mixed. The final concentration of the sulfonyl fluoride was 600 uM. The final mixture was analysed by HPLC-MS after 0, 1, 2, 3, 6, 12 and 24 h time intervals. The AUC (area under the curve) values were determined via integration of HPLC spectra, then corrected with internal standard. The fragment AUC values were applied for ordinary least squares (OLS) linear regression and for computing the important parameters (kinetic rate constant and half-life time), a programmed excel (Visual Basic for Applications) was utilized. The data are expressed as means of duplicate determinations, and the standard deviations were within 15% of the given values.

***Nonapeptide reactivity and promiscuity assay***

For the nonapeptide assay, two initial solutions were prepared: a 1 mM solution of the fragment in dimethyl sulfoxide (DMSO) (A) and a 100 μM solution of the nonapeptide in PBS buffer (pH 7.4) (B). The reaction mixture contained 10 μL A, 50 μL B and 40 μL PBS. The samples were incubated at room temperature for 16 hours. An information-dependent acquisition (IDA) LC-MS/MS experiment was used to identify the number of bindings and their sites. MS scanning was applied as a survey scan and product ion was the dependent scan. The collision energy in the product ion scan was set to 30 eV. The reference data for identification were provided by the GPMAW 12.20 software. The relative quantitation of the nonapeptide and its fragment covalent conjugates was calculated from the total ion chromatograms. For every component, three corresponding ions were selected and their sums of area under the curve were used.

***Chromogenic intrinsic reactivity assay with DPF sensor***

Stock solutions of the DPF sensor and the compounds were prepared in DMSO. In the assay, *N*-acetyl-tyrosine (1 mM) and DBU (1 mM) were incubated for 10 min at 25 °C. The SuFex compounds (50 μM) were added to the plate, followed by the DPF sensor (50 μM). After 15 minutes incubation at room temperature, the release of 7-hydroxy-8-formylcoumarin was monitored by measuring the fluorescence endpoint at 𝜆_ex_ = 360 nm and 𝜆_em_ = 445 nm. The fluorescent reactivity assay was measured by a SpectraMax iD5 Multi-Mode Microplate Reader (Molecular Devices, San Jose, CA, USA).

***LC-MS/MS peptide mapping analysis***

Mass spectrometric experiments were performed on a high-resolution hybrid quadrupole-time-of-flight mass spectrometer (Waters Select Series Cyclic IMS, Waters Corp., Wilmslow, U.K.). The mass spectrometer operated in positive V mode. Leucine enkephalin was used as the Lock Mass standard. Chromatographic separations were performed on a Waters Acquity I-Class UPLC system coupled directly to the mass spectrometer. Modification sites on the KRas^G12D^ protein were determined by RPLC-MS/MS peptide mapping after proteolysis using trypsin. Briefly, proteins were enzymatically digested after buffer exchange using Amicon Ultra-0.5 mL Centrifugal Filter units (10 KDa, Merck Millipore). Protein samples were reduced by dithiothreitol at 37 °C for 30 min. Tryptic cleavage was performed in 50 mM NH_4_HCO_3_ solution. A trypsin–LysC mixture (Promega Corporation, Madison, WI, USA) was used for enzymatic digestion. Proteins were digested for 4 h using a 1:50 enzyme:protein ratio at 37 °C. Digestion was stopped by adding formic acid in a final concentration of 0.2% (V/V). Gradient elution was performed on a Waters Acquity CSH Peptide C18 UPLC column (2.1 × 150 mm, 1.7 µm) under the following parameters: mobile phase “A”: 0.1% formic acid in water, mobile phase “B”: 0.1% formic acid in acetonitrile; flow rate: 300 µL/min; column temperature: 60 °C; gradient: 2 min: 2% B, 80 min: 45% B, 81 min: 85% B. HDMS^E^ experiments were performed using collision voltage ramping in the transfer cell under the following parameters: *m*/*z* 50–2000; scan time: 0.3 sec; single Lock Mass: leucine enkephalin; low energy: 6 V; high energy: ramping 19–45 V. The cyclic ion mobility cell was operated in single pass mode. BiopharmaLynx 1.3.5 software (Waters Corp., Wilmslow, U.K.) was used for data analysis.

***Production of PANC-1 cell lysates***

Cells were grown in RPMI 1640 medium at 37 °C in a humidified atmosphere with 5% CO_2_, as described. When the confluency of pancreatic cancer cells, PANC-1, bearing a KRas^G12D^ mutation reached 70%, the growth medium was discarded and cells were washed twice with phosphate-buffered saline (PBS; Biosera), harvested in 5 mL PBS (Biosera) using a cell scraper (Sarstedt) and collected in a 15 mL conical tube (Sarstedt). Then, cells were centrifuged for 5 min at 1000× *g* at 4 °C (Eppendorf 5804R centrifuge, Eppendorf AG, Hamburg, Germany), the supernatant was removed, and to the pellet was added a protease and phosphatase inhibitor cocktail mix (Thermo Scientific, Rockford, IL, USA). The cell pellet was resuspended in a 4× volume of PBS (Biosera) on ice, transferred into a 1.5ml tube (Corning, Reynosa, Mexico) and homogenized using a probe sonicator (model Q55, QSonica, Newtown, CT, USA), 10× for 1 sec at 20% power, on ice. After this, centrifugation was applied for 5 min at 12,000× *g* at 4 °C (Heraeus Biofuge Fresca centrifuge, Kendro Laboratory Products, Osterode, Germany) to pellet nuclei and unbroken cells. The supernatant was transferred to an ultracentrifuge sealing tube (OptiSeal, Beckman Coulter, Brea, CA, United States) and centrifuged for 1 h at 100,000× *g* at 4 °C (Optima MAX-XP Ultracentrifuge, Beckman Coulter) to separate the membrane (pellet) and soluble (supernatant) protein fractions. The supernatant fraction was transferred to a clean 1.5ml tube (Corning), while the pellet was washed with cold PBS and resuspend in cold PBS (Biosera). The protein concentrations in the membrane and the soluble fractions were quantified by a Pierce BCA protein assay kit (Thermo Scientific) according to the manufacturer’s protocol, and the protein concentration was adjusted to the desired concentration (1–2 mg/mL) with cold PBS (Biosera). Samples were aliquoted in a volume of 50 µL and stored at −80 °C. Prior to ABPP probe labelling, samples were thawed on ice and briefly allowed to warm up to room temperature.

***Fluorogenic labelling of PANC-1 cell lysates***

Membrane fractions of pancreatic cancer cells (PANC-1) lysates, bearing the KRas^G12D^ mutation, were applied for labelling with the SuFBit-C5-TAMRA (**22**) fluorescent tag as follows. An amount of 75 µL of 1 mg/mL lysate solutions in PBS buffer were treated with 5.6 µL of 100 mM SF-C5-TAMRA (**22**) in DMSO. The reaction mixture was incubated at 37 °C for 2 h. Next, 25 µL was submitted for SDS-PAGE analysis and 50 µL of the resulting mixture was submitted for MS analysis.

***Cell lysate analysis by SDS-PAGE***

Polyacrylamide gel electrophoresis was performed with an mPAGE^®^TurboMix Bis-Tris Gel Casting Kit (TMKIT, Merck & Co., Rahway, NJ, USA), following standard lab procedures. An 8% resolving gel and a 4% stacking gel were used and a broad-range MW marker (4.6–300 kDa, ProSieve QuadColor Protein Marker, Lonza) was co-run to estimate protein weights. Samples were mixed with the loading buffer in a 3:1 ratio (composition for 6 × SDS: 1 g SDS, 3 mL glycerol, 6 mL 0.5 M Tris buffer pH = 6.8 and 2 mg Coomassie blue R250 in 10 mL) and heated at 65 °C for 5 minutes. Samples were subsequently loaded into the wells. All gels were run at a constant 200 mA for 60 minutes. Fluorescent gel images were recorded with a Bio-Rad Gel Doc XR+ (Bio-Rad Laboratories, Hercules, CA, USA), applying 𝜆_ex_ = 542 nm and 𝜆_em_ = 568 nm. Gels were then stained using a Coomassie stain (0.12 g Coomassie blue G-250, 0.10 g Coomassie blue R-250, 500 mL MeOH, 400 mL distilled water and 100 mL acetic acid), and after washing, it was rested at room temperature for 16 h in a water–ethanol mixture.

***Cell lysate analysis by mass spectrometry***

The sample solvent was exchanged to 50 mM NH_4_HCO_3_ solution using a Millipore Amicon Ultra 10 kDa centrifugation filter. The filters were rinsed with LC-MS water for 10 min (13,500 rpm at 4 °C). Then, the samples were added and their volume was made up to 200 μL with 200 mM NH_4_HCO_3_ solution and centrifuged for 10 min (13,500 rpm at 4 °C). Two additional cycles (10 min, 13,500 rpm, 4 °C) were performed, the first one by adding 200 μL 200 mM NH_4_HCO_3_ solution and the second one using 200 μL 50 mM NH_4_HCO_3_ solution. The samples were digested in solution using trypsin, as previously described with minor modifications [42]. In brief, portions of each sample, each containing approximately 10 µg of protein, were diluted with water and methanol to a final volume of 25 µL, with a final methanol concentration of 5 %. Denaturation of the proteins was performed by adding 0.5% RapiGest SF (5 µL). For S-S bridge reduction, DTT was added at a final concentration of 12.5 mM and incubated at 60 °C for 30 min. For alkylation, IAA was added at a final concentration of 12.5 mM and incubated in the dark at room temperature for 30 min. Enzymatic digestion was performed with the addition of 2 µL of 0.25 µg/µL trypsin/Lys-C mix, with an incubation time of 1 hour at 37 °C, followed by the addition of 2 µL of 1 µg/µL trypsin, incubated for 2 h at 37 °C. The reaction was quenched by the addition of 1.5 μL formic acid and the sample was dried in a SpeedVac at 50 °C and dissolved in 50 µL 0.1% TFA prior to cleaning. Peptide clean-up and desalting were performed on Pierce C18 spin columns (Thermo Fisher Scientific, Waltham, MA, USA). Briefly, column conditioning and equilibration was performed using 2 × 200 µL 50% MeOH and 2 × 200 µL 0.5% TFA and 5% acetonitrile (AcN), respectively. The column was washed with 2 × 200 µL 0.1% HFBA. Then, the sample, reconstituted in 50 µL 0.1% HFBA, was applied and the flowthrough was reapplied to the column. HFBA (0.1%, 2 × 100 µL) was used for sample washing. Finally, sample elution was performed using 2 × 50 µL 0.1% TFA and 70% AcN and 50 µL 0.1% FA and 70% AcN. In each step, centrifugation was carried out for 2 min at 2000 rpm. Then, the sample was dried in a SpeedVac at 50 °C.

Mass spectrometry measurements were performed on a Maxis II ETD Q-TOF (Bruker Daltonics, Bremen, Germany) equipped with a CaptiveSpray nanoBooster ion source coupled to an Ultimate 3000 nanoRSLC system (Dionex, Sunnyvale, CA, USA). Samples were dissolved in 2% can and 0.1% FA, and in each run, 2 µg protein was injected onto an Acclaim PepMap100 C-18 trap column (100 Å, 5 µm, 100 µm × 20 mm, Thermo Fisher Scientific, Sunnyvale, CA, USA) for sample desalting. Peptides were separated on an ACQUITY UPLC M-Class Peptide BEH C18 column (130 Å, 1.7 µm, 75 µm × 250 mm, Waters, Milford, MA, USA) at 48 °C, applying gradient elution (2% B from 0 to 11 min, followed by a 120 min gradient to 50% B). Eluent A consisted of water + 0.1% formic acid, while eluent B was acetonitrile + 0.1% formic acid. MS spectra were recorded over a mass range of 150–2200 *m*/*z* at 3 Hz, while the CID was performed at 16 Hz for abundant precursor ions and at 4 Hz for low abundance ones. Sodium formate was used as an internal standard and raw data were recalibrated by the Compass Data Analysis software 4.3 (Bruker Daltonik GmbH, Bremen, Germany).

Proteins were identified by searching against the SwissProt Human (downloaded: 12/08/2022) database with addition of a mutated sequence of the RASK_HUMAN (P01116) protein (G12 → D12) using the Byonic (v3.5.0, Protein Metrics Inc., Cupertino, CA, USA) software search engine. The LC-MS/MS results were searched in Byonic with the following parameters: 4 ppm peptide mass tolerance, 20 ppm fragment mass tolerance, 2 missed cleavages, trypsin as the enzyme, carbamidomethylation of cysteines as the fixed modification, ammonia loss (N-term C), oxidation (M), acetyl (Protein N-term), Glu->Pyro-Glu (N-term E), Gln->Pyro-Glu (N-term Q) and carbamidomethylation (C) as the variable modification. SuFBit-C5-TAMRA (**22**) was added as a custom modification of +680.230473 Da mass shift, at the following possible sites: N-term, K, R, Q, S, T, Y. The requirements for protein identification were set in Scaffold 4.10 (Proteome Software, USA) with the following parameters: LFDR-rescoring on 95% peptide confidence threshold, minimum of two unique peptides and a protein-level False Discovery Rate of 1%.

## 4. Conclusions

We present the systematic characterization of a sulfonyl fluoride library in order to determine the ideal choice of sulfonyl fluoride warhead for the development of larger libraries. The intrinsic reactivity and promiscuity of the sulfonyl fluorides were measured in LC-MS- or LC-MS/MS-based assays that are open for future applications. We also developed and performed a plate-based chromogenic assay with DPF-based fluoride detection, where the same probes were identified as most active. We observed a correlation between computed and measured ^13^C NMR data and nonapeptide conversion of the sulfonyl fluorides that could also be a readily available platform for quick reactivity estimation. We have shown that sulfonyl fluorides are usually not selective, but still show a high preference towards tyrosine. After in-depth characterization at the surrogate level, we successfully applied SuFBits in labelling of the KRas^G12D^ oncogene mutant, and we also confirmed the functional consequences of the covalent binding. Moreover, we could apply the SuFBit methodology to explore the possible targets responsible for the antiproliferative effect of the most potent covalent probes by equipping the warhead moiety to a fluorescent reporter. We also identified possible covalent targets in the PANC-1 cancer cell lysate. Based on our dataset, we present a carboxylic acid and an amino-substituted sulfonyl fluoride warhead that could be applied in library design for constant or flexible reactivity, depending on the non-covalent part of the skeleton.

## Figures and Tables

**Table 1:** Kinetic rate constants (k) and half-lives (t_1/2_) resulting from the intrinsic reactivity assay.

Compound	k (^1^/_h_)	t_1/2_ (h)
**1**	0.0482	14.4
**2**	0.0166	41.9
**3**	0.0074	94.3
**4**	0.0215	32.2
**5**	0.0320	21.7
**6**	0.0543	12.8
**7**	0.0595	11.6
**8**	0.0648	10.7
**9**	0.0695	10.0
**10**	0.0646	10.7
**11**	0.0519	13.4
**12**	0.1381	5.0
**13**	0.1639	4.2
**14**	0.1640	4.2
**15**	0.0185	37.5
**16**	0.0615	11.3
**17**	0.1258	5.5
**18**	0.7280	1.0
**19**	0.4194	1.7
**20**	0.1598	4.3

## Data Availability

The authors declare that the data supporting the findings of this study are available within the paper and its Supplementary Information files. Should any raw data files be needed in another format they are available from the corresponding author upon reasonable request.

## Supplementary Materials

The following supporting information can be downloaded at: https://www.mdpi.com/article/10.3390/molecules28073042/s1, Table S1: Characterization of the sulfonyl fluoride library; Figure S1: Correlation between calculated and measured ^13^C-NMR shifts; Figure S2: MS/MS spectrum of nonapeptide assay; Table S2 Peptide mapping analysis of KRas^G12D^ labelled with **12** and **14** probes; Figure S3: Mass spectrum of KRas^G12D^ 150–161 tryptic peptide modified with probe **12** and **14**; Figure S4: Mass spectrum of the KRas^G12D^ 150–161 tryptic peptide modified with probe **12**; Table S3: Results of PANC-1 lysate labelling experiments analysed by mass spectrometry; Supplementary Methods: Synthesis of DPF sensor molecule; Supplementary References [24,31,43,44,45,46,47].

## Sample Availability

Samples of the compounds **1**–**20** are available from the authors.
