# Supplementary material for: Activation-Free Sulfonyl Fluoride Probes for Fragment Screening
Source: Molecules. 2023 Mar 29;28(7):3042. doi: 10.3390/molecules28073042 (PMC10096327; doi:10.3390/molecules28073042)
Supplement: Supplementary file 1 [file molecules-28-03042-s001.zip › molecules-2313385-supplementary.pdf]

Supplementary Information

Activation-Free Sulfonyl Fluoride Probes for Fragment Screening

László Petri [1,2,†], Péter Ábrányi-Balogh [1,2,3,†], Noémi Csorba [1,2,3], Aaron Keeley [1], József Simon [1,4], Ivan Ranđelović [5], József Tóvári [6], Gitta Schlosser [7], Dániel Szabó [8], László Drahos [8] and György M. Keserű [1,2,3,*]

[1] Medicinal Chemistry Research Group, Research Centre for Natural Sciences, Magyar Tudósok Krt. 2, 1117 Budapest, Hungary; petri.laszlo@ttk.hu (L.P.); abranyi-balogh.peter@ttk.hu (P.Á.-B.); csorba.noemi@ttk.hu (N.C.); aaron.keeley1989@gmail.com (A.K.); simon.jozsef@ttk.hu (J.S.)
[2] National Laboratory for Drug Research and Development, Research Centre for Natural Sciences, Magyar Tudósok Krt. 2, 1117 Budapest, Hungary
[3] Department of Organic Chemistry and Technology, Budapest University of Technology and Economics, Szent Gellért tér 4, 1111 Budapest, Hungary
[4] Research Centre for Natural Sciences, MS Metabolomics Research Group, Magyar Tudósok Krt. 2, 1117 Budapest, Hungary
[5] KINETO Lab Ltd., Zápor u. 55, 1032 Budapest, Hungary; ivan.randelovic@kinetolab.hu
[6] Department of Experimental Pharmacology and National Tumor Biology Laboratory POB 21, National Institute of Oncology, 1525 Budapest, Hungary; tovari.jozsef@oncol.hu
[7] MTA-ELTE Lendület Ion Mobility Mass Spectrometry Research Group, Institute of Chemistry, ELTE Eötvös Loránd University, Pázmány Péter Sétány 1/A, 1117 Budapest, Hungary; gitta.schlosser@ttk.elte.hu
[8] MS Proteomics Research Group, Research Centre for Natural Sciences, Magyar Tudósok Krt. 2, 1117 Budapest, Hungary; szabo.daniel@ttk.hu (D.S.); drahos.laszlo@ttk.hu (L.D.)
* Correspondence: keseru.gyorgy@ttk.hu
† These authors contributed equally to this work.

Table of Contents

Table S1. Characterization of the sulfonyl fluoride library 2

Figure S1. Correlation between calculated and measured ^{13}C NMR shifts 2

Figure S2. MS/MS spectrum of nonapeptide assay 3

Table S2. Peptide mapping analysis for KRas^{G12D} labelled with **12** and **14** probes 24

Figure S3. Mass spectrum of KRas^{G12D} 150-161 tryptic peptide modified with probe **12** and **14** 25

Figure S4. Mass spectrum of KRasG12D 150-161 tryptic peptide modified with probe **14** 25

Table S3. Results of PANC-1 lysate labelling experiments analysed by mass spectrometry 26

Supplementary Methods 26

Supplementary References 28

Table S1. Characterization of the sulfonyl fluoride library

ID	^{13}C NMR measured [ppm]	^{13}C NMR calculated [ppm]	Nonapeptide labelling conversion [%]	TYROSINE labelling conversion [%]	LYSINE labelling conversion [%]	Measured Fluorescence Intensity in DPF-assay [-]
**1**	131.09	147.34	83%	79%	4%	6465428
**2**	132.54	149.40	84%	70%	14%	12979003
**3**	114.38	128.80	0%	0%	0%	6762316
**4**	132.3 [24]	153.64	65%	65%	0%	4682278
**5**	135.0 [24]	148.55	79%	79%	0%	4788817
**6**	132.40	150.87	59%	58%	1%	11413202
**7**	132.39	152.21	67%	64%	3%	14500736
**8**	132.43	152.82	47%	46%	1%	9887691
**9**	122.55	140.99	5%	5%	0%	8446817
**10**	122.55	141.16	7%	7%	0%	7763602
**11**	122.55	141.97	0%	0%	0%	7083526
**12**	132.40	150.57	94%	72%	22%	19124365
**13**	132.37	149.09	83%	79%	4%	15615169
**14**	132.46	150.33	96%	59%	36%	20431421
**15**	122.93	139.16	70%	67%	2%	5602622
**16**	122.90	136.92	52%	49%	3%	7351058
**17**	123.01	134.73	16%	16%	0%	8187924
**18**	130.04	145.42	48%	47%	1%	8761611
**19**	130.01	149.21	25%	25%	0%	8650134
**20**	130.10	149.99	46%	41%	5%	8115745

Figure S1. Correlation between calculated and measured ^{13}C NMR shifts

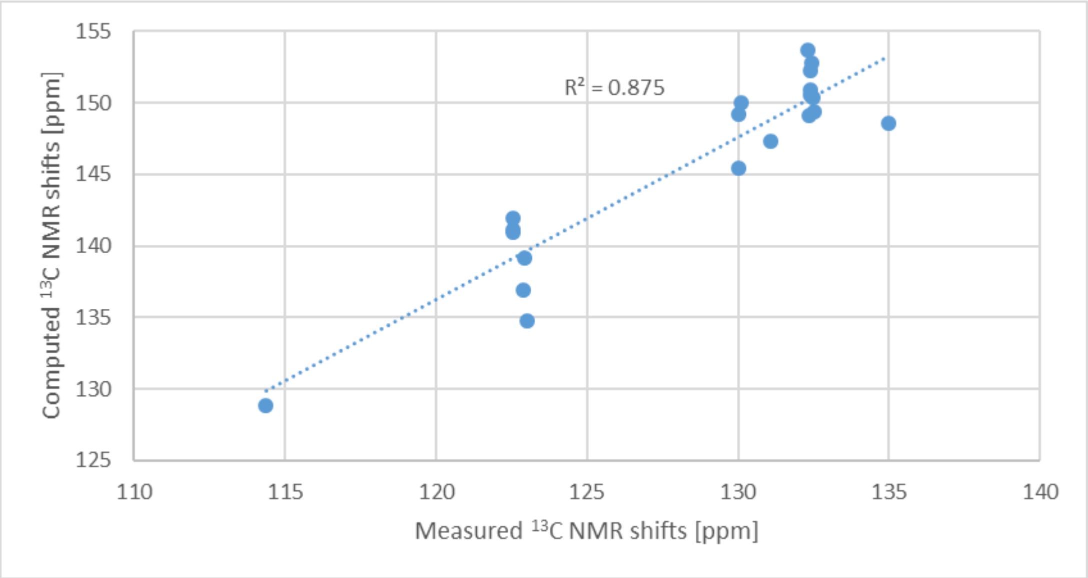

Figure S2. MS/MS spectrum of nonapeptide assay

Representative TIC is presented for compound **12**.

Nonapeptide

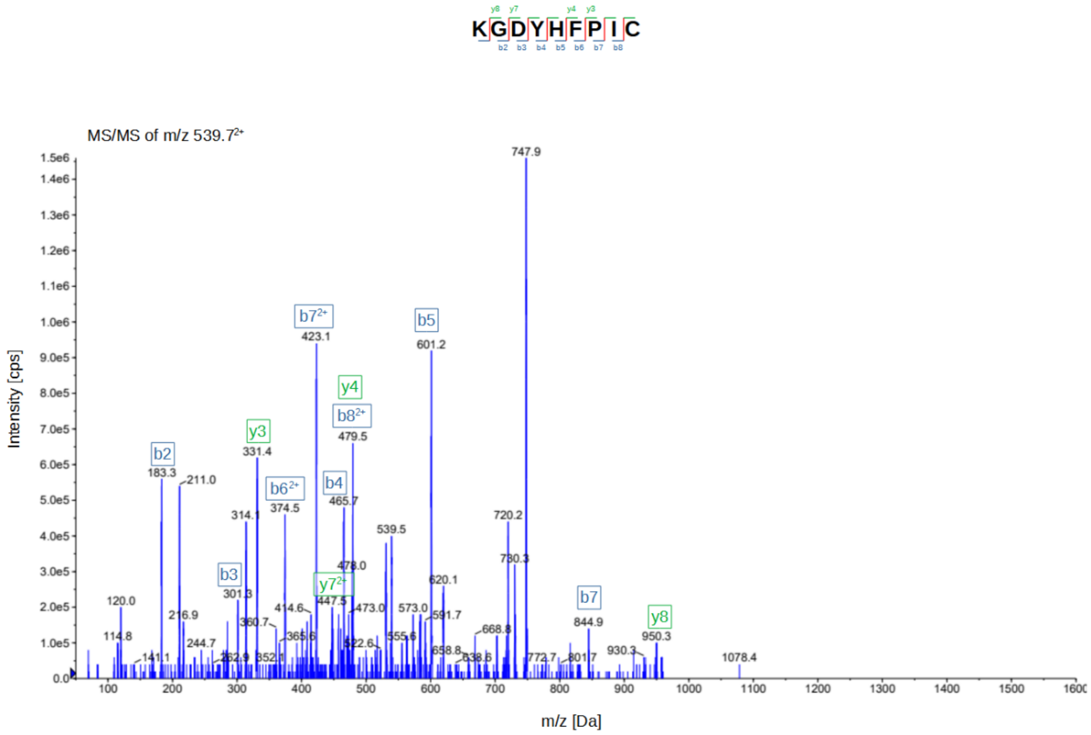

Nonapeptide dimer

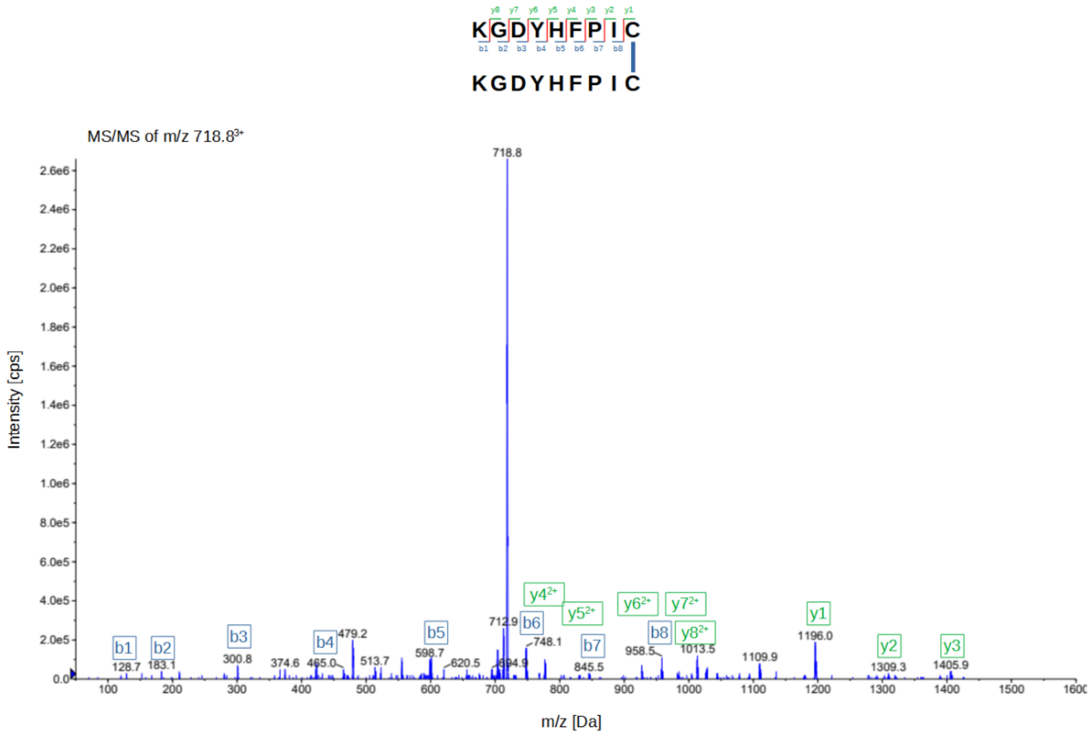

NP+1

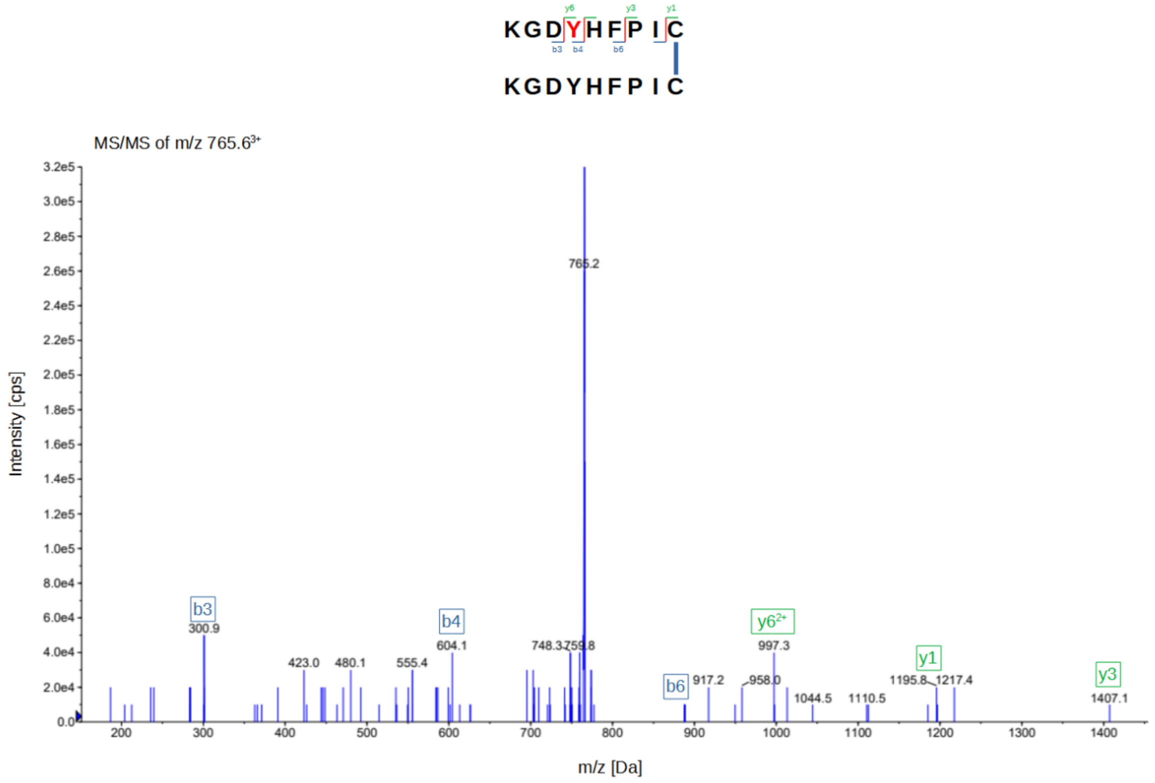

KGDYHFPIC
KGDYHFPIC
MS/MS of m/z 765.6³⁺
Intensity [cps]
m/z [Da]
b3
300.9
b4
604.1
b6
y6²⁺
997.3
y1
y3
1407.1
765.2
423.0
480.1
555.4
748.3
759.8
917.2
958.0
1044.5
1110.5
1195.8
1217.4

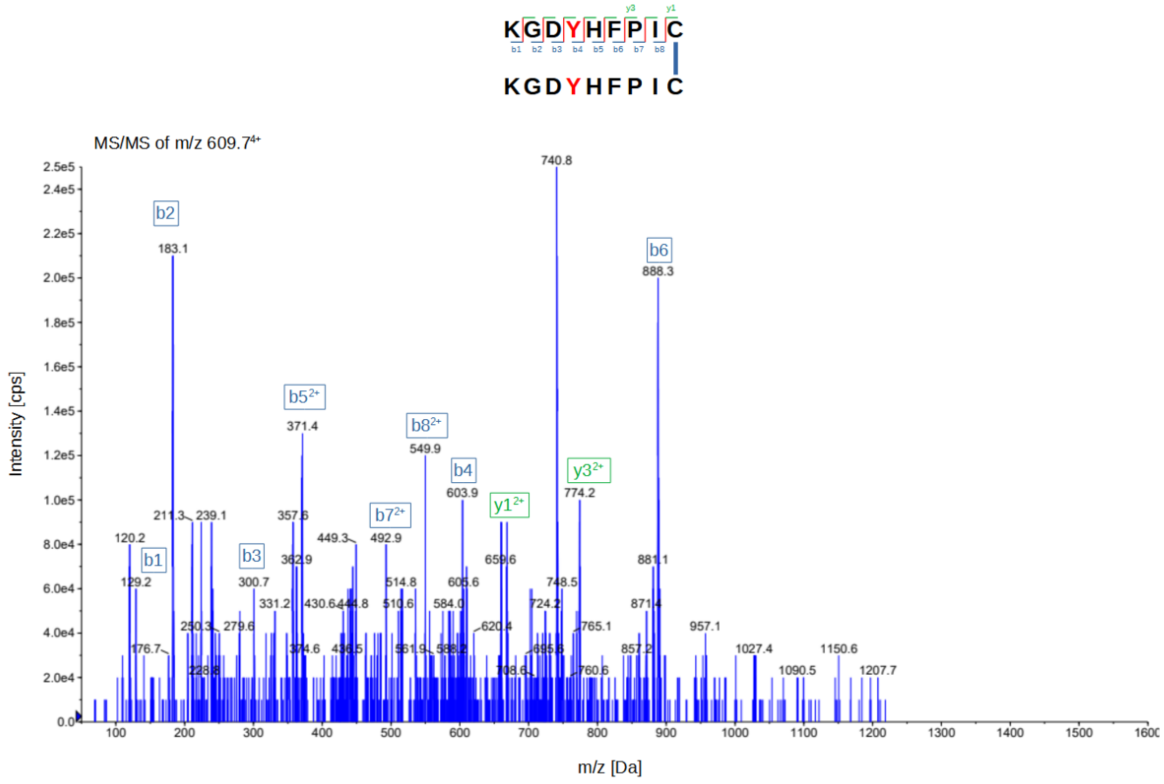

KGDYHFPIC
KGDYHFPIC
MS/MS of m/z 609.7⁴⁺
Intensity [cps]
m/z [Da]
b2
183.1
b6
888.3
740.8
b5²⁺
371.4
b8²⁺
549.9
b4
603.9
y3²⁺
774.2
y1²⁺
b7²⁺
492.9
b1
b3
300.7
120.2
129.2
211.3
239.1
357.6
449.3
362.9
659.6
881.1
514.8
605.6
748.5
871.4
331.2
430.6
444.8
510.6
584.0
724.2
250.3
279.6
620.4
765.1
957.1
176.7
374.6
436.5
561.9
588.2
695.8
857.2
1027.4
1150.6
228.8
708.6
760.6
1090.5
1207.7

NP+2

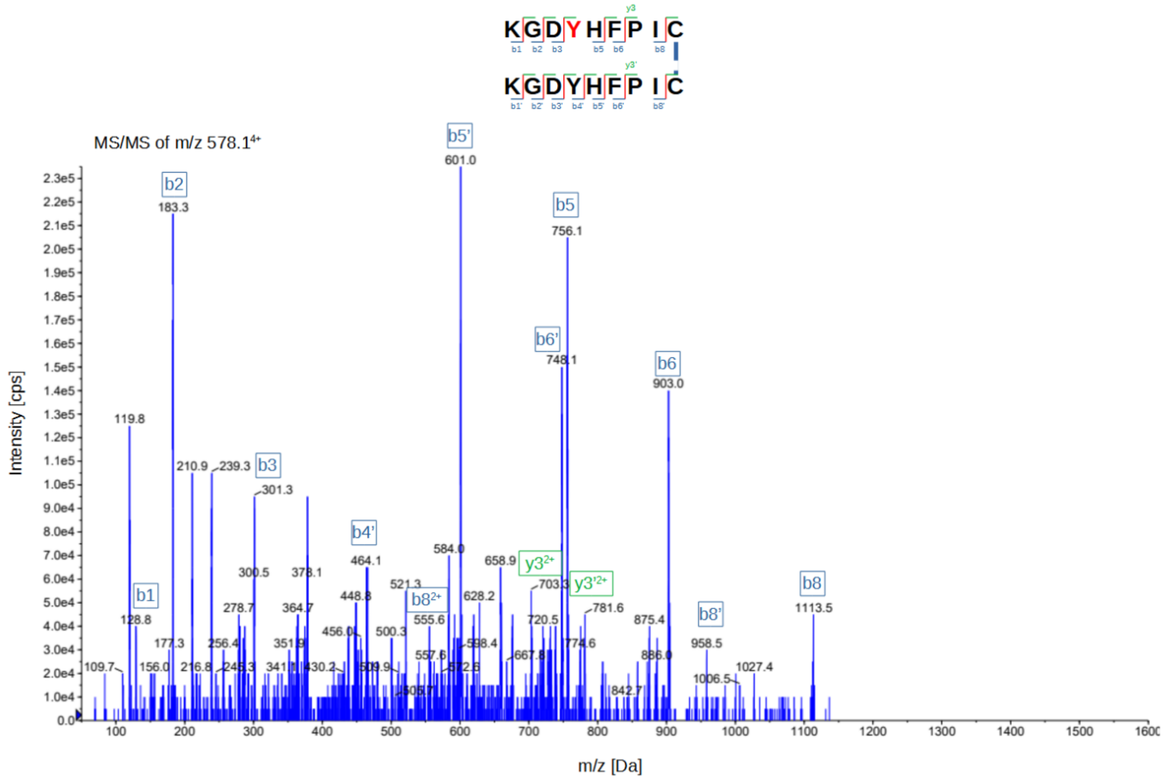
KGDYHFPIC
KGDYHFPIC
MS/MS of m/z 578.1⁴⁺
Intensity [cps]
m/z [Da]
b2 183.3
b5' 601.0
b5 756.1
b6' 748.1
b6 903.0
b3 301.3
b4' 464.1
b1 128.8
b8²⁺ 555.6
y3²⁺ 703.3
y3'²⁺
b8' 958.5
b8 1113.5
119.8
210.9
239.3
584.0
658.9
781.6
875.4
1027.4

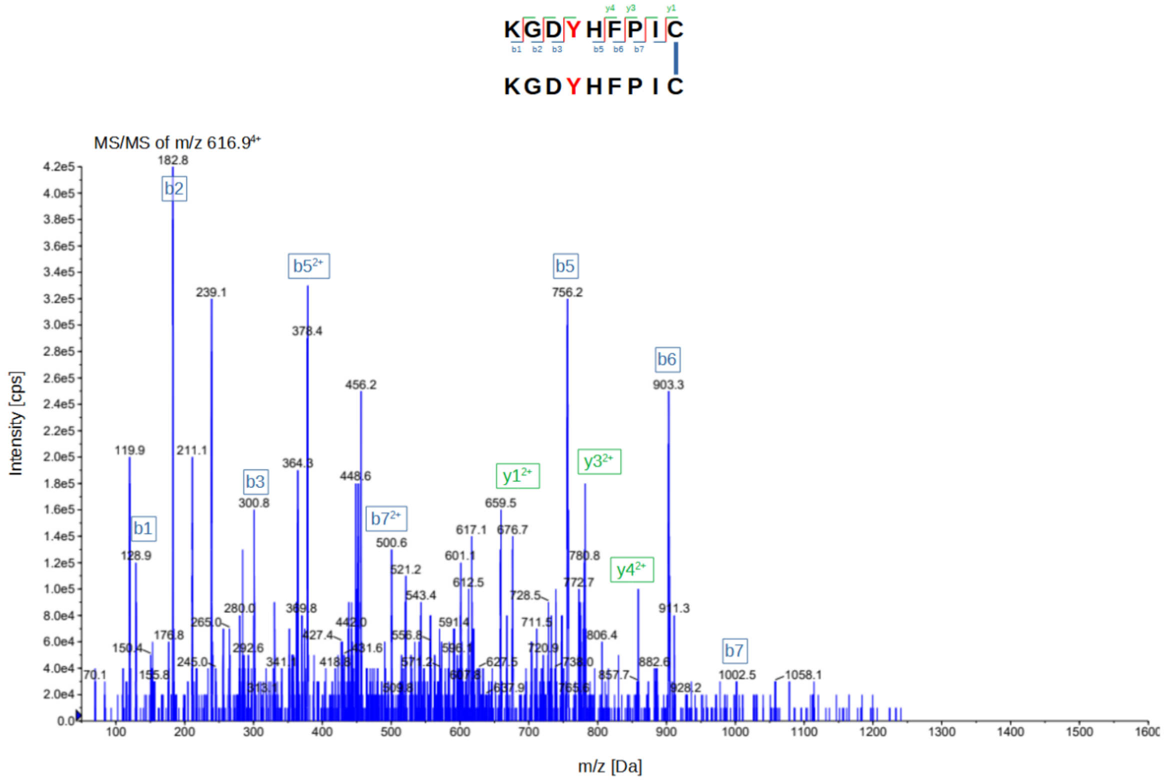
KGDYHFPIC
KGDYHFPIC
MS/MS of m/z 616.9⁴⁺
Intensity [cps]
m/z [Da]
b2 182.8
b5²⁺ 378.4
b5 756.2
b6 903.3
b3 300.8
b1 128.9
b7²⁺ 500.6
y1²⁺ 659.5
y3²⁺ 780.8
y4²⁺
b7 1002.5
239.1
456.2
119.9
211.1
364.8
448.6
617.1
676.7
1058.1

NP+4

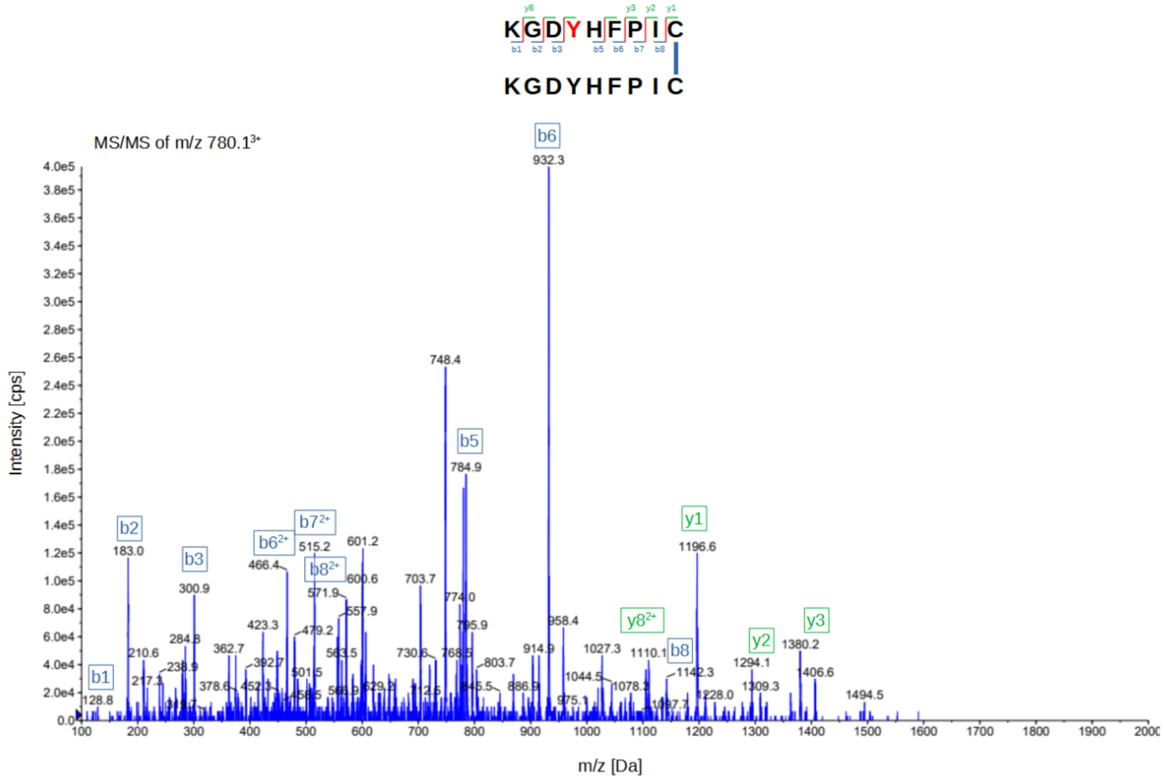

KGDYHFPIC
KGDYHFPIC
MS/MS of m/z 780.1³⁺
Intensity [cps]
m/z [Da]
b6
932.3
748.4
b5
784.9
b2
183.0
b3
300.9
b1
b6²⁺
b7²⁺
515.2
b8²⁺
601.2
703.7
y1
1196.6
y8²⁺
b8
y2
y3
1380.2
1294.1
1406.6
1494.5

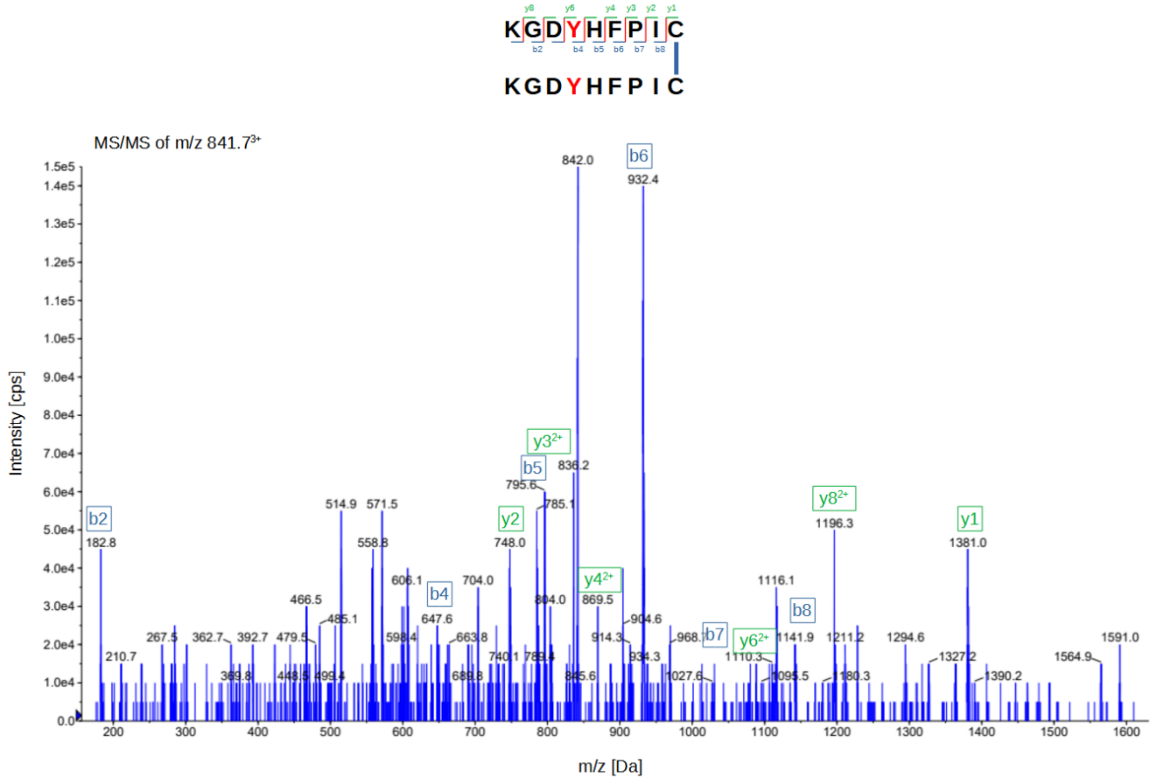

KGDYHFPIC
KGDYHFPIC
MS/MS of m/z 841.7³⁺
Intensity [cps]
m/z [Da]
842.0
b6
932.4
y3²⁺
b5
836.2
795.6
785.1
y2
748.0
b2
182.8
514.9
571.5
y8²⁺
1196.3
y1
1381.0
b4
y4²⁺
b7
y6²⁺
b8
1116.1
1591.0

NP+5

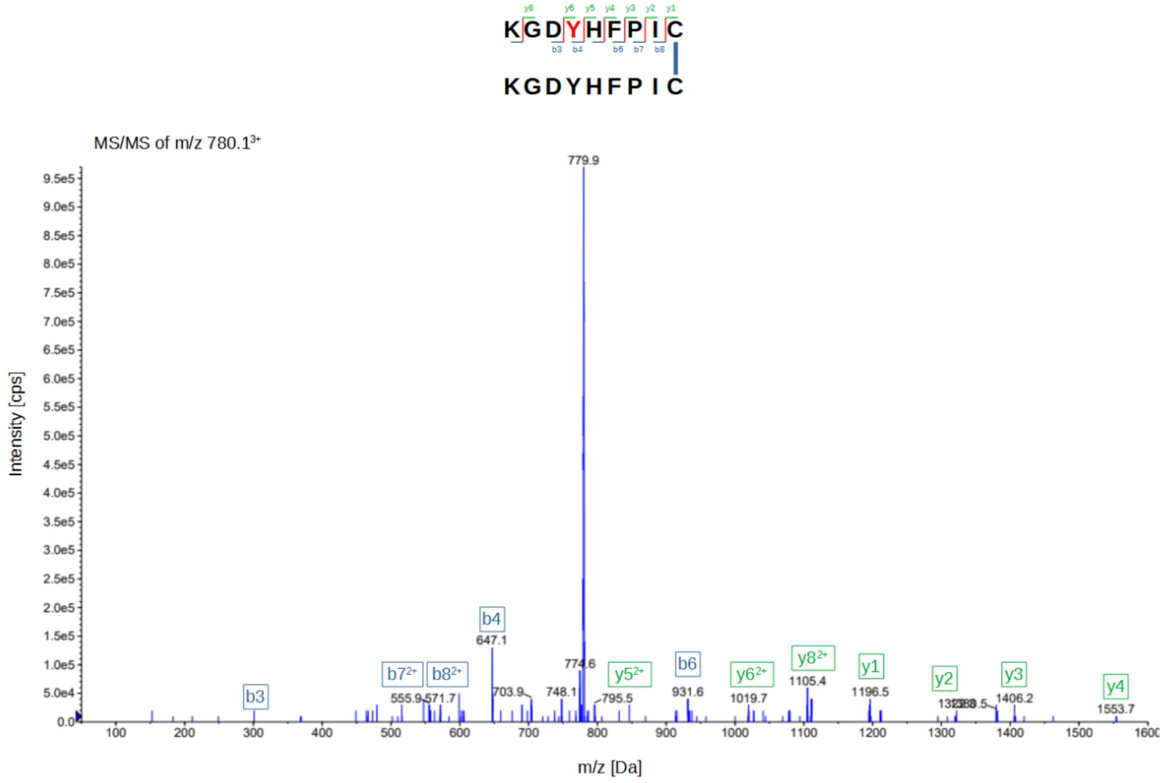

KGDYHFPIC
KGDYHFPIC
MS/MS of m/z 780.1³⁺
Intensity [cps]
m/z [Da]
779.9
b4
647.1
b3
b7²⁺
b8²⁺
555.9
571.7
703.9
748.1
774.6
y5²⁺
795.5
b6
931.6
y6²⁺
1019.7
y8²⁺
1105.4
y1
1196.5
y2
y3
1406.2
y4
1553.7

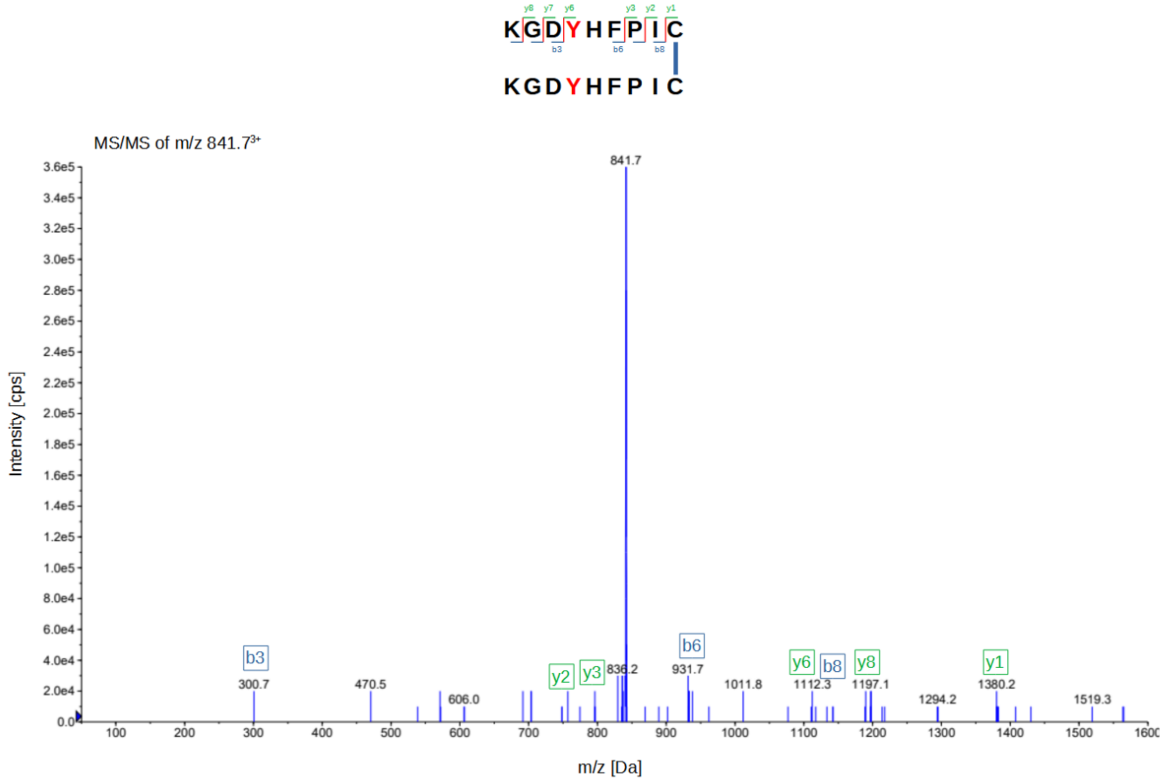

KGDYHFPIC
KGDYHFPIC
MS/MS of m/z 841.7³⁺
Intensity [cps]
m/z [Da]
841.7
b3
300.7
470.5
606.0
y2
y3
836.2
b6
931.7
1011.8
y6
1112.3
b8
y8
1197.1
1294.2
y1
1380.2
1519.3

NP+6

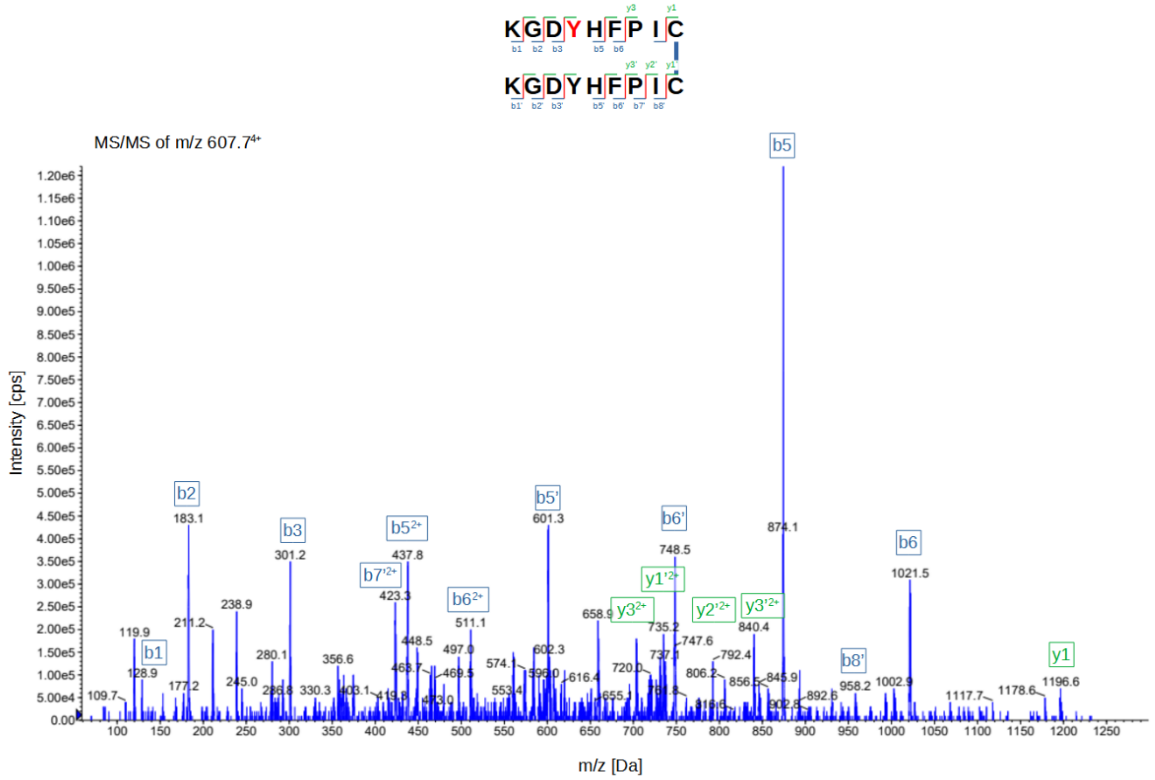

KGDYHFPIC
KGDYHFPIC
MS/MS of m/z 607.7 4+
Intensity [cps]
m/z [Da]
b1
b2
183.1
b3
301.2
b5 2+
437.8
b7' 2+
423.3
b6 2+
511.1
b5'
601.3
b6'
748.5
y3 2+
658.9
y1' 2+
735.2
y2' 2+
y3' 2+
840.4
b5
874.1
b8'
958.2
b6
1021.5
y1
1196.6

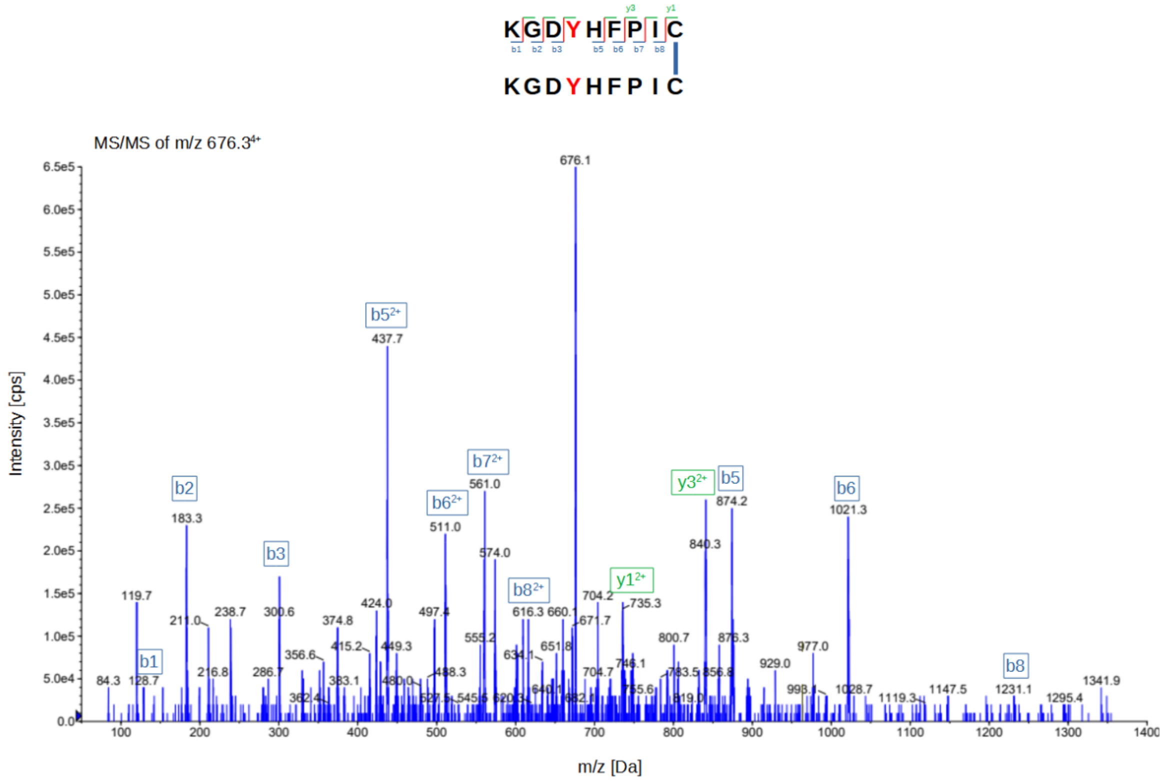

KGDYHFPIC
KGDYHFPIC
MS/MS of m/z 676.3 4+
Intensity [cps]
m/z [Da]
676.1
b1
128.7
b2
183.3
b3
300.6
b5 2+
437.7
b6 2+
511.0
b7 2+
561.0
b8 2+
616.3
y1 2+
735.3
y3 2+
840.3
b5
874.2
b6
1021.3
b8
1231.1

NP+7

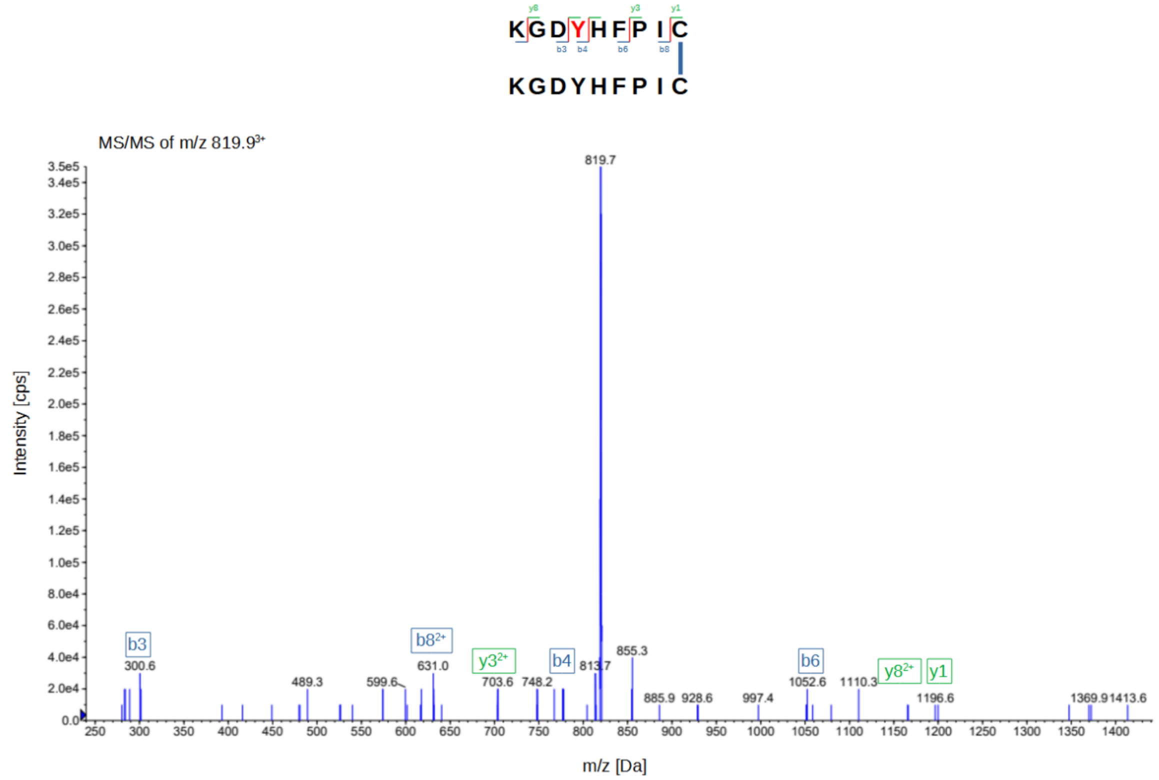

KGDYHFPIC
KGDYHFPIC
MS/MS of m/z 819.9³⁺
Intensity [cps]
m/z [Da]
819.7
b3
300.6
b8²⁺
631.0
y3²⁺
703.6
b4
748.2
813.7
855.3
b6
1052.6
1110.3
y8²⁺
y1
1196.6
489.3
599.6
885.9
928.6
997.4
1369.9
1413.6

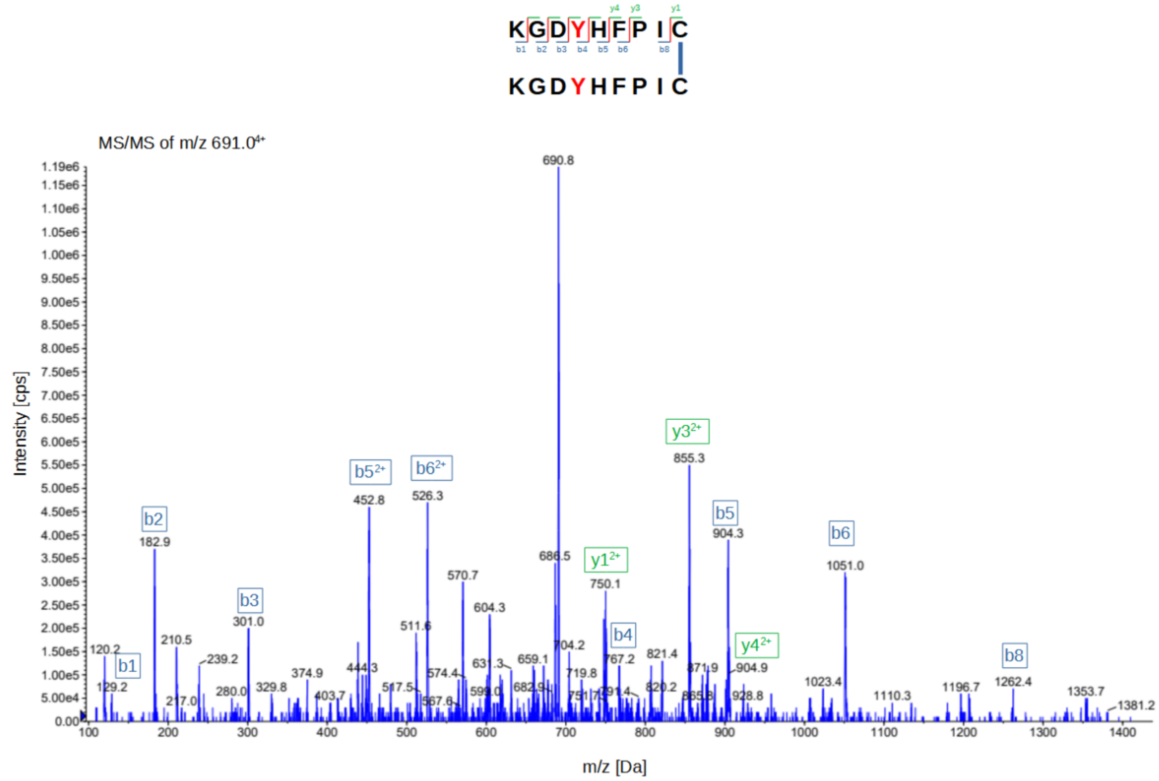

KGDYHFPIC
KGDYHFPIC
MS/MS of m/z 691.0⁴⁺
Intensity [cps]
m/z [Da]
690.8
y3²⁺
855.3
b5²⁺
452.8
b6²⁺
526.3
b2
182.9
b5
904.3
b6
1051.0
y1²⁺
750.1
686.5
570.7
604.3
b3
301.0
511.6
b4
767.2
y4²⁺
904.9
b8
1262.4
b1
120.2
129.2
210.5
239.2
280.0
329.8
374.9
403.7
444.3
517.5
567.6
574.4
599.0
631.3
659.1
682.9
704.2
719.8
751.7
791.4
820.2
821.4
865.8
871.9
928.8
1023.4
1110.3
1196.7
1353.7
1381.2
217.0

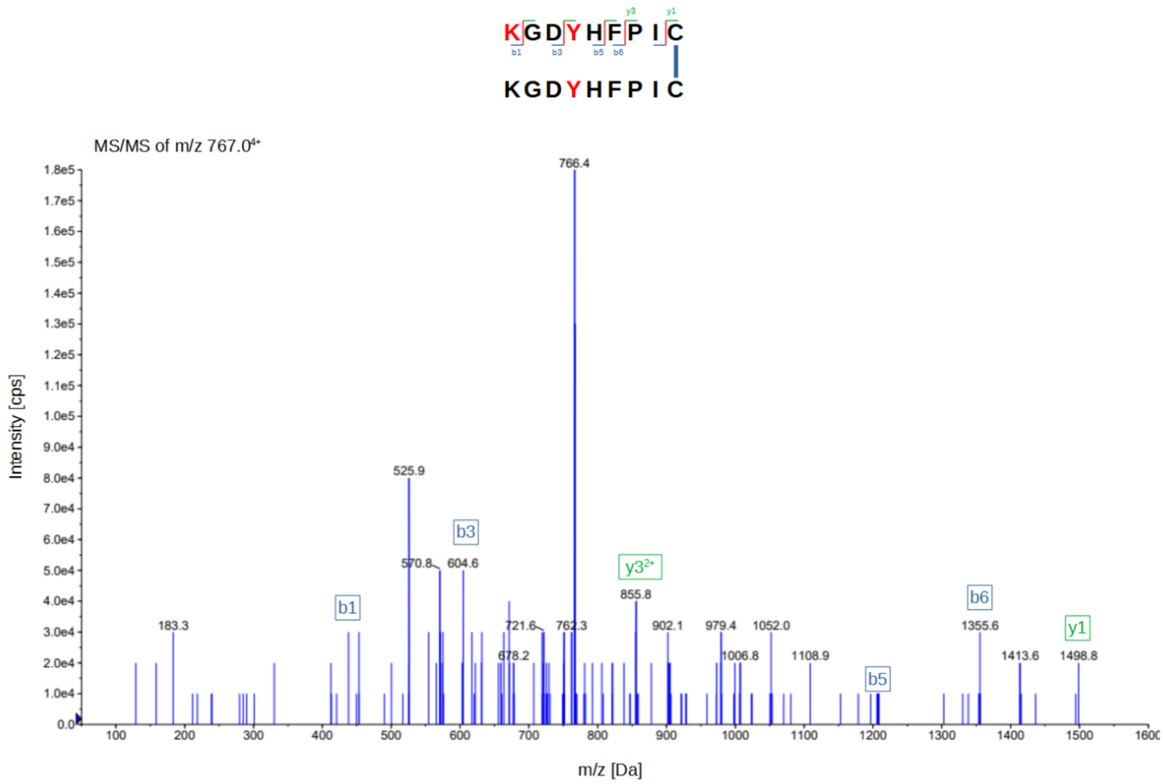

KGDYHFPIC
KGDYHFPIC
MS/MS of m/z 767.0 4+
Intensity [cps]
m/z [Da]
766.4
525.9
570.8
604.6
b1
b3
y3 2+
855.8
183.3
721.6
762.3
678.2
902.1
979.4
1006.8
1052.0
1108.9
b5
b6
1355.6
1413.6
y1
1498.8

NP+8

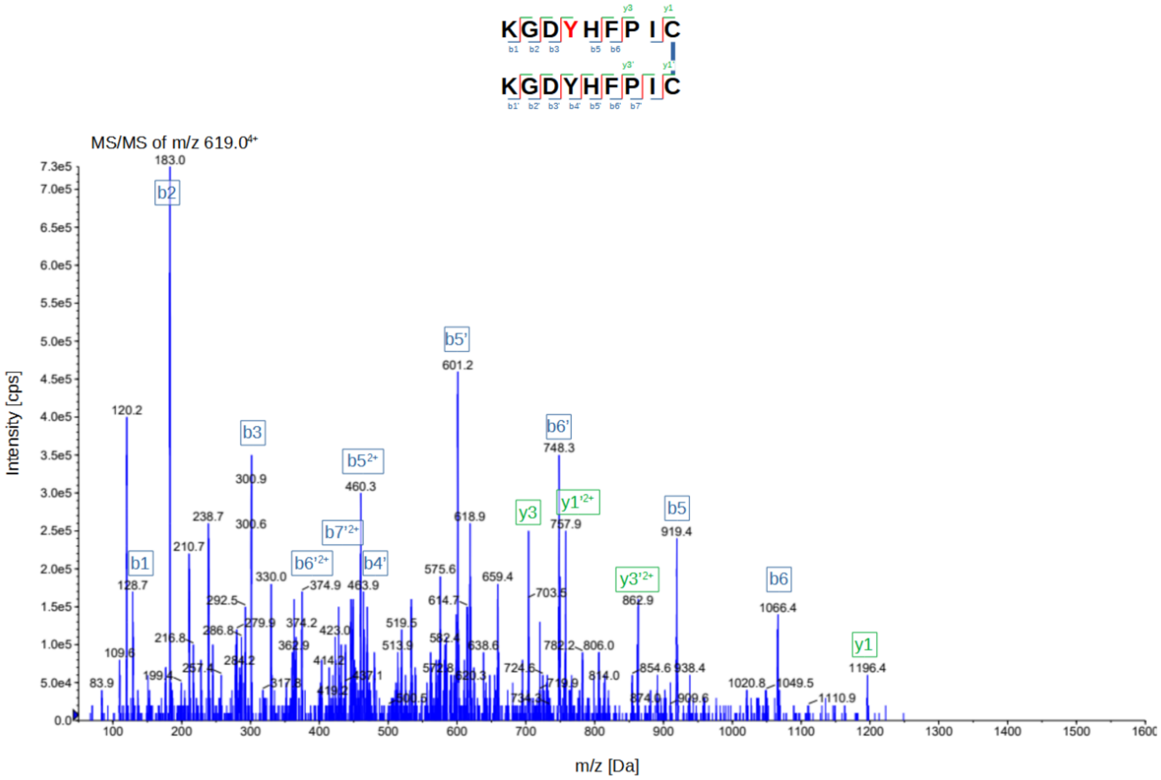
KGDYHFPIC
KGDYHFPIC
MS/MS of m/z 619.0⁴⁺
Intensity [cps]
m/z [Da]
b2
b5'
b6'
b3
b5²⁺
y3
y1'²⁺
b5
b1
b7'²⁺
b6'²⁺
b4'
y3'²⁺
b6
y1

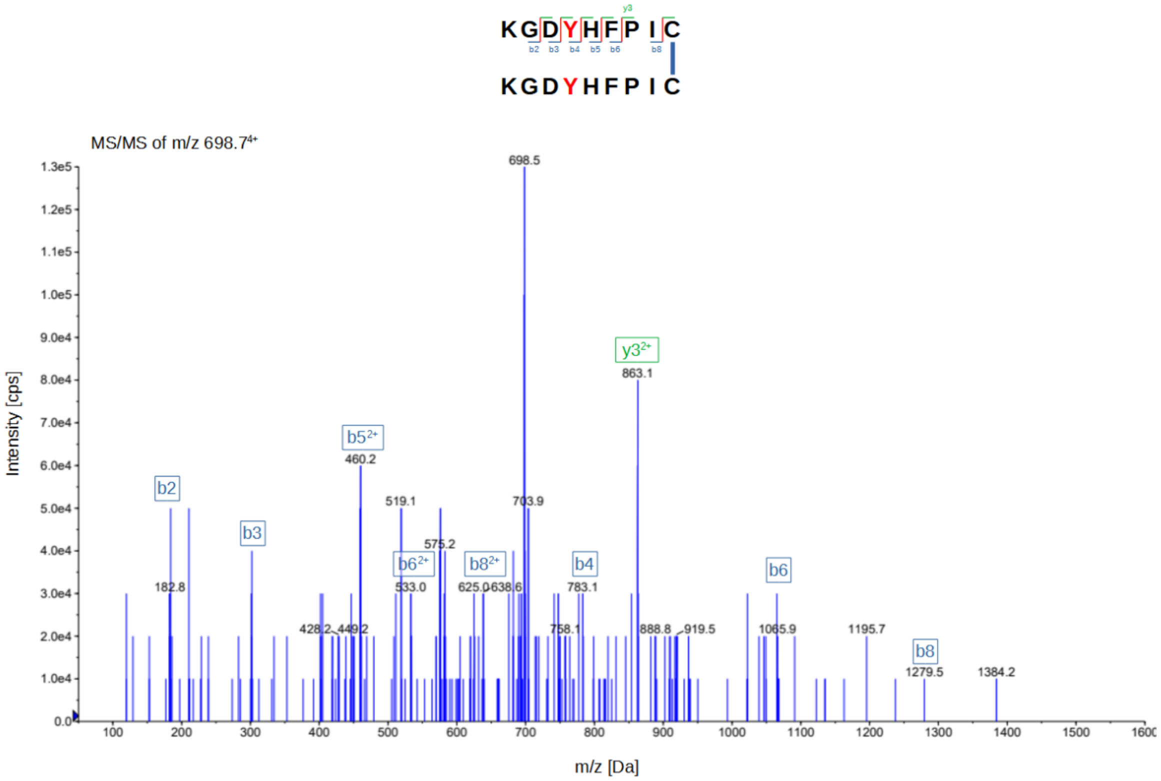
KGDYHFPIC
KGDYHFPIC
MS/MS of m/z 698.7⁴⁺
Intensity [cps]
m/z [Da]
y3²⁺
b5²⁺
b2
b3
b6²⁺
b8²⁺
b4
b6
b8

NP+9

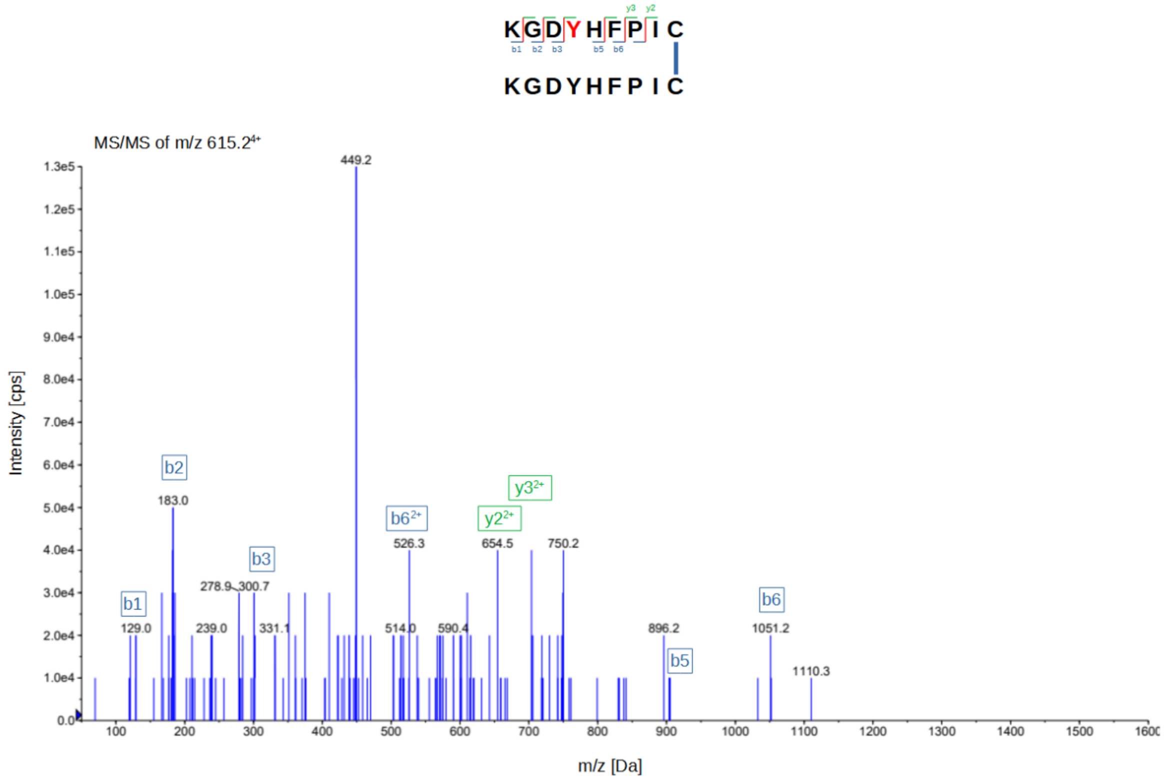

KGDYHFPIC
KGDYHFPIC
MS/MS of m/z 615.2⁴⁺
Intensity [cps]
m/z [Da]
449.2
b2
183.0
b6²⁺
526.3
y3²⁺
y2²⁺
654.5
750.2
b3
278.9
300.7
b1
129.0
239.0
331.1
514.0
590.4
896.2
b6
1051.2
b5
1110.3

NP+10

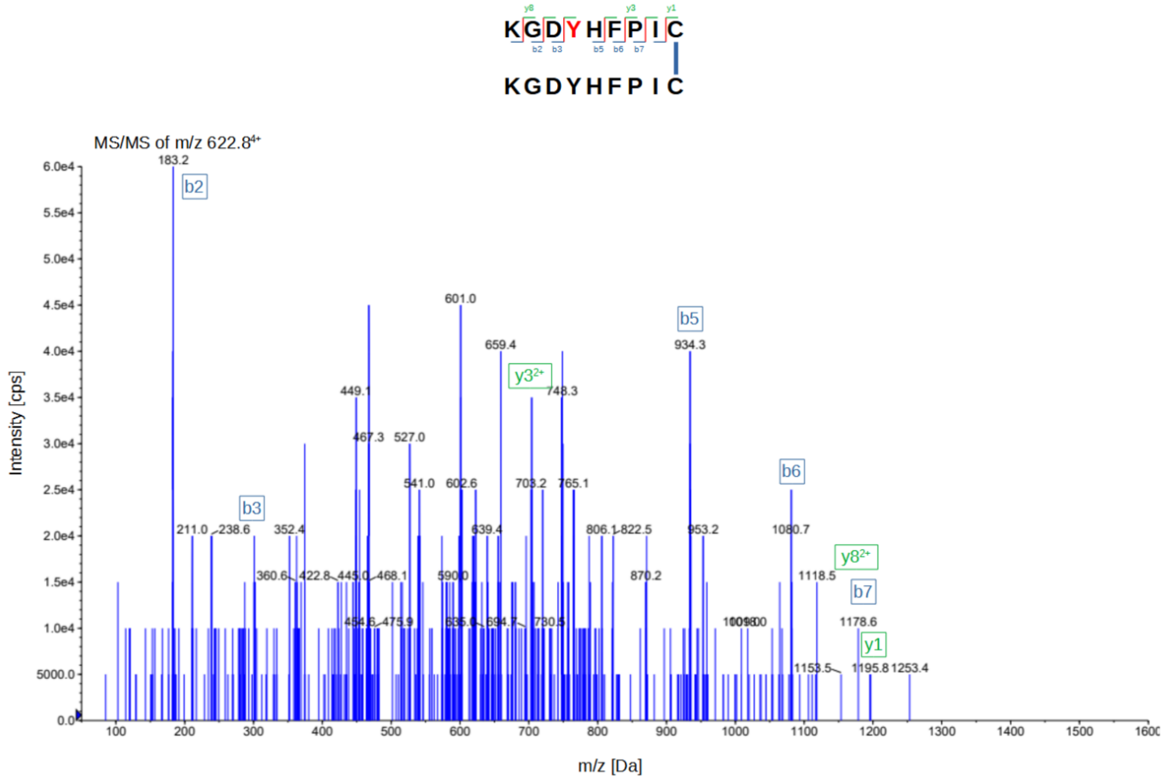
KGDYHFPIC
KGDYHFPIC
MS/MS of m/z 622.8⁴⁺
Intensity [cps]
m/z [Da]
b2
b3
b5
b6
b7
y1
183.2
601.0
659.4
934.3
748.3
1080.7
1118.5
1178.6
1253.4

NP+12

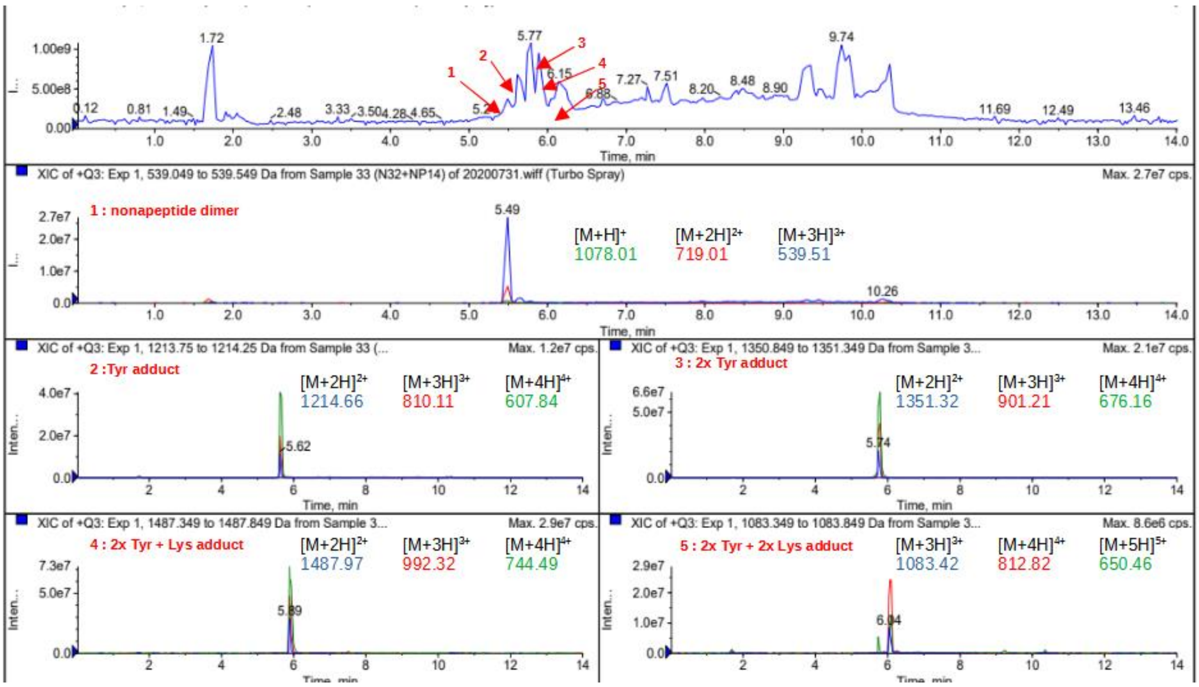
1 : nonapeptide dimer
[M+H]⁺ 1078.01
[M+2H]²⁺ 719.01
[M+3H]³⁺ 539.51
2 :Tyr adduct
[M+2H]²⁺ 1214.66
[M+3H]³⁺ 810.11
[M+4H]⁴⁺ 607.84
3 : 2x Tyr adduct
[M+2H]²⁺ 1351.32
[M+3H]³⁺ 901.21
[M+4H]⁴⁺ 676.16
4 : 2x Tyr + Lys adduct
[M+2H]²⁺ 1487.97
[M+3H]³⁺ 992.32
[M+4H]⁴⁺ 744.49
5 : 2x Tyr + 2x Lys adduct
[M+3H]³⁺ 1083.42
[M+4H]⁴⁺ 812.82
[M+5H]⁵⁺ 650.46
Time, min

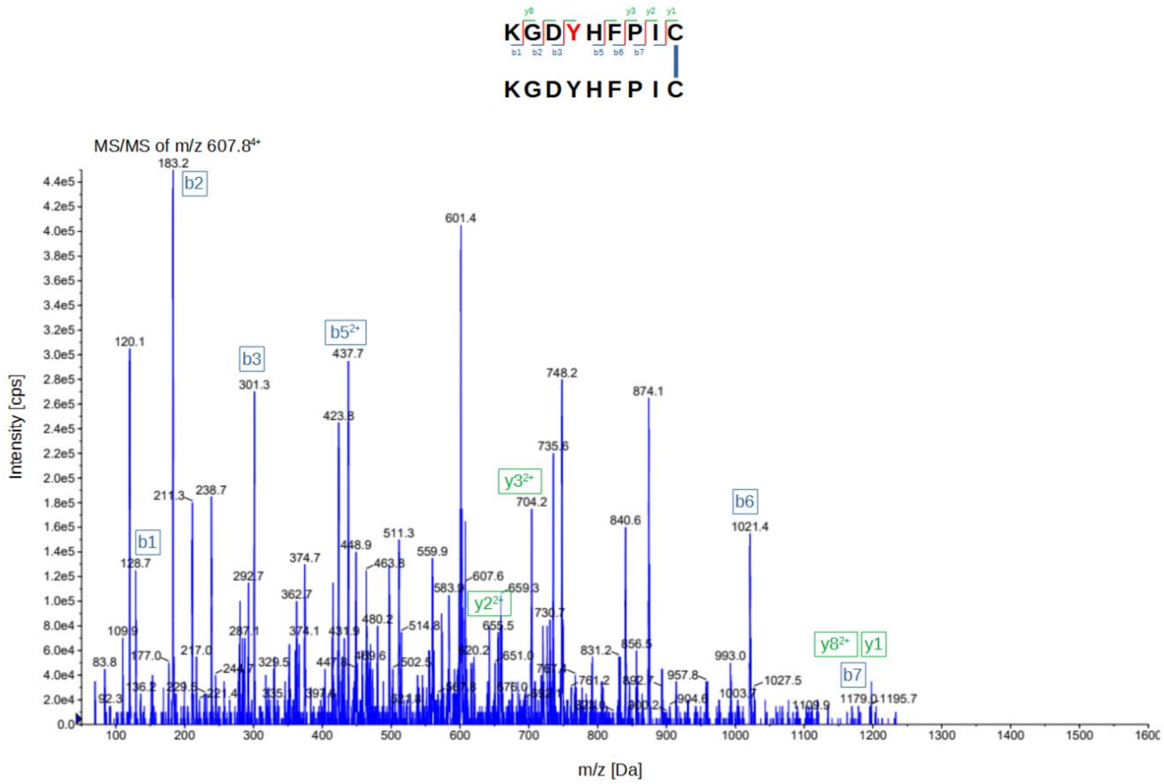
KGDYHFPIC
KGDYHFPIC
MS/MS of m/z 607.8⁴⁺
Intensity [cps]
m/z [Da]
b1
b2
b3
b5²⁺
y3²⁺
y2²⁺
b6
y8²⁺
y1
b7
183.2
601.4
120.1
437.7
301.3
423.8
748.2
874.1
735.6
704.2
1021.4
840.6

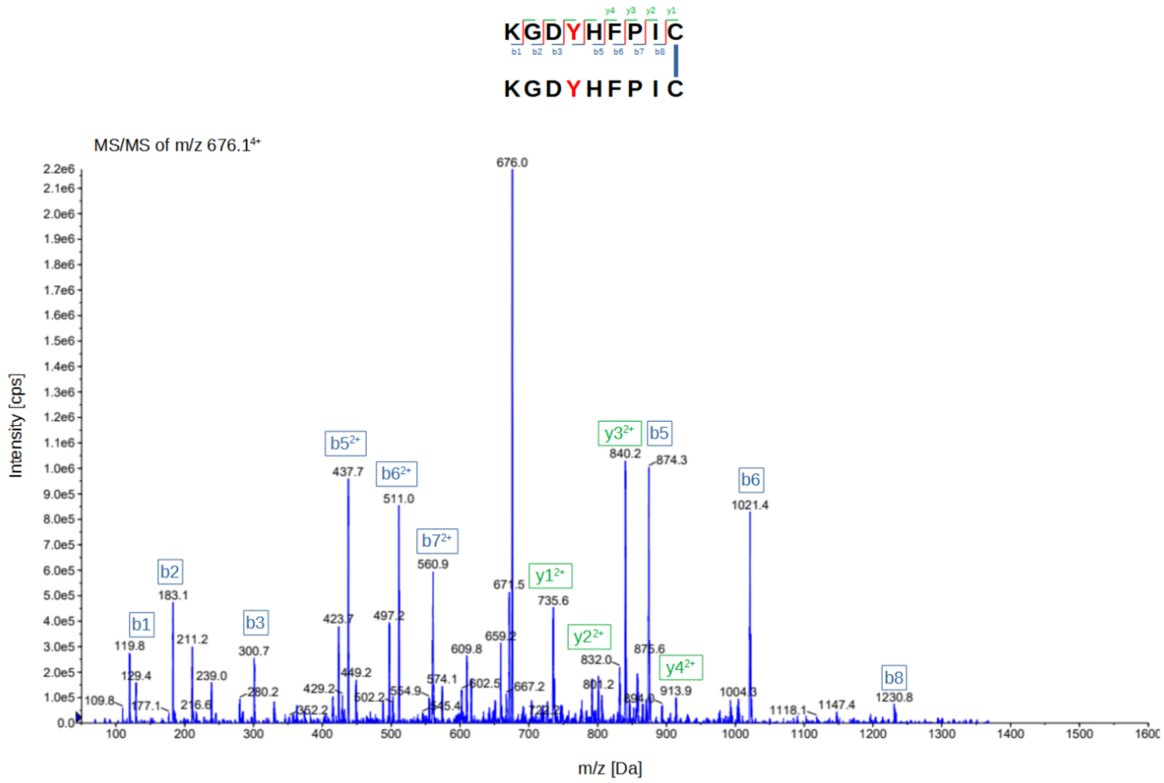
KGDYHFPIC
KGDYHFPIC
MS/MS of m/z 676.1⁴⁺
Intensity [cps]
m/z [Da]
b1
b2
b3
b5²⁺
b6²⁺
b7²⁺
y1²⁺
y2²⁺
y3²⁺
b5
y4²⁺
b6
b8
676.0
437.7
511.0
560.9
840.2
874.3
1021.4
735.6
183.1
1230.8

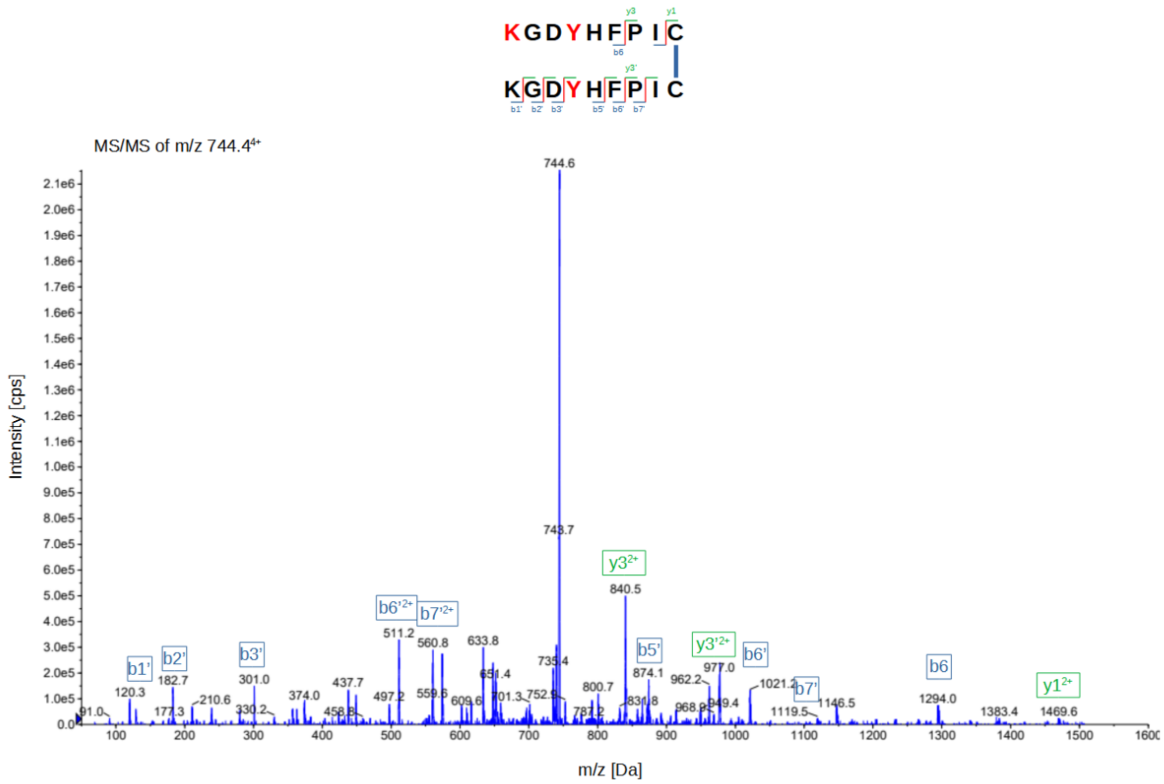

KGDYHFPIC
KGDYHFPIC
MS/MS of m/z 744.4 4+
Intensity [cps]
m/z [Da]
744.6
743.7
y3 2+
840.5
b6' 2+
b7' 2+
511.2
560.8
633.8
735.4
651.4
b1'
b2'
b3'
120.3
182.7
301.0
437.7
374.0
b5'
874.1
y3' 2+
977.0
b6'
962.2
1021.2
b7'
b6
1294.0
y1 2+
1469.6
1383.4
1146.5
1119.5
800.7

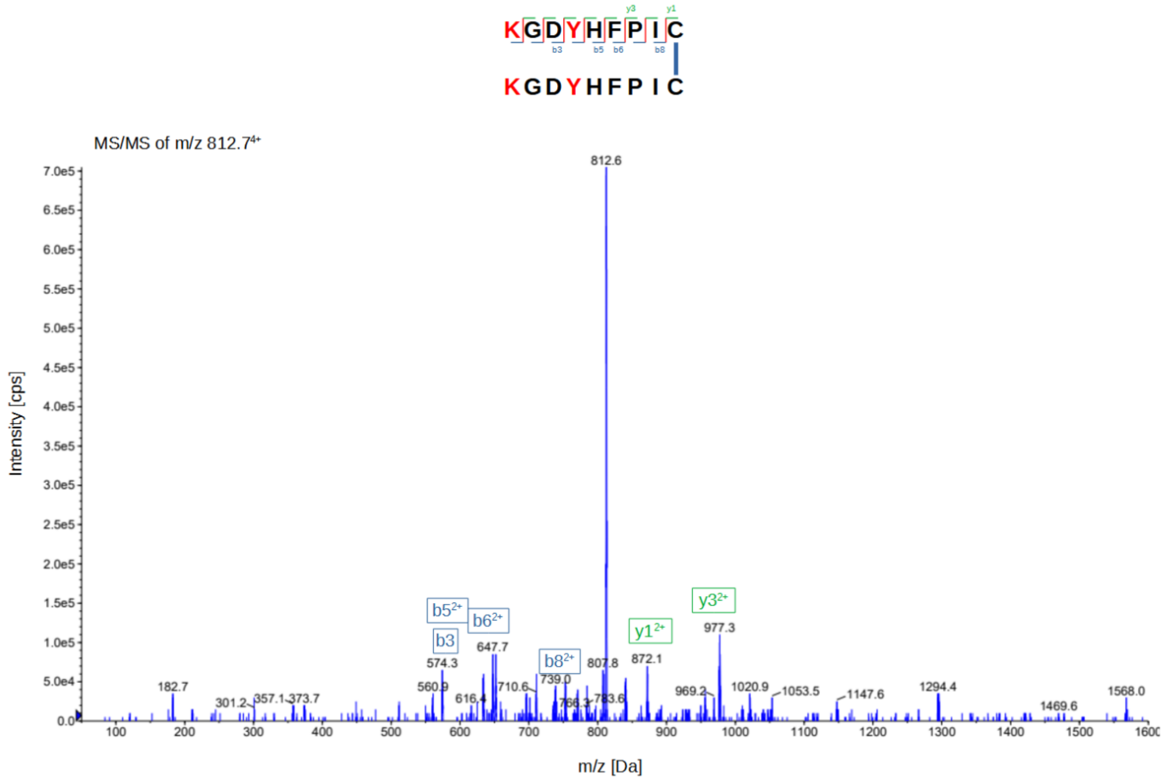

KGDYHFPIC
KGDYHFPIC
MS/MS of m/z 812.7 4+
Intensity [cps]
m/z [Da]
812.6
y3 2+
977.3
y1 2+
872.1
b5 2+
b6 2+
b3
647.7
574.3
b8 2+
807.8
739.0
182.7
301.2
357.1
373.7
1020.9
1053.5
1147.6
1294.4
1469.6
1568.0

NP+13

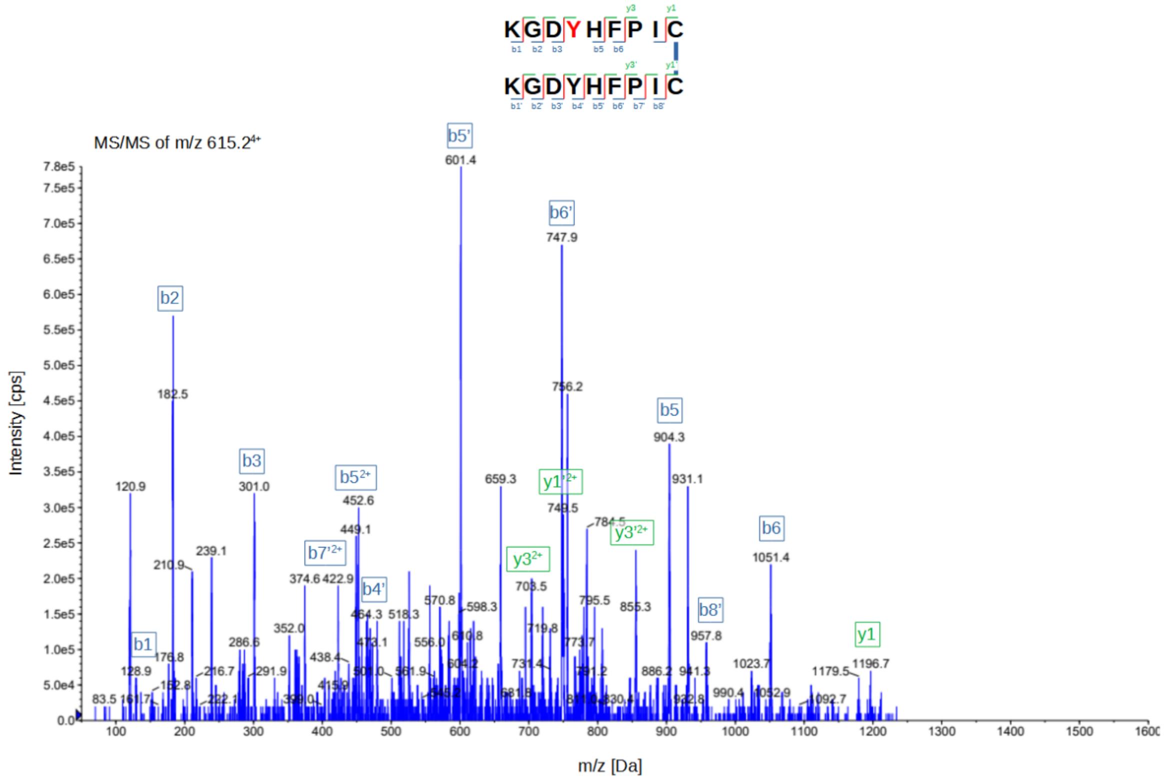

KGDYHFPIC
KGDYHFPIC
MS/MS of m/z 615.2⁴⁺
Intensity [cps]
m/z [Da]

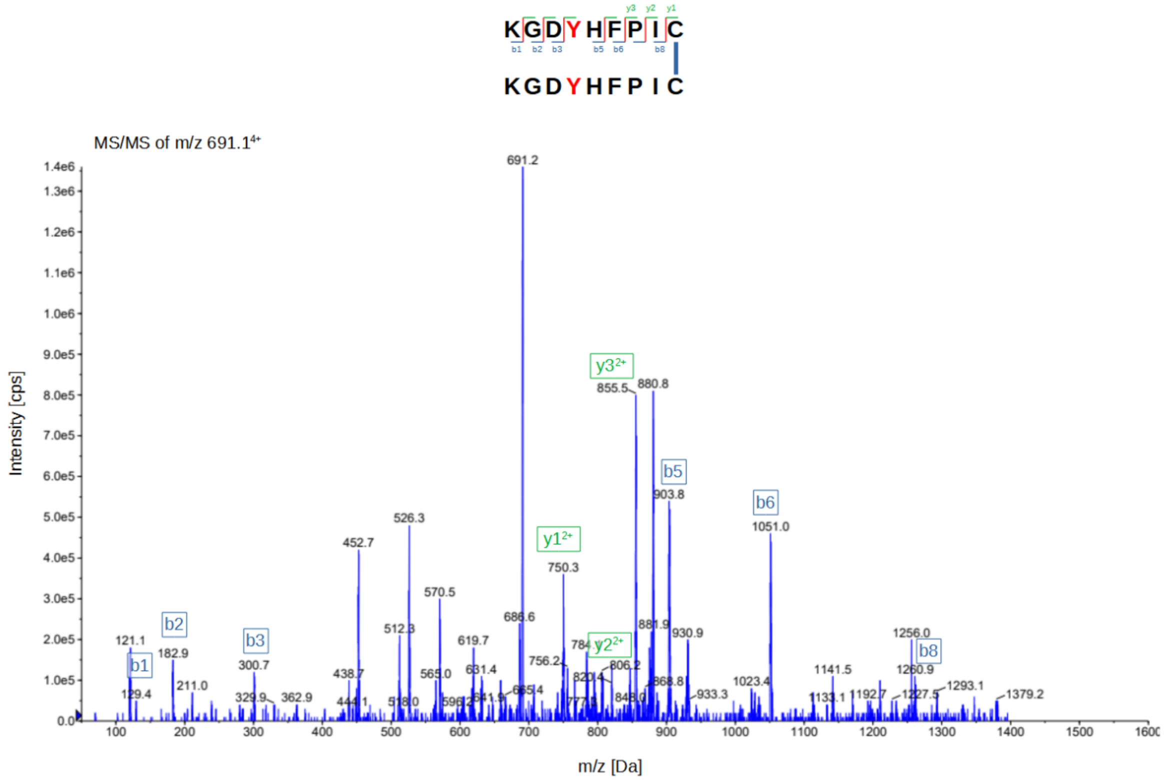

KGDYHFPIC
KGDYHFPIC
MS/MS of m/z 691.1⁴⁺
Intensity [cps]
m/z [Da]

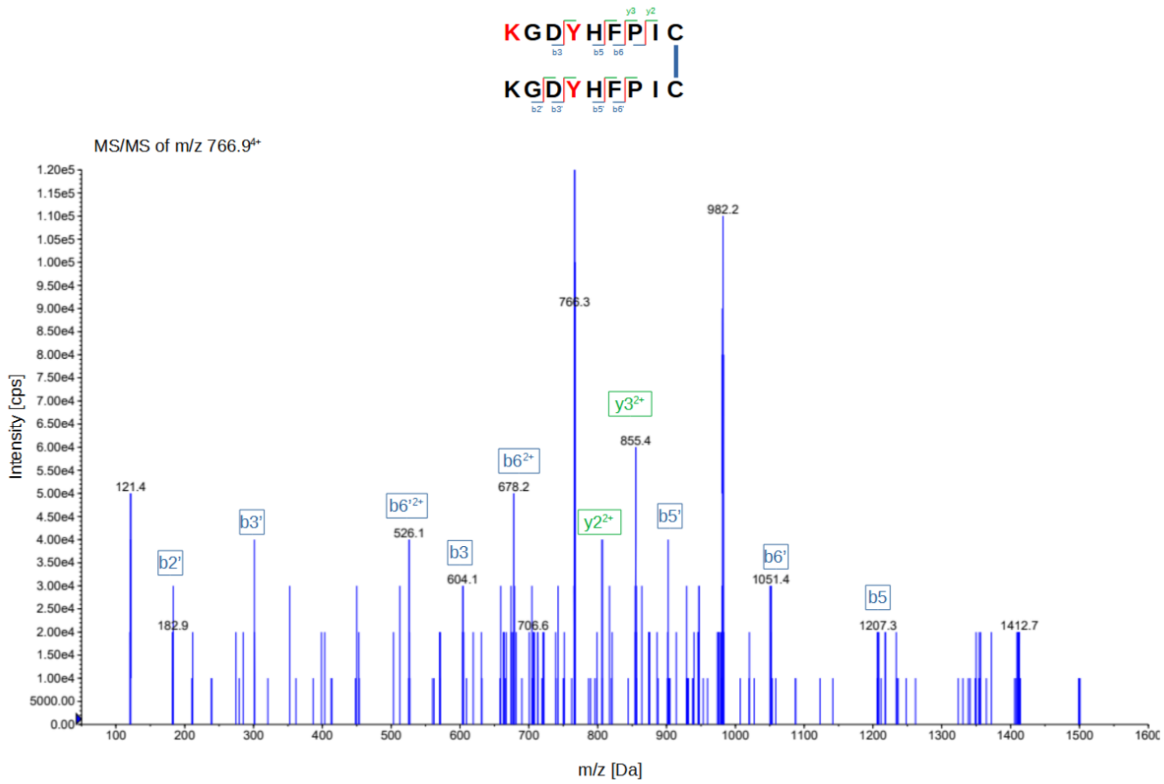
KGDYHFPIC
KGDYHFPIC
MS/MS of m/z 766.9⁴⁺
Intensity [cps]
m/z [Da]
121.4
b2'
182.9
b3'
b6'²⁺
526.1
b3
604.1
b6²⁺
678.2
706.6
766.3
y2²⁺
y3²⁺
855.4
b5'
982.2
b6'
1051.4
b5
1207.3
1412.7

NP+14

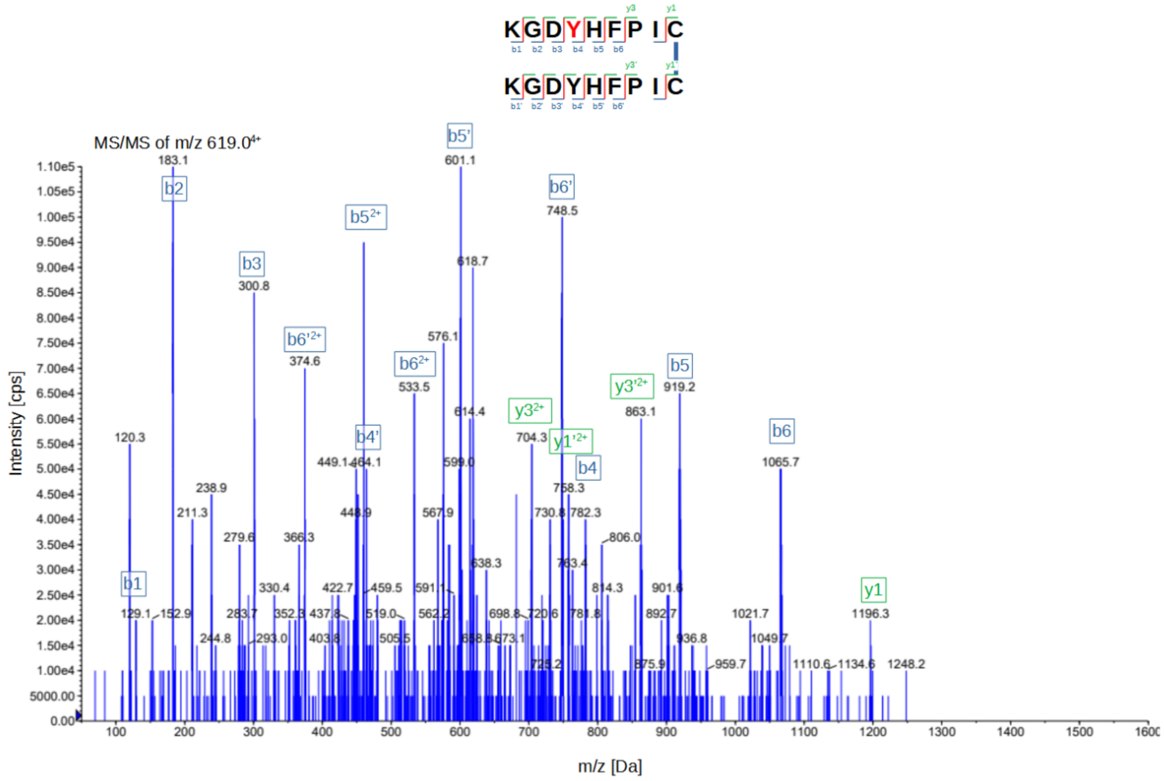
KGDYHFPIC
KGDYHFPIC
MS/MS of m/z 619.0⁴⁺
Intensity [cps]
m/z [Da]
b1
120.3
183.1
b2
211.3
238.9
b3
300.8
b6'²⁺
374.6
b5²⁺
b4'
449.1
464.1
b6²⁺
533.5
576.1
b5'
601.1
618.7
614.4
y3²⁺
704.3
y1'²⁺
b6'
748.5
b4
758.3
782.3
806.0
y3'²⁺
863.1
b5
919.2
b6
1065.7
y1
1196.3
1248.2

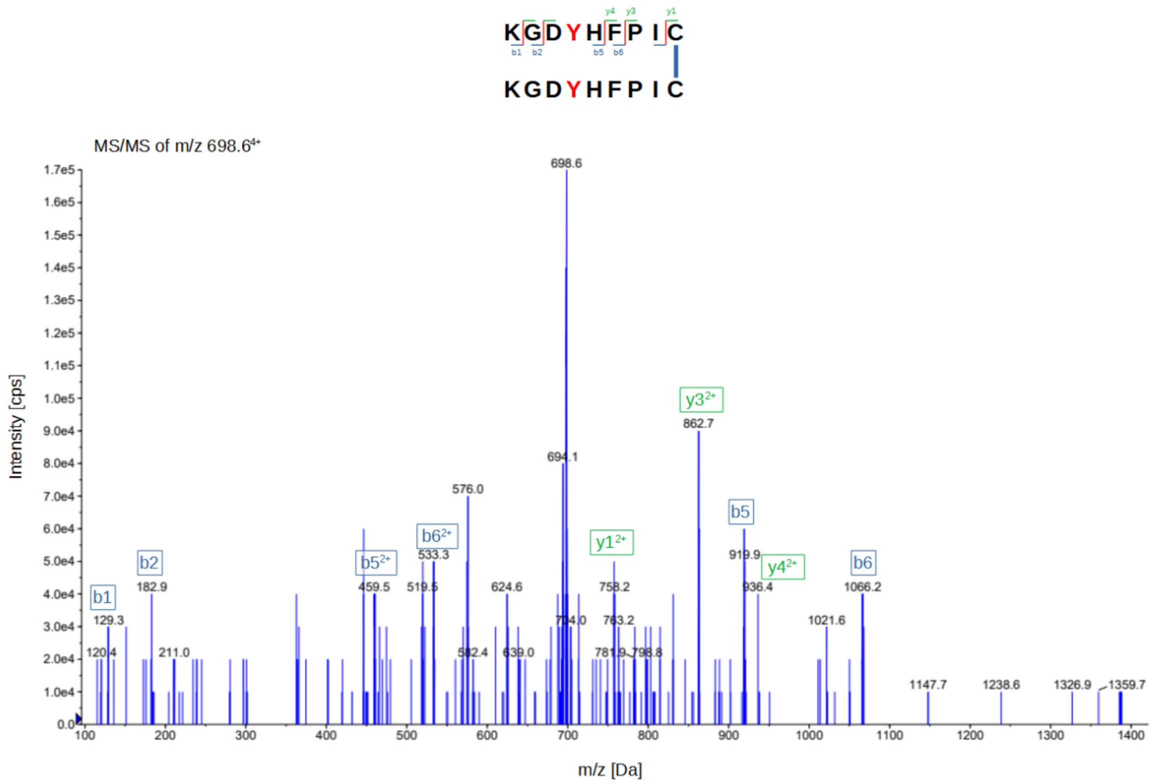
KGDYHFPIC
KGDYHFPIC
MS/MS of m/z 698.6 4+
Intensity [cps]
m/z [Da]
b1
b2
b5 2+
b6 2+
y1 2+
y3 2+
b5
y4 2+
b6
698.6
862.7
694.1
576.0
533.3
182.9
129.3
1066.2
1021.6
1147.7
1238.6
1326.9
1359.7

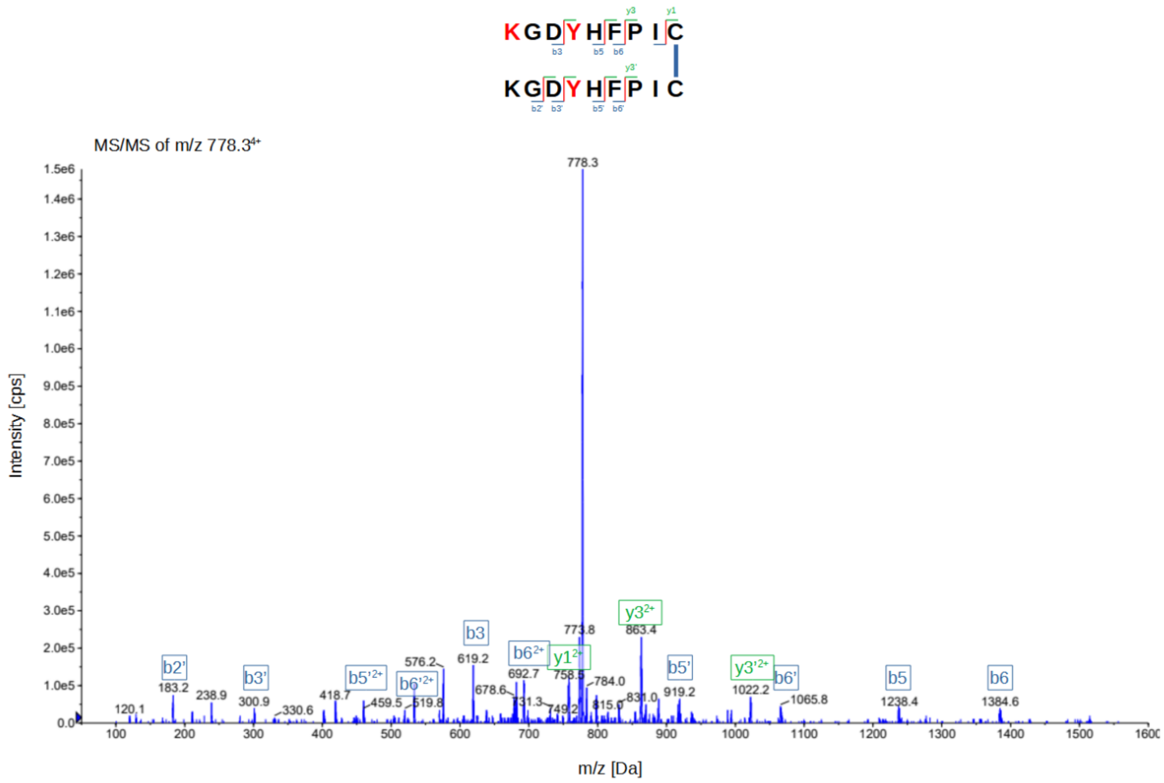
KGDYHFPIC
KGDYHFPIC
MS/MS of m/z 778.3 4+
Intensity [cps]
m/z [Da]
b2'
b3'
b5' 2+
b6' 2+
b3
b6 2+
y1 2+
y3 2+
b5'
y3' 2+
b6'
b5
b6
778.3
863.4
619.2
183.2
1238.4
1384.6

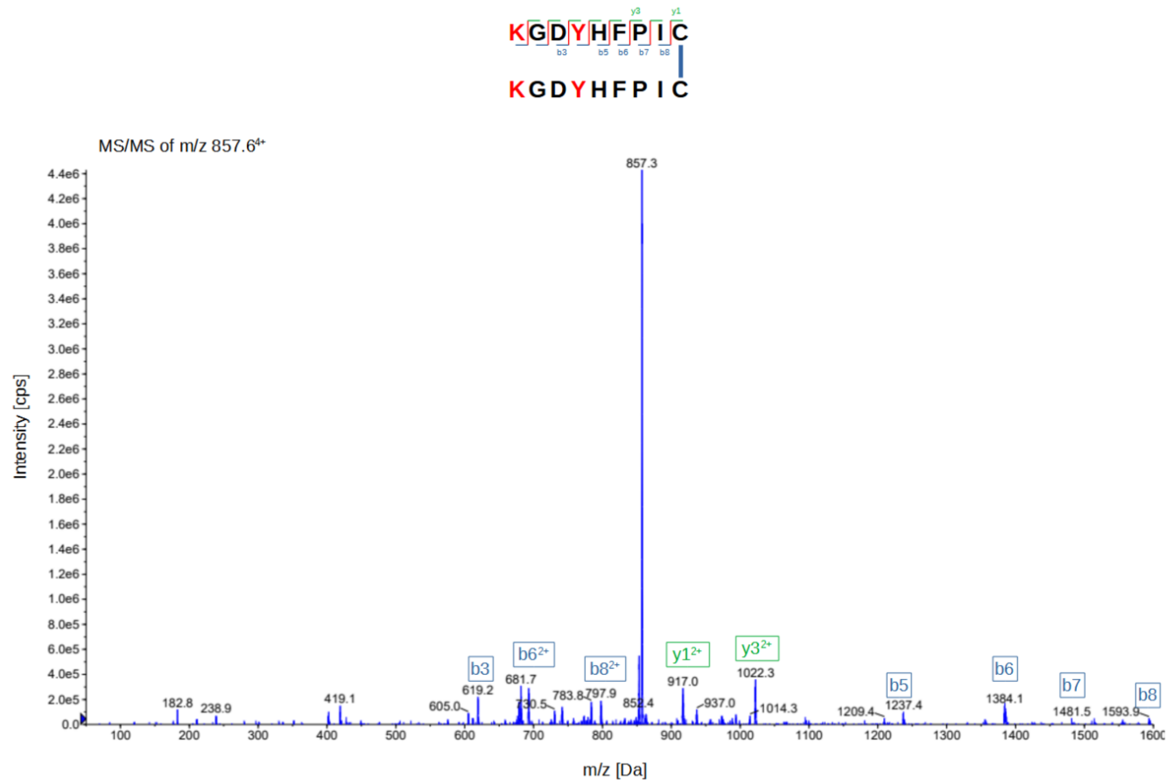
KGDYHFPIC
KGDYHFPIC
MS/MS of m/z 857.6⁴⁺
Intensity [cps]
m/z [Da]
857.3
b3
b6²⁺
b8²⁺
y1²⁺
y3²⁺
b5
b6
b7
b8
182.8
238.9
419.1
605.0
619.2
681.7
730.5
783.8
797.9
917.0
937.0
1022.3
1014.3
1209.4
1237.4
1384.1
1481.5
1593.9

NP+15

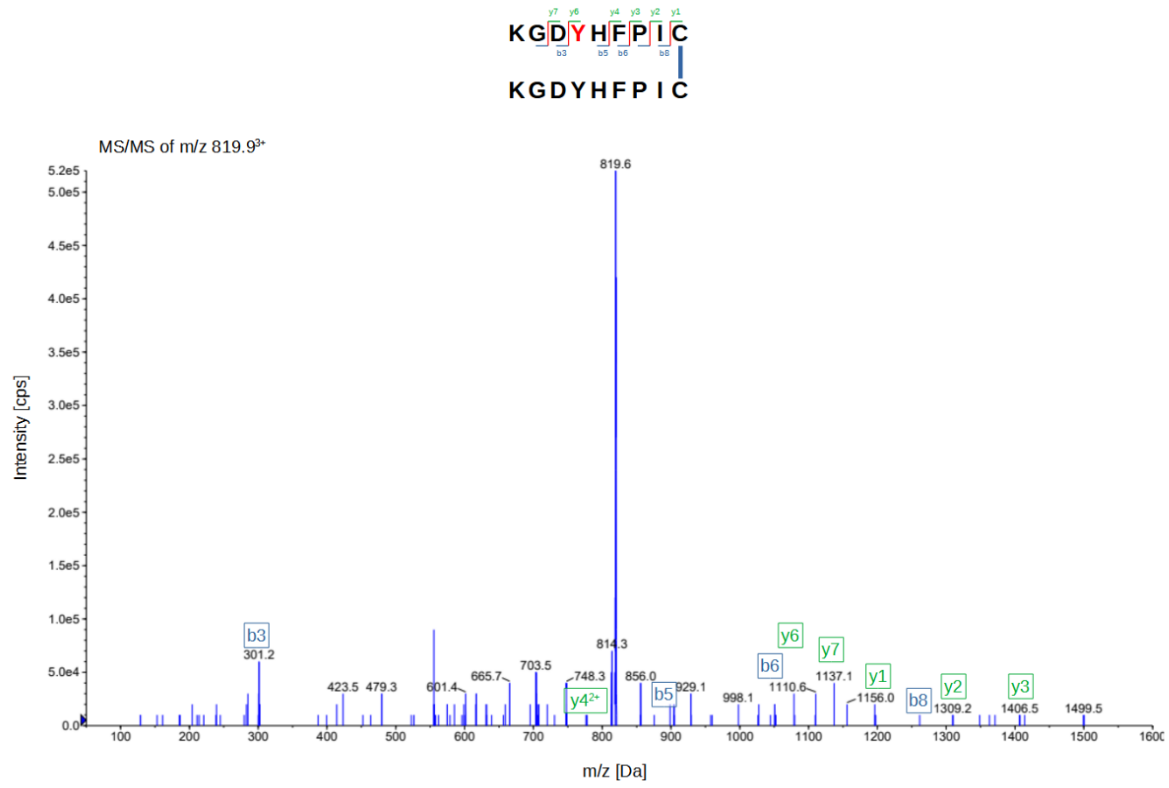
KGDYHFPIC
KGDYHFPIC
MS/MS of m/z 819.9³⁺
Intensity [cps]
m/z [Da]
819.6
b3
301.2
423.5
479.3
601.4
665.7
703.5
748.3
y4²⁺
814.3
856.0
b5
929.1
998.1
b6
1110.6
y6
y7
1137.1
1156.0
y1
b8
y2
1309.2
y3
1406.5
1499.5

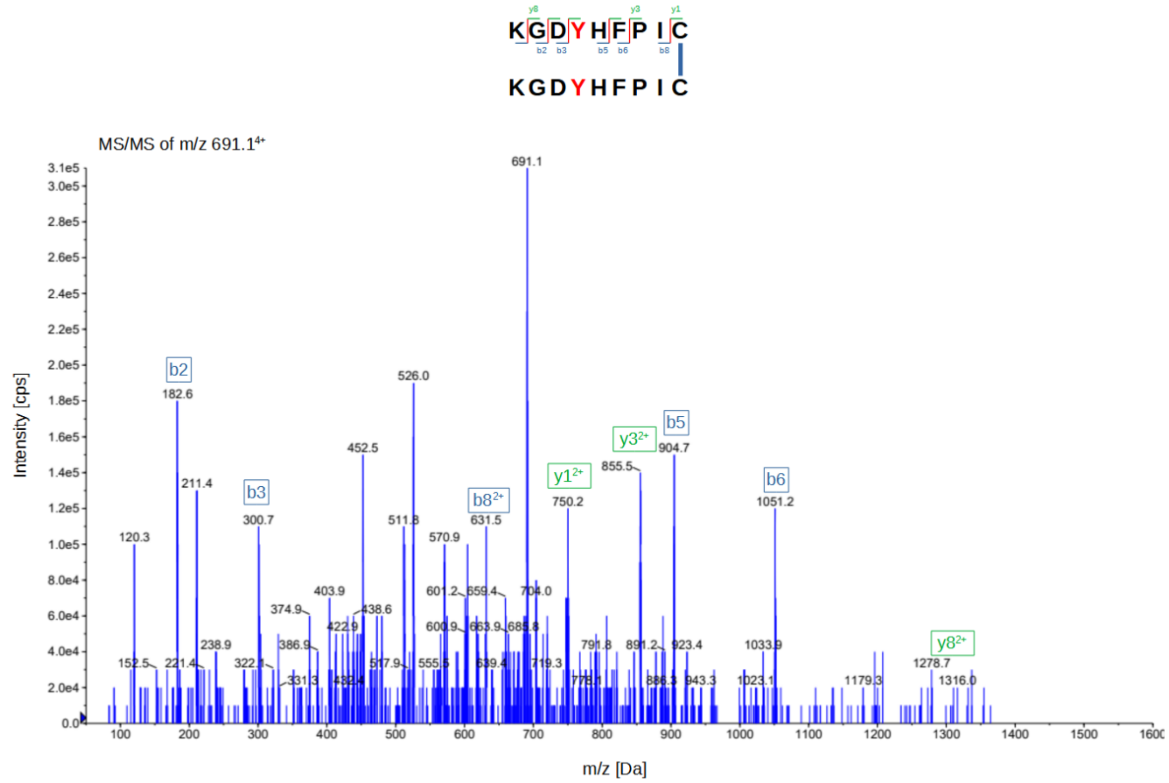
KGDYHFPIC
KGDYHFPIC
MS/MS of m/z 691.1^4+
Intensity [cps]
m/z [Da]
b2
182.6
b3
300.7
526.0
452.5
691.1
b8^2+
631.5
y1^2+
750.2
y3^2+
855.5
b5
904.7
b6
1051.2
y8^2+
1278.7

NP+16

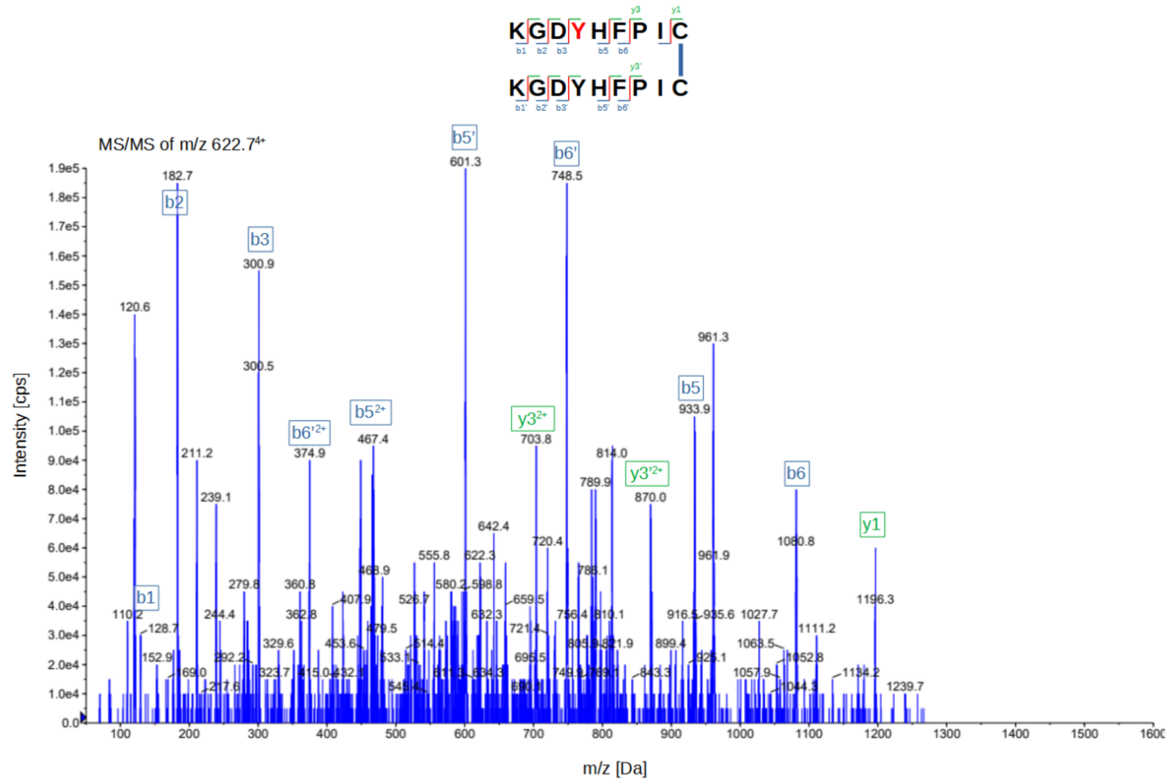
KGDYHFPIC
KGDYHFPIC
MS/MS of m/z 622.7^4+
Intensity [cps]
m/z [Da]
b1
b2
182.7
b3
300.9
120.6
b6'^2+
374.9
b5^2+
467.4
b5'
601.3
b6'
748.5
y3^2+
703.8
y3'^2+
870.0
b5
933.9
961.3
b6
1080.8
y1
1196.3

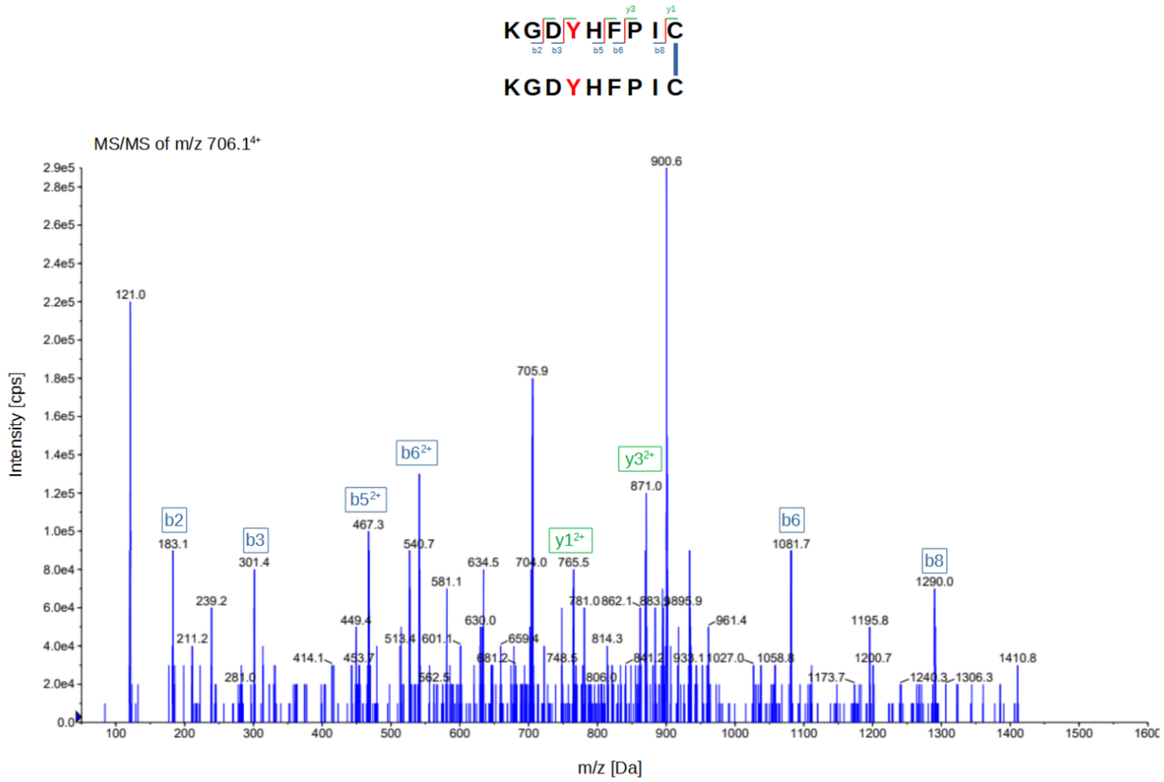
KGDYHFPIC
KGDYHFPIC
MS/MS of m/z 706.1^{4+}
Intensity [cps]
m/z [Da]
b2
b3
b5^{2+}
b6^{2+}
y1^{2+}
y3^{2+}
b6
b8
121.0
183.1
301.4
467.3
540.7
705.9
765.5
871.0
900.6
1081.7
1290.0

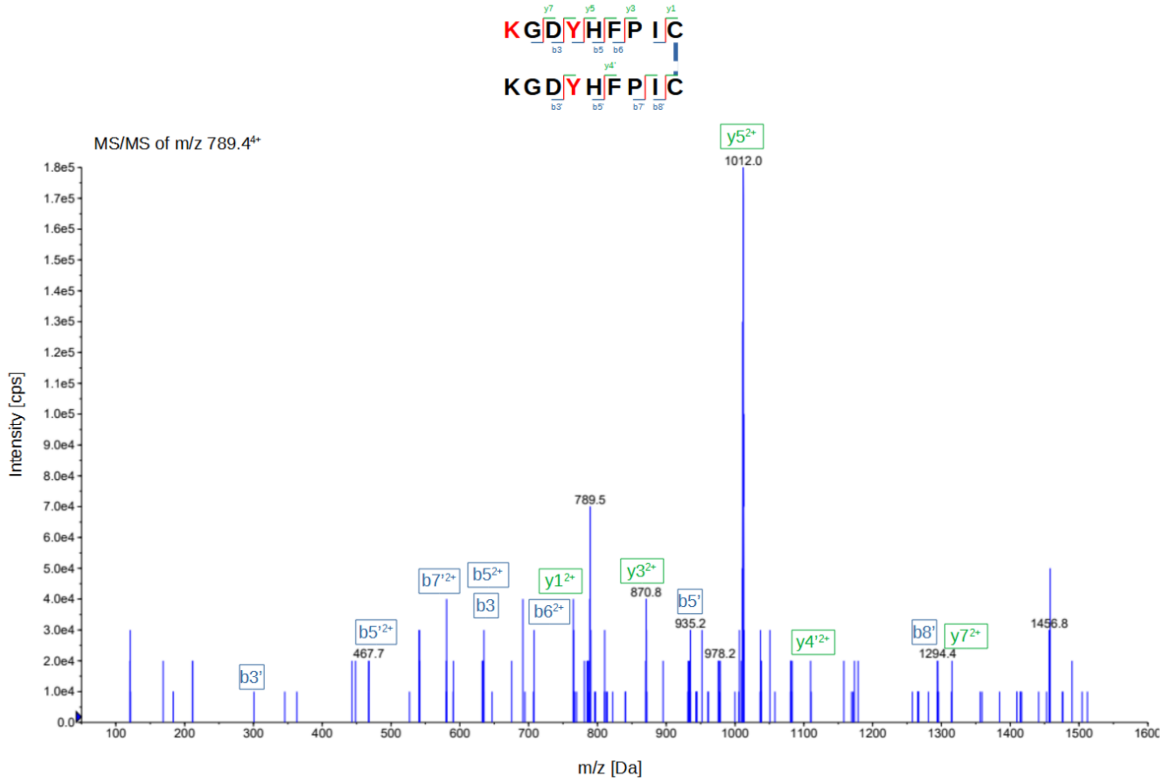
KGDYHFPIC
KGDYHFPIC
MS/MS of m/z 789.4^{4+}
Intensity [cps]
m/z [Da]
b3'
b5'$^{2+}$
b7'$^{2+}$
b5^{2+}
b3
b6^{2+}
y1^{2+}
y3^{2+}
b5'
y5^{2+}
y4'$^{2+}$
b8'
y7^{2+}
467.7
789.5
870.8
935.2
978.2
1012.0
1294.4
1456.8

NP+17

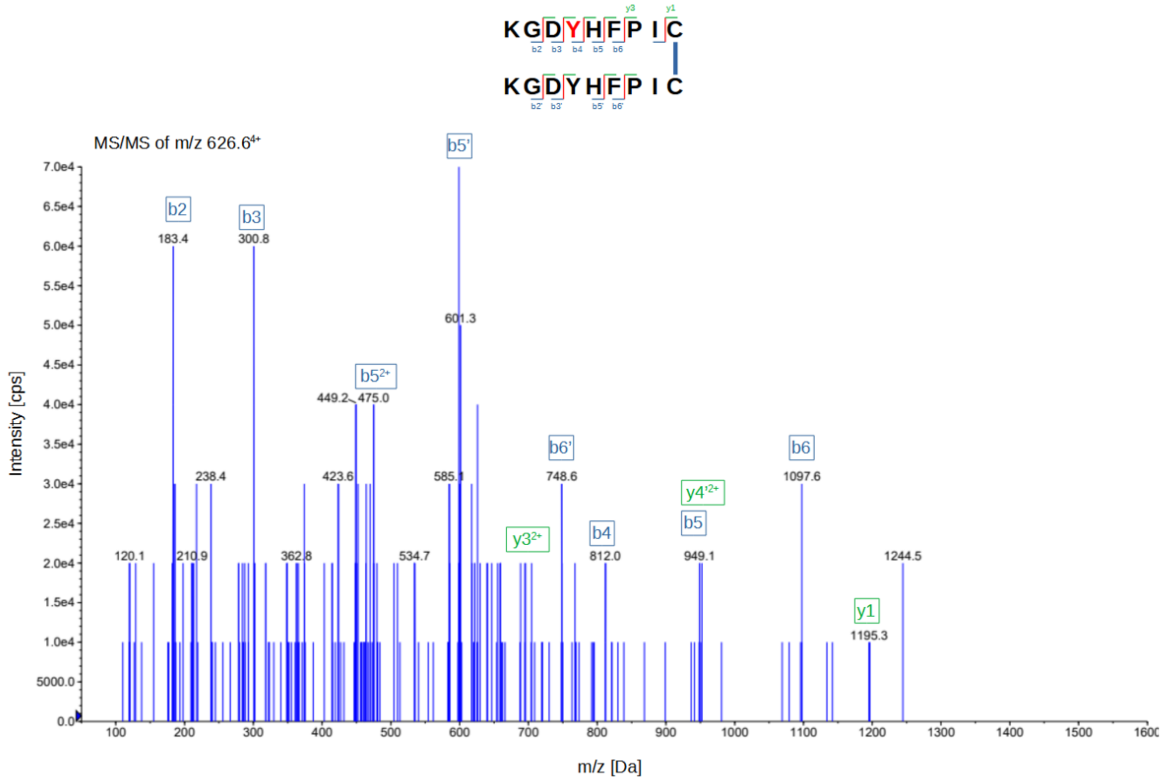
KGDYHFPIC
KGDYHFPIC
MS/MS of m/z 626.6⁴⁺
Intensity [cps]
m/z [Da]
b2
183.4
b3
300.8
b5'
601.3
b5²⁺
449.2
475.0
238.4
423.6
585.1
b6'
748.6
b6
1097.6
y4'²⁺
b5
949.1
y3²⁺
b4
812.0
120.1
210.9
362.8
534.7
1244.5
y1
1195.3

NP+18

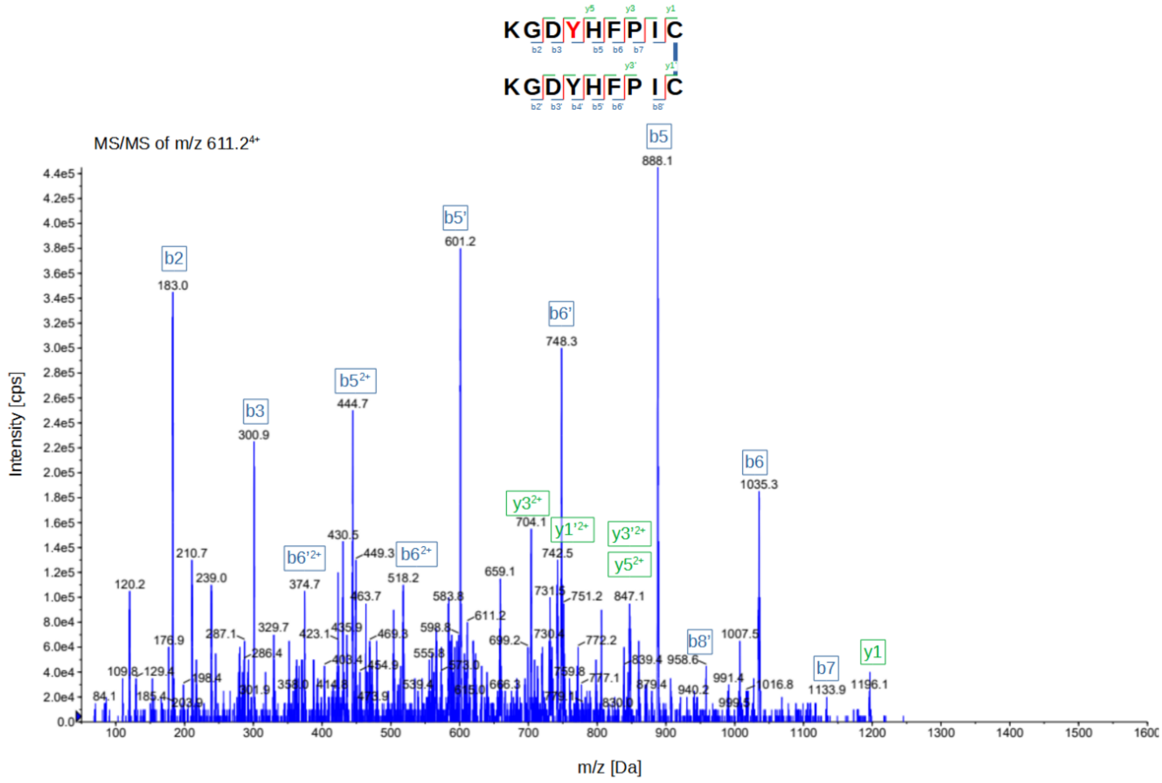
KGDYHFPIC
KGDYHFPIC
MS/MS of m/z 611.2⁴⁺
Intensity [cps]
m/z [Da]
b5
888.1
b5'
601.2
b2
183.0
b6'
748.3
b5²⁺
444.7
b3
300.9
b6
1035.3
y3²⁺
704.1
y1'²⁺
742.5
y3'²⁺
y5²⁺
b6'²⁺
374.7
b6²⁺
449.3
518.2
430.5
210.7
239.0
120.2
659.1
b8'
1007.5
b7
1133.9
y1
1196.1

NP+19

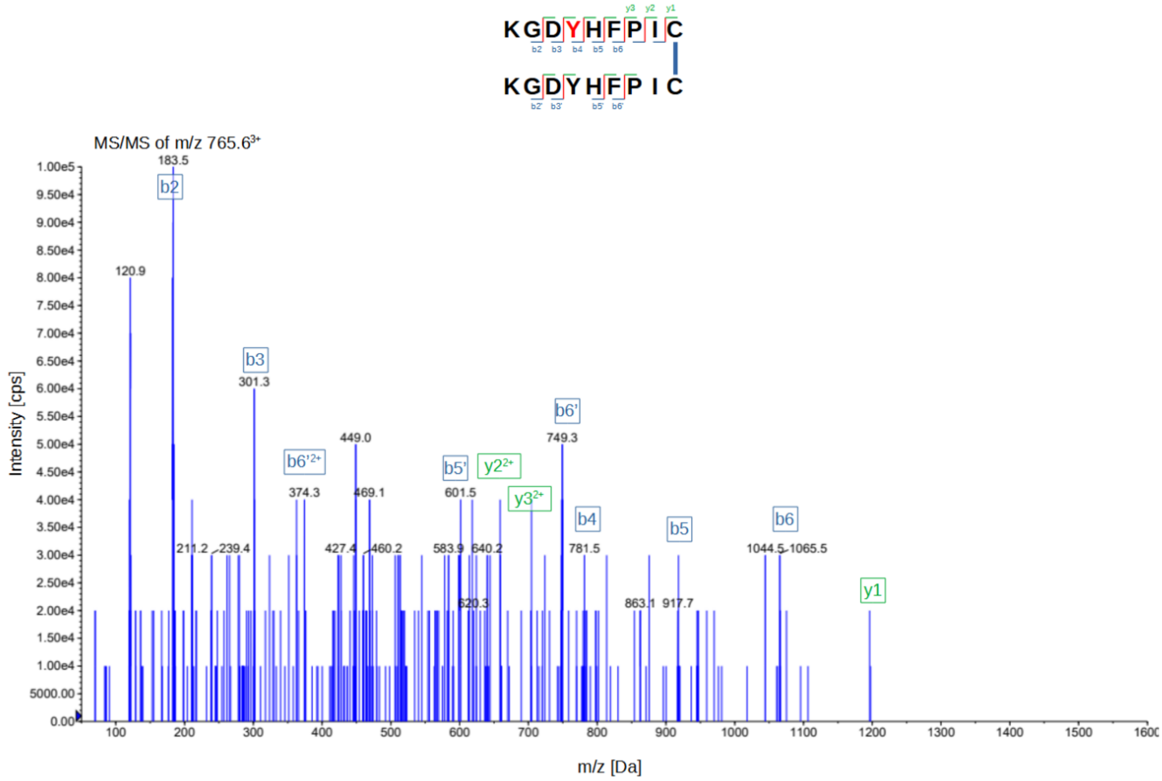
KGDYHFPIC
KGDYHFPIC
MS/MS of m/z 765.6³⁺
Intensity [cps]
m/z [Da]
b2 183.5
120.9
b3 301.3
b6'²⁺ 374.3
449.0
469.1
b5' 601.5
y2²⁺
y3²⁺
b6' 749.3
b4 781.5
b5 917.7
b6 1065.5
1044.5
y1
211.2
239.4
427.4
460.2
583.9
640.2
620.3
863.1

NP+20

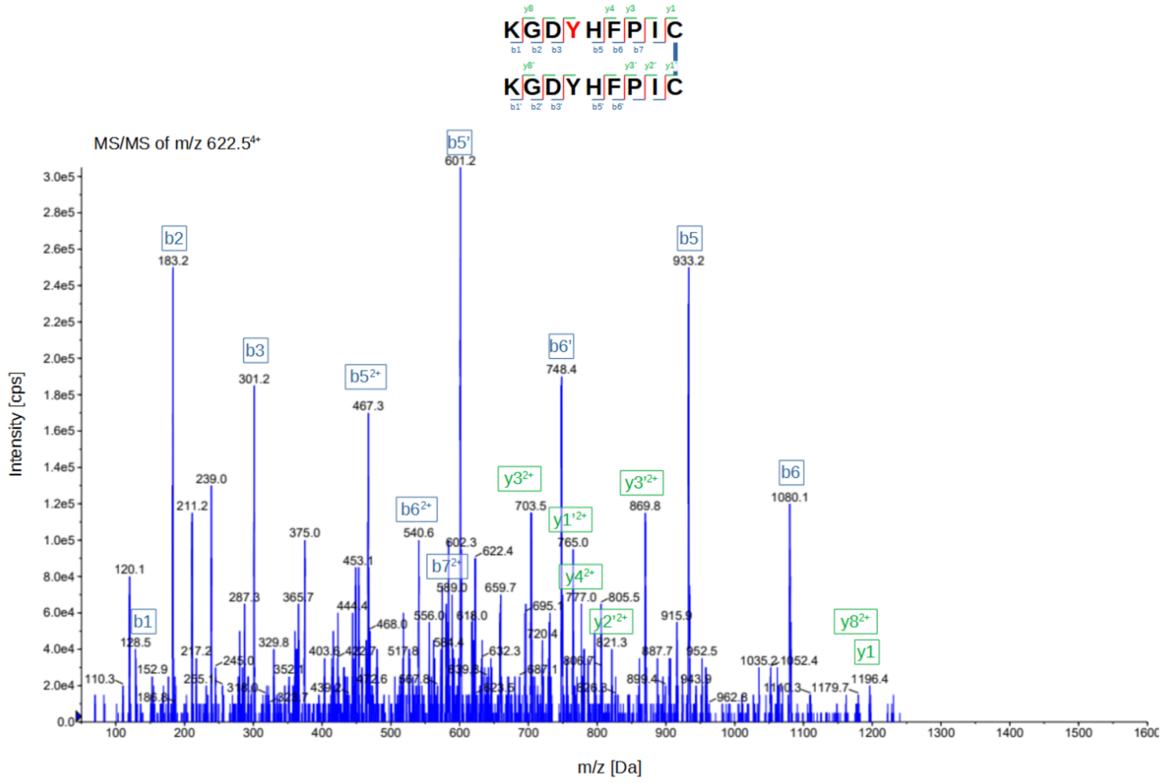
KGDYHFPIC
KGDYHFPIC
MS/MS of m/z 622.5⁴⁺
Intensity [cps]
m/z [Da]
b5' 601.2
b2 183.2
b5 933.2
b3 301.2
b6' 748.4
b5²⁺ 467.3
b6²⁺ 540.6
b7²⁺
y3²⁺ 703.5
y3'²⁺ 869.8
y1'²⁺ 765.0
y4²⁺
y2'²⁺ 821.3
b6 1080.1
y8²⁺
y1 1196.4
b1 128.5
120.1
239.0
211.2
375.0
453.1
602.3
622.4
659.7
287.3
365.7
805.5
915.9
1035.2
1052.4
1179.7
110.3

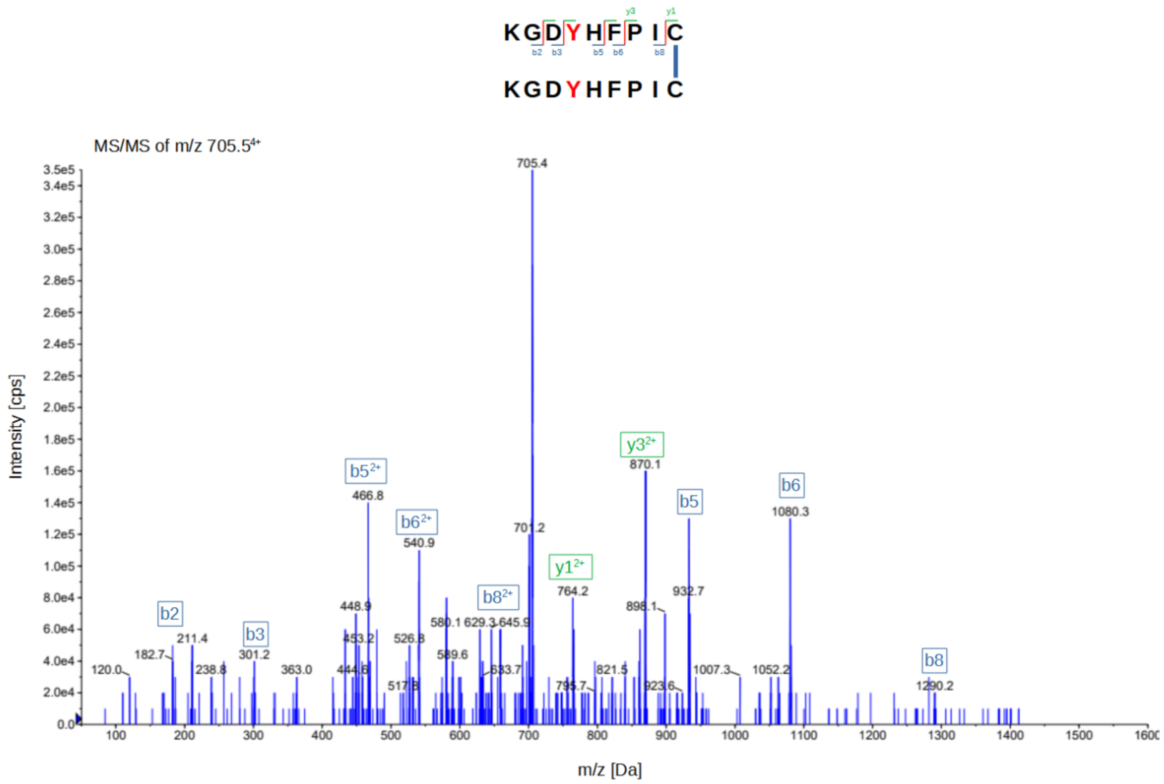

Table S2. Peptide mapping analysis for KRas^{G12D} labelled with 12 and 14 probes

Covalent probe	Modified Peptides	Position	RT (min)	Calculated Peptide Mass (Da)	Detected *m/z*	Measured Mass (Da)	Mass Error (ppm)
12	QGVDDAFYTLVR	150-161	47.5	1655.7290	828.8702	1655.7245	-2.7
	TRQGVDDAFYTLVR	148-161	40.1	1912.8778	638.6304	1912.8673	-5.5
14	QGVDDAFYTLVR	150-161	47.8	1700.7140	851.3618	1700.7078	-3.6
	TRQGVDDAFYTLVR	148-161	40.4	1957.8628	653.6249	1957.8511	-6.0

Figure S3. Mass spectrum of KRas^{G12D} 150-161 tryptic peptide modified with probe 12 and 14

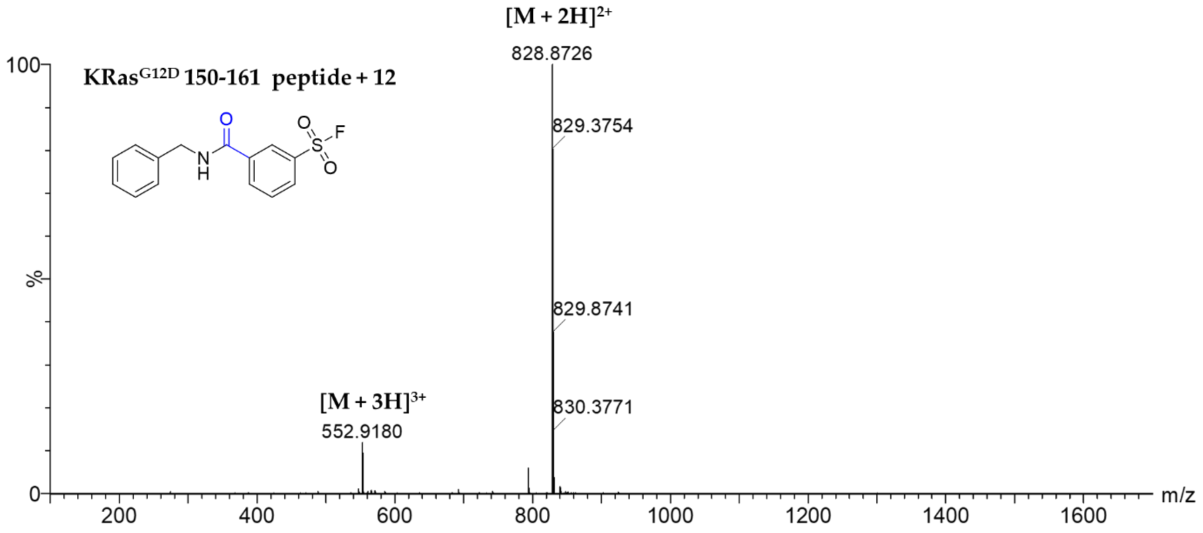

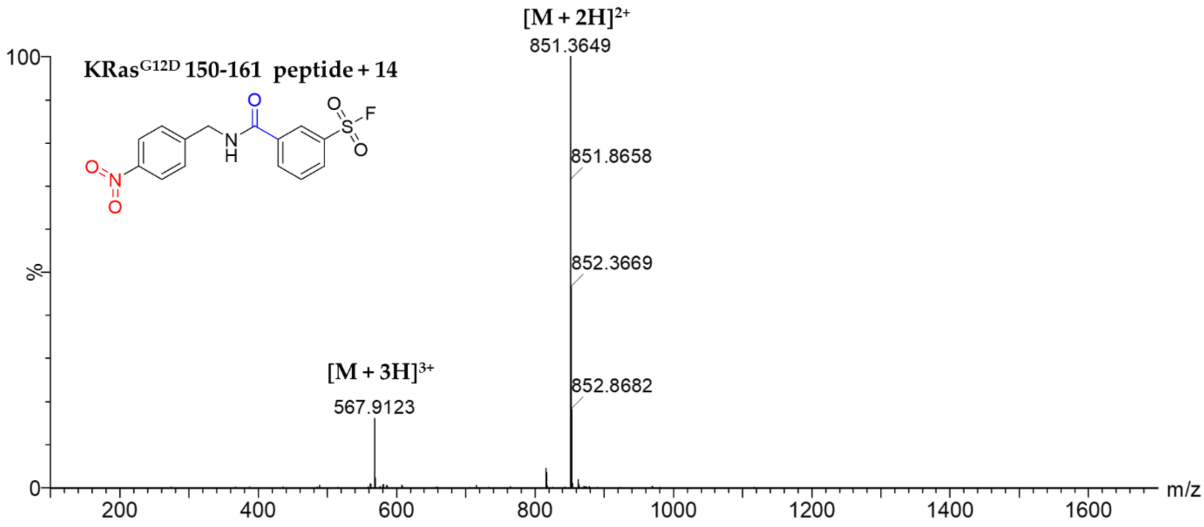

Figure S4. Mass spectrum of KRasG12D 150-161 tryptic peptide modified with probe 14

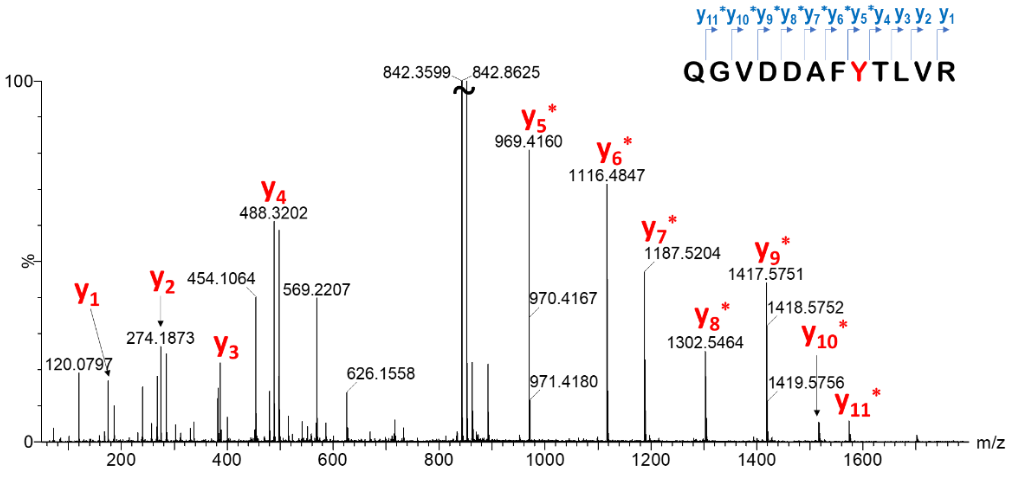

Table S3. Results of PANC-1 lysate labelling experiments analysed by mass spectrometry

Protein		Labelling site
Name	Uniprot	
40S ribosomal protein S16	P62249	N-term
60S ribosomal protein L14	P50914	K79
40S ribosomal protein S14	P62263	K106
Actin, cytoplasmic 1	P60709	Y53; Y240
Vimentin	P08670	Y30; Y38
Heterogeneous nuclear ribonucleoproteins A2/B1	P22626	T176; Y324; Y336
X-ray repair cross-complementing protein 6	P12956	S222
T-complex protein 1 subunit delta	P50991	T69
Heterogeneous nuclear ribonucleoprotein A1	P09651	Y357; S365 / Y366
60S ribosomal protein L4	P36578	Y122
60S ribosomal protein L3	P39023	Y307
40S ribosomal protein S3a	P61247	S154
60S ribosomal protein L8	P62917	Y133
60S ribosomal protein L7	P18124	Y82
60S ribosomal protein L34	P49207	S17
60S ribosomal protein L17	P18621	Y4
60S ribosomal protein L28	P46779	Y25
60S ribosomal protein L36	Q9Y3U8	Y5

Supplementary Methods

General

Reagents and solvents were purchased from commercial suppliers and were used without further purification. The reactions were monitored using thin layer chromatography (TLC) (which was carried out on Merck aluminum sheets, silica gel 60 F_{254}) or HPLC-MS (Shimadzu LCMS2020). Flash column chromatography was carried out on Teledyne ISCO Combi Flash Lumen+ Rf using silica gel 60 F_{254}. The NMR experiments were performed at 500 MHz on a Varian VNMR SYSTEM spectrometer for ^{1}H and 126 MHz for ^{13}C.

Synthesis of 2-oxo-2H-chromen-7-yl acetate (S1)

To a solution of 7-hydroxycoumarin (5 mmol, 1 eq.) in ethyl-acetate (10 ml) were added pyridine (1.1 eq.) and acetic anhydride (3.7 eq.). The resulting mixture was stirred for 2 h at room temperature. The reaction was quenched with water (20 ml) and stirred for further 30 min. The aqueous phase was separated and extracted with ethyl-acetate (3x10 ml). The combined organic phases were washed with 1M aq. HCl, saturated $NaHCO_3$ solution, water and brine then dried on Na_2SO_4. After filtration the solvent was evaporated resulting the product (**S1**) (0,961 g, 95%) as a white solid which doesn't required further purification. ^{1}H NMR (500 MHz, $CDCl_3$) δ=7.68 (dd, *J* = 9.5, 0.7 Hz, 1H), 7.48 (d, *J* = 8.4 Hz, 1H), 7.12 (d, *J* = 2.2 Hz, 1H), 7.06 (dd, *J* = 8.5, 2.2 Hz, 1H), 6.39 (d, *J* = 9.6 Hz, 1H), 2.34 (s, 3H). [43]

Synthesis of 7-hydroxy-2-oxo-2H-chromene-8-carbaldehyde (S2)

The solution of (**S1**) (4.7 mmol, 1 eq.) and trifluoroacetic acid (6,4 ml) was cooled down to 0 °C and hexamethylenetetramine (7.05 mmol, 1.5 eq.) was added. The resulting mixture was refluxed for 16 h. After completion of the reaction the TFA was evaporated under vacuum then 20 ml of water was added to the residue. The resulting mixture was stirred at 60 °C for 30 min then cooled down to 20 °C. The yellow precipitate was filtered off and dried in high vacuum to give **S2** (0,191 g, 21%). ^{1}H NMR (500 MHz, DMSO-d_6) δ 11.89 (s, 1H), 10.44 (s, 1H), 8.02 (d, J = 9.6 Hz, 1H), 7.87 (d, J = 8.8 Hz, 1H), 6.96 (d, J = 8.7 Hz, 1H), 6.38 (d, J = 9.6 Hz, 1H). [44]

Synthesis of ethyl 4-((tert-butyldiphenylsilyl)oxy)benzoate (S3)

Tert-butylchlorodiphenylsilane (5,3 mmol, 1.1 eq.) was added dropwise to the mixture of ethyl 4-hydroxybenzoate (4.81 mmol, 1 eq.) and diisopropylethylamine (5.78 mmol, 1.2 eq.) in chloroform (20 ml). The resulting mixture was stirred at room temperature for 5 h then quenched with water (20 ml). The aqueous phase was washed with chloroform (2x10 ml). The combined organic phases were dried on Na_2SO_4. After evaporation of the chloroform the crude product was purified by normal-phase flash chromatography using hexane/ethyl-acetate 0-10% eluent to furnish the desired product (**S3**) (0,788 g, 41%) as a pale yellow oil. ^{1}H NMR (500 MHz, $CDCl_3$) δ 7.82 – 7.79 (m, 2H), 7.72 – 7.68 (m, 4H), 7.45 – 7.41 (m, 2H), 7.39 – 7.35 (m, 4H), 6.79 – 6.76 (m, 2H), 4.30 (q, J = 7.1 Hz, 2H), 1.33 (t, J = 7.1 Hz, 3H), 1.11 (s, 9H). [45]

Syntesis of (4-((tert-butyldiphenylsilyl)oxy)phenyl)methanol (S4)

Under argon diethyl ether (5 ml) was cooled down to -10 °C then $LiAlH_4$ (1.87 mmol, 2 eq.) was added. To the resulting mixture (**S3**) (0.93 mmol, 1 eq.) in DEE (3 ml) was added dropwise via syringe in 10 minutes then stirred for 1,5 h keeping the temperature below 0 °C. The reaction was quenched with saturated Na_2SO_4 solution (10 ml) at 0 °C and warmed up to room temperature. The resulting white precipitate was filtered off and washed with cold water following by ethyl-acetate. The aqueous phase was separated and washed with ethyl-acetate (3x10 ml). The combined organic phases were extracted with brine, separated and dried on Na_2SO_4 then evaporated in vacuum. The resulting white solid (**S4**) (0,271 g, 81 %) required no further purification. ^{1}H NMR (500 MHz, $CDCl_3$) δ 7.73 – 7.69 (m, 4H), 7.44 – 7.40 (m, 2H), 7.38 – 7.34 (m, 4H), 7.11 – 7.07 (m, 2H), 6.78 – 6.72 (m, 2H), 4.54 (s, 2H), 1.10 (s, 9H). [46]

Synthesis of tert-butyl(4-(chloromethyl)phenoxy)diphenylsilane (S5)

Compound **S4** (0.60 mmol, 1 eq.) was dissolved in dichloromethane (5 ml) and cooled to 0 °C. Thionyl chloride (0.1 ml, 2,1 eq.) was added dropwise to the solution. The resulting mixture was warmed up to room temperature then stirred for 2 h. The mixture was poured on ice then extracted with EtOAc (3x5 ml). The organic layers were combined and washed with saturated aqueous solution of $NaHCO_3$ and brine, dried over Na_2SO_4, and concentrated in vacuo. The product (**S5**) was obtained as a white solid (0.114 g, 50 %) and was used without further purification. ^{1}H-NMR (500 MHz, $CDCl_3$): δ 7.73 – 7.68 (m, 4H), 7.45 – 7.40 (m, 2H), 7.37 (dd, J = 7.9, 6.5 Hz, 4H), 7.13 – 7.09 (m, 2H), 6.75 – 6.71 (m, 2H), 4.48 (s, 2H), 1.10 (s, 9H). [47]

Synthesis of 7-((4-((tert-butyldiphenylsilyl)oxy)benzyl)oxy)-2-oxo-2H-chromene-8-carbaldehyde (S6)

Under argon tert-butyl(4-(chloromethyl)phenoxy)diphenylsilane (**S5**) (0.85 mmol, 1.23 eq.), 7-hydroxy-8-formylcoumarin (**2**) (0.69 mmol, 1 eq.), KI (1.71 mmol, 2.47 eq.) and K_2CO_3 (6.83 mmol, 9.88 eq.) were dissolved in dry DMF (6 ml) and stirred at room temperature for 16h. The mixture was diluted with

water then the aqueous phase was extracted with EtOAc (3x10 ml). The combined organic phases were dried on Na_2SO_4 then evaporated in vacuum. The crude product was purified by normal-phase flash chromatography using hexane/ethyl-acetate 0-50% eluent to furnish the desired product (**S6**) (0.125 g, 34%) as an off-white solid. ^{1}H NMR (500 MHz, $CDCl_3$) δ 10.64 (s, 1H), 7.71 (dt, J = 6.8, 1.5 Hz, 4H), 7.61 (d, J = 9.5 Hz, 1H), 7.54 (d, J = 8.8 Hz, 1H), 7.45 – 7.40 (m, 2H), 7.39 – 7.34 (m, 4H), 7.20 – 7.15 (m, 2H), 6.93 (d, J = 8.8 Hz, 1H), 6.81 – 6.77 (m, 2H), 6.32 (d, J = 9.6 Hz, 1H), 5.14 (s, 2H), 1.10 (s, 9H). [31]

Synthesis of 7-((4-((tert-butyldiphenylsilyl)oxy)benzyl)oxy)-8-(difluoromethyl)-2H-chromen-2-one (DPF sensor: S7)

Under argon (**S6**) (0.24 mmol, 1 eq.) was dissolved in DCM (5 ml), cooled down to -20 °C then N,N-diethylaminosulfur trifluoride (DAST, 0.40 mmol, 1.7 eq.) was added dropwise via a syringe. The resulting mixture was warmed up to -5 °C and stirred for 4 h. After completion of the reaction the mixture was cooled to -78 °C and quenched with water (10 ml). The resulting solution was warmed up to room temperature and extracted with dichloromethane (2x5 ml). The combined organic phases were dried on Na_2SO_4 and evaporated in vacuum. The crude product was purified by normal-phase flash chromatography using hexane/ethyl-acetate 0-35% eluent to furnish the desired DPF sensor (**S7**) (0.035 g, 27%) as an off-white solid. ^{1}H NMR (500 MHz, $CDCl_3$) δ 7.74 – 7.70 (m, 4H), 7.60 (d, J = 9.6 Hz, 1H), 7.49 – 7.41 (m, 3H), 7.40 – 7.34 (m, 4H), 7.27 (s, 1H), 7.18 – 7.13 (m, 2H), 6.90 (d, J = 8.8 Hz, 1H), 6.81 – 6.77 (m, 2H), 6.29 (d, J = 9.5 Hz, 1H), 5.10 (s, 2H), 1.12 (s, 9H). ^{13}C NMR (126 MHz, $CDCl_3$) δ 159.48, 155.76, 143.00, 135.49, 132.73, 131.22, 129.94, 128.44, 127.80, 127.78, 119.99, 113.92, 112.82, 110.01, 109.47, 71.01, 26.50, 19.45.

Supplementary References

24. Mukherjee, H.; Debreczeni, J.; Breed, J.; Tentarelli, S.; Aquila, B.; Dowling, J.E.; Whitty, A.; Grimster, N.P. A Study of the Reactivity of S (VI) –F Containing Warheads with Nucleophilic Amino-Acid Side Chains under Physiological Conditions. *Org Biomol Chem* **2017**, *15*, 9685–9695, doi:10.1039/C7OB02028G.

31. Gu, J.-A.; Mani, V.; Huang, S.-T. Design and Synthesis of Ultrasensitive off–on Fluoride Detecting Fluorescence Probe via Autoinductive Signal Amplification. *Analyst* **2015**, *140*, 346–352, doi:10.1039/C4AN01723D

43. Álvarez-Calero, J.M.; Jorge, Z.D.; Massanet, G.M. TiCl $_4$ /Et $_3$ N-Mediated Condensation of Acetate and Formate Esters: Direct Access to β-Alkoxy- and β-Aryloxyacrylates. *Org Lett* **2016**, *18*, 6344–6347, doi:10.1021/acs.orglett.6b03233.

44. Zhu, G.; Huang, Y.; Wang, C.; Lu, L.; Sun, T.; Wang, M.; Tang, Y.; Shan, D.; Wen, S.; Zhu, J. A Novel Coumarin-Based Fluorescence Chemosensor for Al3+ and Its Application in Cell Imaging. *Spectrochim Acta A Mol Biomol Spectrosc* **2019**, *210*, 105–110, doi:10.1016/j.saa.2018.11.006.

45. Guggilapu, S.D.; Prajapti, S.K.; Babu, B.N. An Efficient One-Pot Oxidative Esterification of Aldehydes to Carboxylic Esters Using B(C6F5)3–TBHP. *Tetrahedron Lett* **2015**, *56*, 889–892, doi:10.1016/j.tetlet.2015.01.033.

46. Gangar, M.; Harikrishnan, M.; Goyal, S.; Mungalpara, M.N.; Nair, V.A. A Highly Efficient and Enantioselective Synthesis of EEHP and EMHP: Intermediates of PPAR Agonists. *Tetrahedron Lett* **2016**, *57*, 3462–3467, doi:10.1016/j.tetlet.2016.06.093.

47. Yamamoto, J.; Maeda, N.; Komiya, C.; Tanaka, T.; Denda, M.; Ebisuno, K.; Nomura, W.; Tamamura, H.; Sato, Y.; Yamauchi, A.; et al. Development of a Fluoride-Responsive Amide Bond Cleavage Device That Is Potentially Applicable to a Traceable Linker. *Tetrahedron* **2014**, *70*, 5122–5127, doi:10.1016/j.tet.2014.05.110.
